# A Genome-Wide Analysis of Genetic Diversity in *Trypanosoma cruzi* Intergenic Regions

**DOI:** 10.1371/journal.pntd.0002839

**Published:** 2014-05-01

**Authors:** Leonardo G. Panunzi, Fernán Agüero

**Affiliations:** 1 Instituto de Investigaciones Biotecnológicas – Instituto Tecnológico de Chascomús, Universidad de San Martín – CONICET, Sede San Marítn, Buenos Aires, Argentina; Karolinska Institutet, Sweden

## Abstract

**Background:**

*Trypanosoma cruzi* is the causal agent of Chagas Disease. Recently, the genomes of representative strains from two major evolutionary lineages were sequenced, allowing the construction of a detailed genetic diversity map for this important parasite. However this map is focused on coding regions of the genome, leaving a vast space of regulatory regions uncharacterized in terms of their evolutionary conservation and/or divergence.

**Methodology:**

Using data from the hybrid CL Brener and Sylvio X10 genomes (from the TcVI and TcI Discrete Typing Units, respectively), we identified intergenic regions that share a common evolutionary ancestry, and are present in both CL Brener haplotypes (TcII-like and TcIII-like) and in the TcI genome; as well as intergenic regions that were conserved in only two of the three genomes/haplotypes analyzed. The genetic diversity in these regions was characterized in terms of the accumulation of indels and nucleotide changes.

**Principal Findings:**

Based on this analysis we have identified i) a core of highly conserved intergenic regions, which remained essentially unchanged in independently evolving lineages; ii) intergenic regions that show high diversity in spite of still retaining their corresponding upstream and downstream coding sequences; iii) a number of defined sequence motifs that are shared by a number of unrelated intergenic regions. A fraction of indels explains the diversification of some intergenic regions by the expansion/contraction of microsatellite-like repeats.

## Author Summary

Chagas disease is caused by the protozoan parasite *Trypanosoma cruzi*, and poses a serious public health problem in the America, with approximately 8 million people infected and 200,000 new cases reported annually. The disease has different clinical manifestations. The fact that infections by the same species cause different clinical outcomes is believed to be determined, at least in part, by the genetic background of the parasite (infection by different strains). Previous characterizations of the genetic diversity in *Trypanosoma cruzi* were carried out on the protein-coding portions of the genome. However, the genetic diversity of non-coding intergenic regions remained unexplored. These regions are particularly important in trypanosomes because they contain essential regulatory sequences that drive the process of mRNA maturation and that ultimately govern the expression of genes. In this study, we analyzed the genetic diversity present in non-coding regions of the genome, and provide a broad picture of the selective forces acting on this subset of the genome. Based on this analysis we identified a highly conserved core of intergenic regions, that were maintained essentially unchanged over large evolutionary periods of time, as well as a highly divergent set of intergenic regions.

## Introduction

Understanding the functional significance of noncoding DNA is one of the major challenges of current genomics research. Although a number of early studies suggested that noncoding DNA evolve largely free from selective constraints, recent genome-wide comparative studies in higher eukaryotes showed that some noncoding DNA, particularly introns, are subjected to significant evolutionary pressures [1]–[3].

Kinetoplastid protozoa's genes are essentially introns-free and predominantly arranged in long constitutively transcribed polycistronic units [4], [5] that are subsequently processed to produce mature monocistronic mRNAs by coupled reactions of *trans*-splicing of a small 39 nt spliced leader (SL) sequence to the 5′- end, and polyadenylation of the 3′ end [6]. Therefore, the intergenic region contains intron-like features such as polypyrimidine tracts and (*trans-*)splicing acceptor sites that drive the maturation of mRNAs. Evenmore, noticeable regulatory DNA sequence motifs are absent as well, suggesting a major role of post-transcriptional mechanisms in the regulation of mRNA abundance [7].

Trans-acting factors (RNA-binding proteins and other cofactors) selectively destabilize mRNAs [5] and regulate their abundance, by binding to structural RNA motifs in untranslated regions [8], [9]. In other organisms, it has also been shown that functionally related mRNAs are co-expressed by the action of RNA-binding proteins [10]. In trypanosomes, gene expression could be explained by post-transcriptional RNA regulons, composed of sets of mRNAs sharing regulatory motifs [11]–[15]. Alternative RNA processing sites also suggest the existence of other dynamic mechanisms affecting the presence of regulatory elements in transcripts [16], [17].

Given their importance in the regulation of gene expression in trypanosomes, we were interested in analyzing the genetic diversity and apparent selection in intergenic and untranslated regions of *T. cruzi*. Previous studies have described their composition and sequence features [18], [19], but none have focused on their diversity or degree of conservation. Recently, we and others have analyzed a number of complete *T. cruzi* genomes and characterized the genetic diversity of their protein coding regions [20], [21]. However, an important part of the genome was left uncharacterized in terms of their diversity and the apparent selective pressure affecting these noncoding regions.

*T. cruzi*, the aetiological agent of Chagas disease, is a vector-borne infection with a high prevalence in Central and South America [22]–[24]. Its reproduction is mostly clonal, with almost no genetic recombination [25], [26]. During its evolutionary history a highly structured population, currently composed of six major lineages, or Discrete Typing Units (DTUs) TcI to TcVI [27]–[29], was produced. Despite this predominant clonality, there is evidence of composite (TcIII and TcIV) and hybrid (TcV and TCVI) genomes [30]–[33]. Hybrid lineages have alleles from ancestral TcII and TcIII haplotypes, whereas the ancestry of composite lineages is not yet fully understood. The remarkable genetic heterogeneity of these divergent evolving groups could partially account for their wide range of biological features, eco-epidemiological traits [34], [35], and the large spectrum of clinical manifestations of Chagas disease [36], [37].

During its complex life cycle, alternating between an insect and a mammalian vertebrate host, *T. cruzi* must quickly adapt to diverse environments, while undergoing dramatic morphological and functional changes [38]. This feat is dependent on drastic changes on gene expression [39], [40].

The genome sequence of the *T. cruzi* hybrid CL Brener (TcVI) strain [41] evinced two divergent haplotypes (TcII-like and TcIII-like). Each haploid genome was comprised of 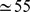 Mb that harbors 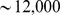 protein-coding genes located in 40 chromosomes, with at least 30–50% of repetitive sequences [42], [43]. Recently, the non-hybrid Sylvio X10 strain (TcI) was sequenced and it revealed a conserved core of genes and a significant reduction of large gene families, which explains the smaller genome size (44 Mb) [20]. This comparative genome analysis was focused on protein coding regions, but no further inquiry was done on intergenic and/or putative untranslated regions.

In this paper, we have extended our analysis of genetic diversity to intergenic regions (IGRs) in order to provide a detailed comparative characterization of allelic variants of extant genomes. As a result we found a core of highly conserved IGRs, a considerable number of divergent IGRs, and a number of IGR segments of variable length that are shared between unrelated genomic *loci*.

## Materials and Methods

### Genomic data source

We retrieved the analyzed sequence data from TriTrypDB (http://tritrypdb.org/tritrypdb/, version 4.0), belonging to homologous chromosome-sized scaffolds [44] from the hybrid CL Brener (TcII-like and TcIII-like haplotypes) and to contigs from the non-hybrid Sylvio X10 (TcI) *T. cruzi* strains.

### Selection of orthologous IGR regions

**Figure 1:**
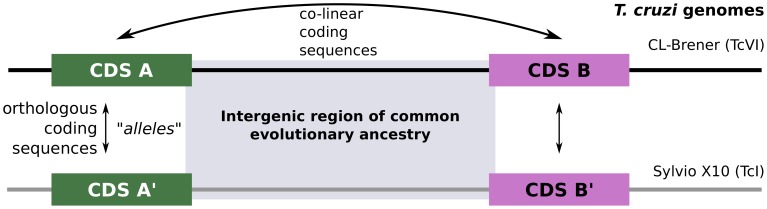
Identification of chromosomal regions of common evolutionary ancestry between *T. cruzi* genomes. Colinear coding sequences in the genomes analyzed were used to define the boundaries of the corresponding IGRs. Given a pair of colinear coding sequences A, B from one genome, if an ortholog of A in the second genome (A′) is also colinear with the ortholog of B, then the boundaries of the IGR are defined from the location of the corresponding translational START and STOP codons.

We defined the IGR unit, which was the minimum comparable genomic region in our analysis, as the sequence between two consecutive annotated coding sequences (CDSs, see Figure 1). Coding sequences upstream and downstream of a given IGR must be both colinear and orthologous in the hybrid CL Brener (TcII-like and TcIII-like alleles) and non-hybrid Sylvio X10 (TcI) genomes.

These non-coding IGR regions were extracted according to the coordinates of its adjacent consecutive CDSs, first from from CL Brener contigs and subsequently searched in the Sylvio X10 genome using BLAT [45]. Best reciprocal hits between homologous regions were selected if sequences were 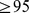
% identical. Sequences producing multiple-hits CDSs and pseudogenes were removed in order to obtain pairs of syntenic orthologous single-copy genes where the shared ancestry of IGR blocks could be safely assume. However, it is possible that near-identical copies of the same gene could have collapsed during genome assembly, therefore passing this filter.

The final curation of our dataset was achieved by filtering IGRs that match the following criteria: i) absence of ambiguous DNA bases; ii) absence of long gaps at the end of aligned flanking CDSs (presence of such gaps may suggest an inaccurate or incomplete annotation of the CDS translational start, and may induce us to include unnanotated parts of a CDS within the IGR); and iii) presence of corresponding start and stop codons at the ends of all CDS. A summary of the data analyzed is shown in Table S1. This constitutes the complete dataset of coding and non-coding DNA that was examined.

All downstream analyses were performed by a series of custom scripts written in Perl and R languages.

### Sequence analyses

We determined the nucleotide composition and length for each IGR unit in all haplotypes using standard bioinformatics tools. Sequences were aligned using different algorithms for coding (CDS) or non-coding (IGR) regions, as described below.

Alignment of coding sequences was done using MUSCLE [46], [47]. However, to align IGR regions, which contain more gaps and polymorphims, we used the MCALIGN algorithm [48], [49], that explicitly models indels based on *prior* information. To generate this prior information, we first aligned IGR regions using SIGMA [50], [51], which is designed specifically for noncoding DNA. From these data we determined the indel length frequency distribution, which was used as input to MCALIGN to obtain the final alignments of IGRs. Polymorphisms were labeled as transitions/transversions, and for each IGR we calculated the number of single nucleotide polymorphisms (SNPs) per site as a measure of sequence divergence, using the total length of the alignment as the number of sites. In the case of indels, for each IGR we produced a list of indels of different sizes and report the number of indels per site as a measure of sequence divergence.

To detect blocks of sequence similarity among unrelated (non-orthologous) IGRs, we performed all-vs-all BLASTN searches, using a local database of IGR regions extracted from all haplotypes. Using a custom Perl script we skipped orthologous IGRs in the list of BLASTN hits, and then manually analyzed the remaining hits to find significant alignments against non-orthologous IGRs.

#### Detection of repetitive DNA elements and structural RNA motifs

To detect simple repeats and longer repetitive DNA elements, we used IGR regions to search the RepBase database[52] (Release 20130422), with RepeatMasker[53] (Version 4.0.3), using a RepeatMasker compatible version of the NCBI BLAST suite (RMBlast, http://www.repeatmasker.org/RMBlast.html) as the sequence similarity search engine. RepeatMasker was run with the following parameters: ‘ -s -gccalc –nolow’. Two sets of searches were performed, one against human repeats (as control), and another against the *T. cruzi* entries in RepBase. To detect structural RNA motifs we searched the Rfam database (release 11.0) [54] using cmscan v1.1rc1 (June 2012).

### Experimental methods

#### Samples

Purified DNA samples from a representative panel of the main six *T. cruzi* DTUs (TcI-TcVI), isolated from differentially located hosts and vectors, were kindly provided by Patricio Diosque (National University of Salta, Salta, Argentina). They were genotyped using a MLST (Multilocus Sequence Typing) method [55] and were characterized further with previously described genetic markers [25], [56], and with a recently developed typing method [57]. A panel of 20 different strains, was used to amplify IGR regions. IGR regions were selected based on their total length and level of sequence diversity; information that was gathered in the analyzed data from the aligned homologous chromosomal regions among paired haplotypes. These targets were of a especially suitable length, broadly 300–400 bp, to get them fully sequenced (from any strand) in only one reaction. Some of them were also restrained to be equally long and to present a large proportion of SNPs between any pair of haplotypes, whereas other of them included not only a significant fraction of polymorphic sites but also huge INDELs. The Primer3 [58] program was used for designing PCR primers (Table S2) of all targeted genomic IGR loci, and we included an integral part of flanking CDSs in order to procure predominantly conserved regions in all haplotypes where primers matched.

#### PCR amplification

To discard genome assembly problems that might produce size differences between allelic IGRs, we selected six diverse IGR regions based on their level of sequence conservation. They were: 1) IGRs with virtually identical lengths in all haplotypes; 2) unique IGR length only in one haplotype. We performed PCR amplification followed by TBE agarose gel electrophoresis (see Table S2 and section “Probing the diversity of intergenic regions in an expanded panel of strains”). DNA samples belonged to a complete panel of strains, representative of all major DTUs, therefore we could observe the diversity of IGR sizes in these other *T. cruzi* DTUs.

Information about oligonucleotide primers related to their annealing temperature, product size, chromosomal coordinates and sequence, is given in Table S2. The PCR cycle consisted of an initial denaturation step (3 min at 94°C), primer annealing (30 sec, at specific temperatures aforementioned) and primer extension (time according to each PCR product length, at 72°C). This usual procedure was repeated 30 times and was immediately followed by a final extension step (5 min at 72°C). Each PCR reaction was produced in a total volume of 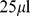 and contained: 50 ng genomic DNA, 4 mM 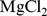, 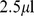 10X PCR Buffer (Invitrogen), 0.2 mM dNTPs (Invitrogen), 200 nM each forward/reverse primer (Sigma-Aldrich), 2 U Taq Polymerase (Invitrogen). PCR products were confirmed by gel electrophoresis (1.5% agarose in 1X TBE, 90 V for 45 min).

## Results and Discussion

### Overall strategy, collection of orthologous loci

Using the selection strategy described in Methods (Figure 1), we were able to retrieve 4,312, 4,642 and 2,644 IGRs that were flanked by 5,284, 5,637 and 3,886 CDSs, belonging to TcII-like, TcIII-like and TcI, respectively. These genomic regions comprise approximately 43% of the haploid megabase-sized scaffolds of the CL Brener genome, 29.3% of the contigs of the Sylvio X10 genome, and correspond to 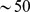
% of the annotated protein-coding genes in the reference CL Brener genome. This coverage is in agreement with current estimations of the repetitive content of the *T. cruzi* genome, estimated to be 30–50%. Another additional factor that could be responsible for filtering out *bona fide* IGRs is the quality of the assembly of current draft genomes. Notwithstanding these caveats, the fraction of the genome analyzed corresponds to a “core” of genes and their associated IGRs which have been maintained together over the evolution of the species. All this information is available as Supplementary Material (Table S3).

**Figure 2:**
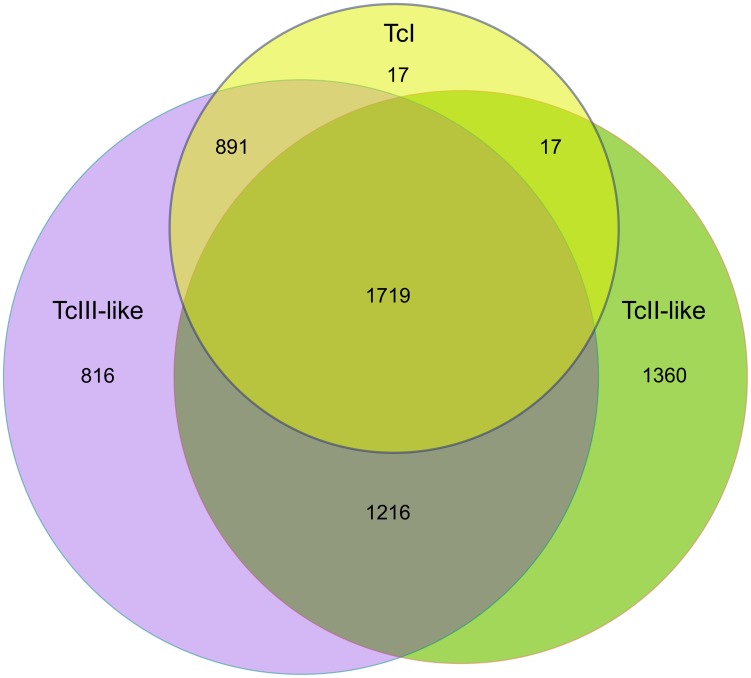
Distribution of shared evolutionary ancestry of IGRs across analyzed genomes/haplotypes. The Venn diagram shows the distribution of orthologous (allelic) IGRs in each genome/haplotype. IGRs containing assembly gaps were excluded from the intersections and are shown as unique to each haplotype. The diagram is not drawn to scale, and is only intended to highlight each subset.

After identification of these IGRs in a 3-way comparison between the two CL Brener haplotypes and the Sylvio X10 contigs, we grouped these regions according to their shared ancestry, and discarded problematic IGRs (see below). As depicted in the Venn diagram in Figure 2, we identified a total of 6,036 IGRs from the 3 haplotypes analyzed. From these, only 1,719 were strictly shared between the three haplotypes. In agreement with previous comparative analyzes of TcI vs CL Brener sequences [20], [33], [59], IGRs from the TcI genome were most often conserved when compared with the TcIII-like haplotype of CL Brener. Interestingly, the TcII-like and TcIII-like haplotypes shared 2,935 IGRs, which is close to the 2,601 IGRs shared by TcI and TcIII-like haplotypes.

For some of the characterizations reported below, we also discarded a number of IGRs from the analysis. Problematic IGRs were those that contained stretches of Ns introduced by the sequencing center/author (indicating gaps in the assembly). As such, they break our requirement of strict colinearity (Figure 1) and were not considered further. These can be observed in the Venn diagram as sets of IGRs that are apparently unique to one genome/lineage. In the case of the Sylvio X10 genome (which is not scaffolded), there were only 17 IGRs with gaps in the assembly, whereas this figure raises to 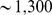 and 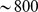 IGRs in the case of the TcII-like and TcIII-like haplotypes of CL Brener, respectively. Although these numbers may seem large, in comparison with the Sylvio X10 genome, they do not reflect an intrinsic diversity of IGR regions between these haplotypes but rather the high degree of scaffolding of the reference CL Brener genome[44] (joining two contigs together in a scaffold required the introduction of Ns to indicate an assembly gap). Based on this observation we decided to perform our analysis of genetic diversity on the set of 3,843 “good” IGRs (those present at the intersections of the Venn diagram). These represent a core set of IGRs which were maintained together as a block with their corresponding flanking coding sequences, over large periods of time.

### The highly conserved core genome of *T. cruzi*

The method described in the previous section for the identification of orthologous IGR regions allowed us to obtain groups of IGRs with shared ancestry between *T. cruzi* lineages. However, in spite of being maintained together over large evolutionary spans, further characterization of the genetic diversity present in these 1,719 IGRs showed both highly conserved IGRs, with little or no accumulation of indels and/or SNPs, and highly divergent IGRs. In this section, we describe an analysis of this diversity, decomposed into i) length differences (indels) and ii) nucleotide differences (SNPs).

Because IGR regions contain a number of essential regulatory regions, this diversity could have important functional implications. In the case of polypyrimidine (PPY) tracts (which are essential for *trans-*splicing in trypanosomes[60]–[62]), it has been demonstrated that both the base composition [63], and the length [64] of a PPY tract are important for its efficiency. Therefore changes affecting the length, composition and/or sequence of PPY tracts in allelic IGRs may be affecting the location and/or efficiency of polyadenylation and *trans-*splicing of the flanking genes.

**Figure 3:**
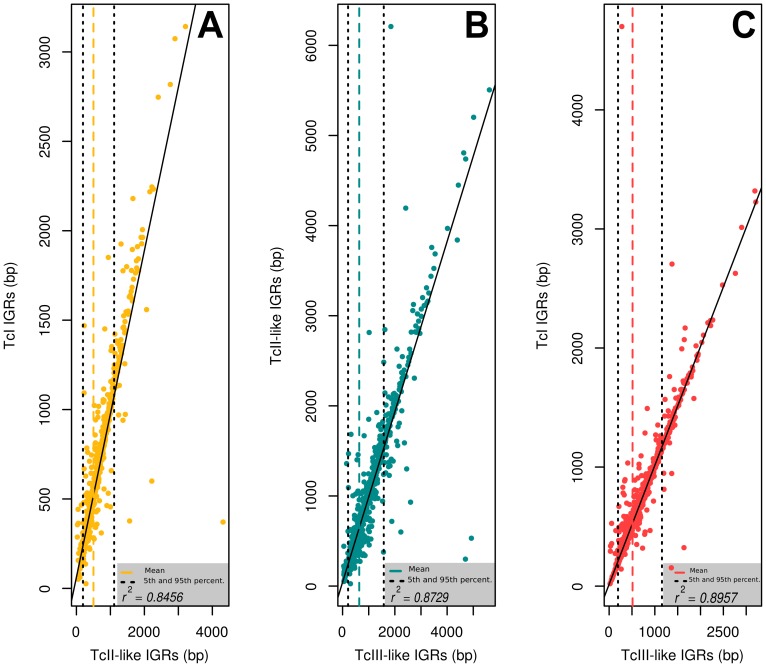
Size correlation of orthologous IGR regions. The plots show the pairwise comparisons of IGR regions between the three analyzed haplotypes/genomes: A) TcI vs TcII-like, B) TcII-like vs TcIII-like, C) TcI vs TcIII-like. The colored dotted line in each plot marks the mean value of each distribution, while the black dotted lines mark the 5th and 95th percentiles, respectively. Plot axes correspond to length (size) of the IGR region, in base pairs, for each haplotype/genome.

The conservation of the length of IGR and CDS sequences, which serves as a measure of accumulation of indels, were broadly comparable in all *T. cruzi* haplotypes. As shown in Figure 3, apart from a few outliers, there was a good conservation of the length of IGRs across genomes/haplotypes. Again, we found a higher correlation between TcI and TcIII-like haplotypes in both coding and non-coding genomic regions (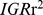: 0.8957, (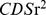: 0.997, see Supplementary File S1), followed by the remaining pairs, with lower correlation coefficients (TcI vs TcII-like, 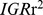: 0.8456; TcII-like vs TcIII-like, 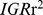: 0.8729). In all these cases the same trend is observed in CDS regions but of course with higher correlation coefficient values (Figure S1).

**Figure 4:**
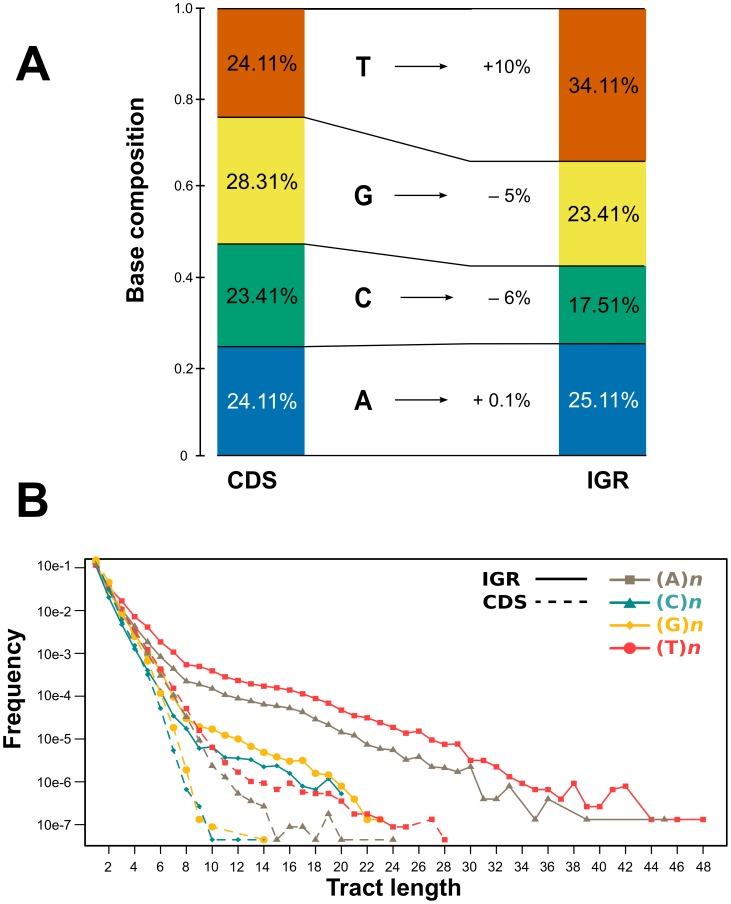
Analysis of sequence composition of core CDS and IGR regions. A) Comparison of base composition of 1,719 selected IGR regions and their associated flanking coding sequences. B) Comparison of ocurrence of homopolymer tracts in CDS vs IGR regions.

The base composition of these IGRs revealed a marked T bias (34%), and a significant underrepresentation of C (17.5%), when compared with coding sequences (24.1% of T and the 23.4% of C) (Figure 4). These two bases are essential components of regulatory motifs within IGRs (PPY tracts). In the case of short IGRs, this functional constraint alone may explain the compositional bias of IGRs. Also, the lower proportion of GC in IGR regions (40%) when compared to CDS regions (51%) could be a possible consequence of mutational and selective forces that could be favouring AT-rich regulatory motifs [65]–[67] and establish a stable molecular structure that might modulate the transcription of genes [11] and enhance the affinity for RNA-binding proteins [68]–[70].

In order to relate compositional and length features, we also surveyed the occurrence of non-overlapping homopolymer tracts in both CDS and IGR regions. We detected a higher frequency of each type of polynucleotide (dA, dC, dG and dT) in non-coding DNA, in strong contrast with coding DNA (Figure 4). Although the virtual absence of long homopolymeric tracts could be expected for coding regions, it was intriguing to see that IGR regions have a marked preference for dA and dT. This preference could have functional consequences either in the transcriptional and/or post-transcriptional regulation of gene expression. If these homopolymeric tracts are present in a mature transcript, they could bind regulatory RNA binding proteins, as described previously for *T. cruzi* [66], [68]. However, these tracts could also regulate the expression of genes by producing long nucleosome-free regions (LNFRs). In a genome-wide analysis of nucleosome occupancy and nucleosome DNA sequence preference in yeast, Kaplan *et al.* showed that AAAAA and ATATA are the most prominent patterns in LNFRs [71], and more generally, that poly(dA-dT) are good LNFR indicators, in agreement with previous observations [72]–[76]. Although in trypanosomes there are apparently no promoters regulating transcription initiation by RNA polymerases, it has been shown that there could be regulation of transcription through nucleosome and/or chromatin remodeling [77], [78].

### A closer resemblance between TcI and TcIII-like haplotypes in IGRs and CDSs

The previous comparative analysis of Sylvio X10/1 and CL Brener genomes [20] showed that the highly conserved “core” of coding regions had a lower mean nucleotide diversity between TcI and TcIII-like than TcI and TcII-like haplotypes. In our study we further expanded this analysis to non-coding IGRs. We found less nucleotide differences between TcI and TcIII-like haplotypes in both regions (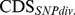: 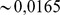, 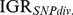: 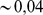) than those observed between TcI and TcII-like haplotypes (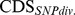: 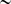 0,022, 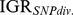: 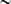 0,052) or between TcII-like and TcIII-like haplotypes (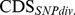: 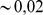, 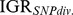: 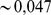) (data in Supplementary Figure S2). We also performed additional nonparametric statistical tests which evinced significant differences among possible pairwise comparisons in both IGRs and CDSs regions. The Kruskal-Wallis test (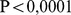, *a posteriori* test 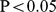) revealed disparities between the medians of these groups, while the Mann-Whitney U test (2-tailed, 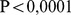) enabled testing significant differences of unique specific pairs of data.

These results confirmed the greater genetic diversity between TcI and TcII-like haplotypes in a curated and representative sample of 1,719 IGRs, and 2,648 CDS. This finding is in agreement with the current proposed evolutionary relationships among DTUs [30], [31], [33], [59] and the longer estimated divergence time between TcI and TcII haplotypes.

### Dissection of genetic diversity across large genomic regions

**Figure 5:**
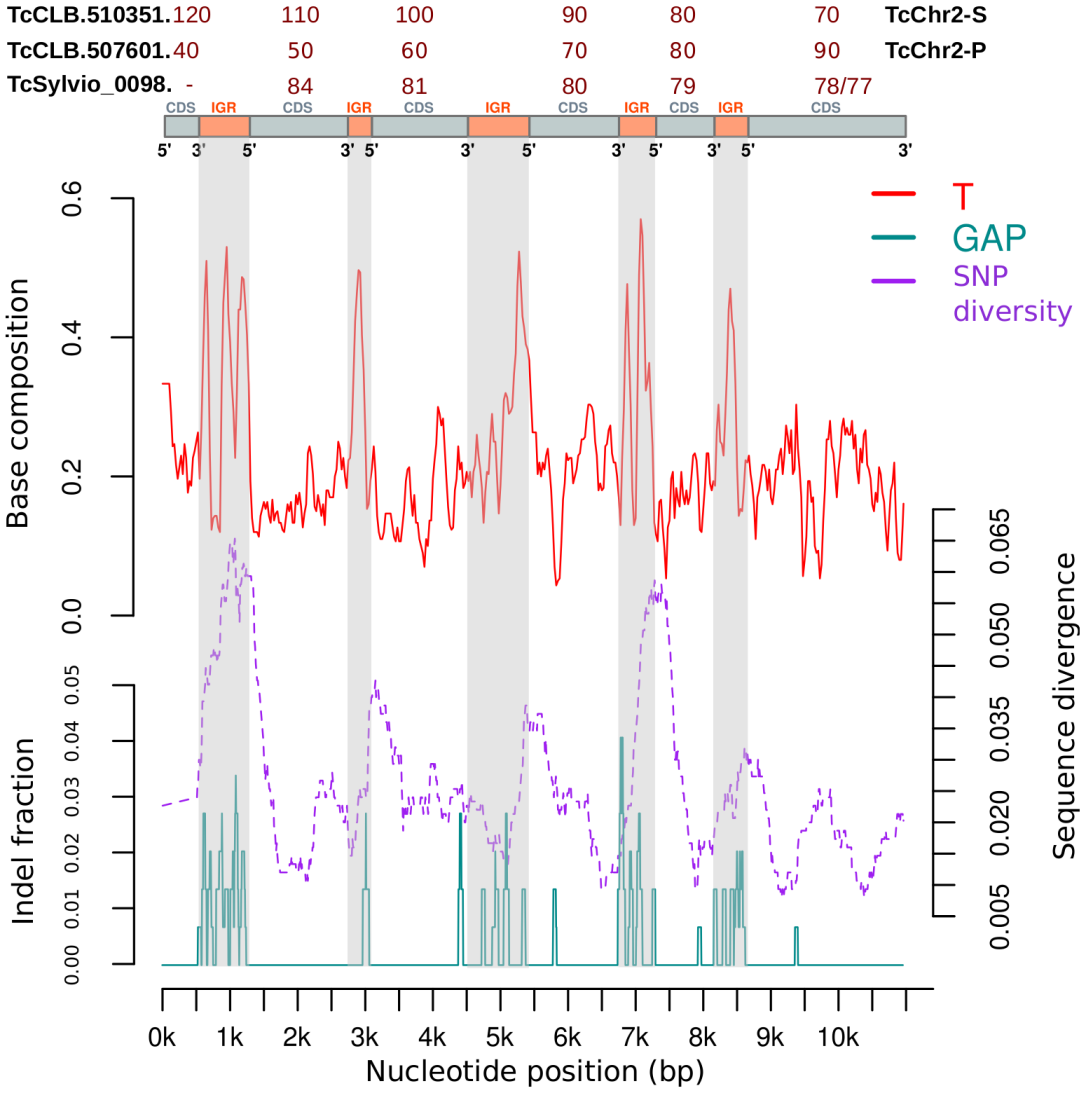
Distribution of indels, sequence composition and nucleotide diversity in a long genomic region. The plot shows values of thymidine composition, sequence divergence (SNPs) and indels, in a sliding window of 500 bp that was moved in 10 bp intervals. The region corresponds to TcChr2-S (43230–54007), TcChr2-P (46794–57559), and sylviocontig_9 (11357–22135). Annotated coding sequences in these region are TcCLB.507601.40–90 (TcChr2-P), TcCLB.510351.70–120 (TcChr2-S), TCSYLVIO_009877–9880 (GenBank accession ADWP02023504), and TCSYLVIO_009884 (GenBank accession ADWP02023505).

To visualize the dynamics of constraints on genetic diversity along large genomic regions, we next dissected the diversity into 3 main components (compositional bias, sequence divergence (SNPs, fixed changes), and indels), and observed their fluctuations over relatively large spans. For this analysis we selected a couple of genomic regions that had blocks of IGRs and CDSs that meet the criteria of Figure 1 of common evolutionary ancestry in the three haplotypes. The analysis shows fluctuations in the 3 components analyzed (see Figures 5, and S3), which follow the expected pattern based on CDS vs IGR comparison. However other trends are also apparent. Although the fluctuation of thymidine composition is large when comparing CDS vs IGR regions, it does not change significantly across IGR regions.

In contrast, indels and sequence divergence (i.e. SNPs) show more variability across IGRs, with some IGRs accumulating more changes than others. This analysis also showed that the measured components (SNPs, %T and %indels) peak abruptly within IGRs. However, when averaged across all IGRs they do not show a particular bias towards the 5′ end of the downstream CDS, the 3′ end of the upstream CDS, or the middle of the IGR (see Figures 5 and S3) (Kruskal-Wallis test 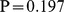, *a posteriori* test 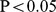).

### Diversification of allelic intergenic regions

**Figure 6:**
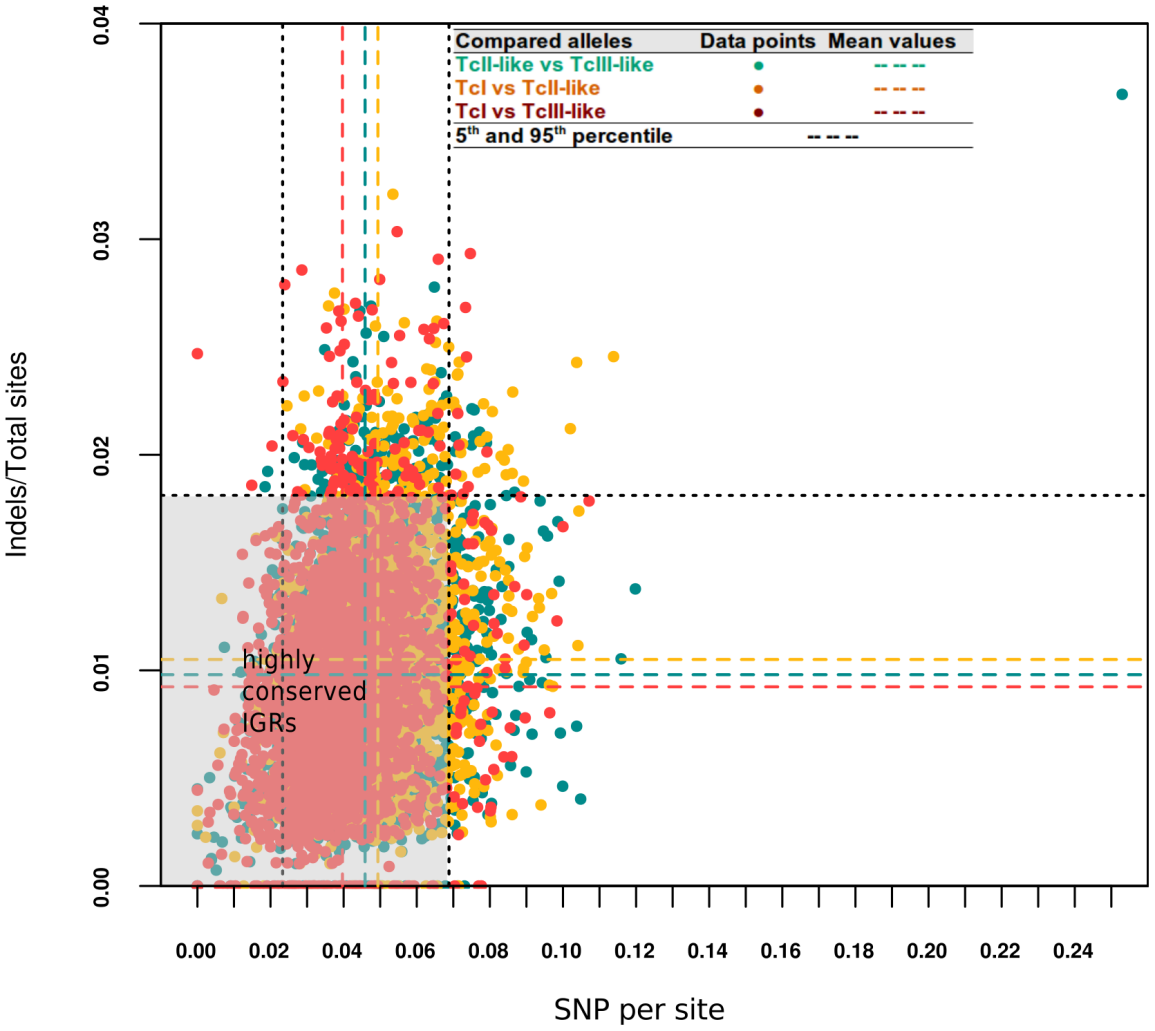
Plot of Indels vs SNPs in paired orthologous IGRs. The plot shows how each IGR region maps in space according to its nucleotide diversity and indel ratios. Each dot represents a pair of IGR regions. Colors indicate the different comparisons across haplotypes/genomes. Dotted lines represent the mean 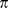 and indels/site values. In black dotted lines we also show the 5th and 95th percentile for each distribution.

Despite the strict requirements for allelic and orthologous genomic regions in our analysis (see Figure 1), we were able to identify significant diversity in these IGRs. The plot in Figure 6 shows the genetic diversity, decomposed in i) number of indels, measured as the ocurrence of indel events per total available sites, and ii) sequence divergence (SNPs), which essentially measures the proportion of base changes. Average values show, as expected, a higher accumulation of changes in IGR regions, with 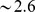 fold higher sequence divergence (SNPs per site, 0.051 vs 0.0196 in IGR vs CDS regions, respectively) and 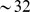 fold higher number of indels (0.0096 vs 0.0003 in IGR vs CDS regions, respectively).

As can be observed in Figure 6, within the shared 1,719 IGRs there is a core of highly conserved IGRs showing medium to low values of indels and SNPs per site. We defined them as placed within the 5th and 95th percentiles (see shaded rectangle in Figure 6) of both distributions (number of indels total sites, and sequence divergence). These IGRs constitute 90.6% of all IGRs. Outside this core, we found pairs of orthologous IGRs which are deviating either through accumulation of indels or SNPs, but rarely both. We only detected 0.7% of IGRs above the 95th percentile of both distributions (indels and SNPs per site). Whereas 4.3% of the IGRs display a higher proportion of either indels or SNPs per site.

**Figure 7:**
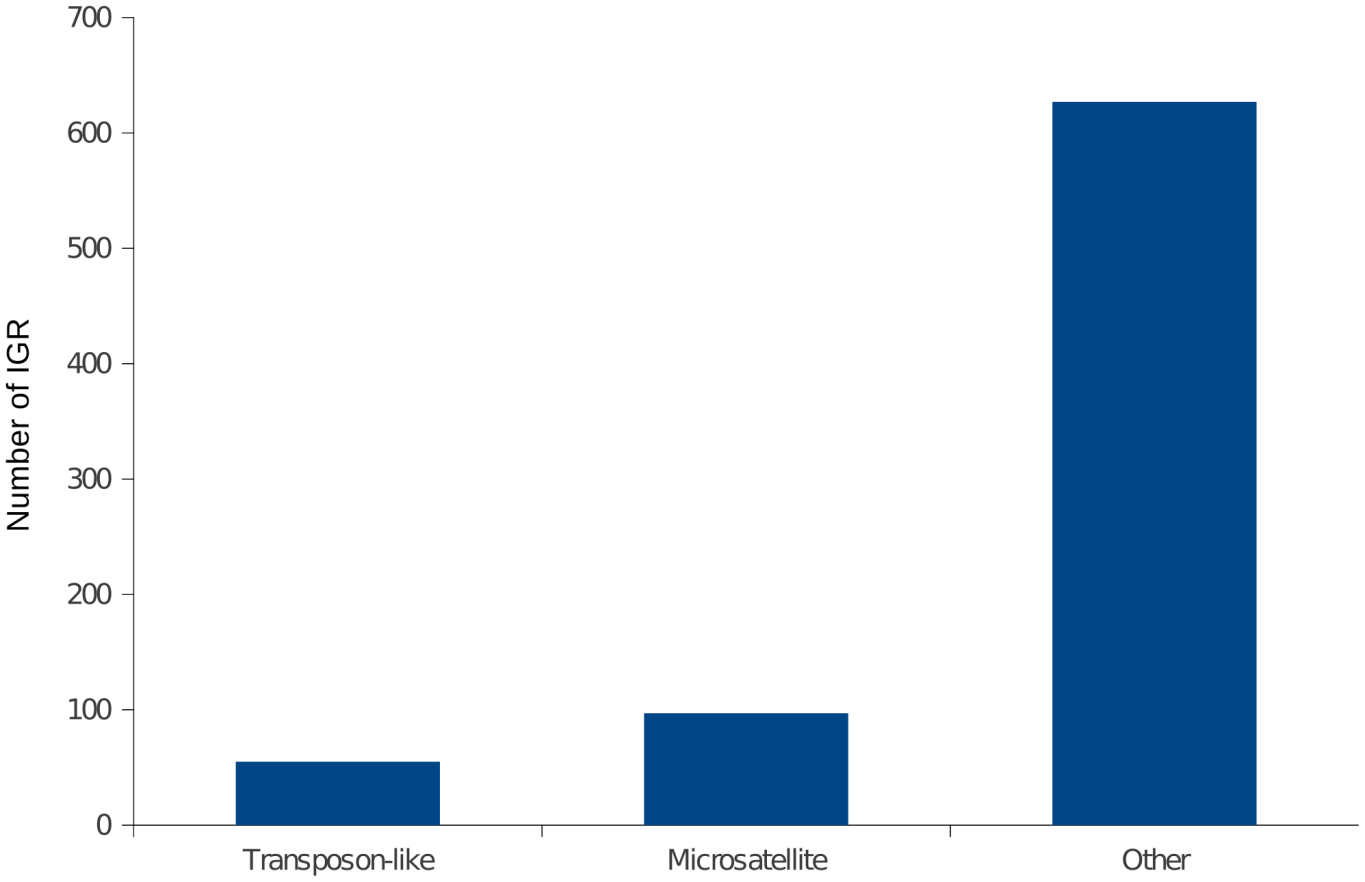
Contribution of repetitive elements to the observed length differences between IGRs. Repetitive elements detected by RepeatMasker (see Methods) were grouped into two broad categories: Transposon-like repetitive elements, and microsatellite-like repeats. The figure shows the contribution of these two classes of repetitive elements to the overall size of IGRs. The plot shows data for IGRs where the size difference between alleles is 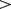
50 bp.

Indels are usually caused by polymerase slippage due to mispairing of a template strand during DNA replication and/or repair. Because IGRs accumulate more and longer homopolymer tracts (see Figure 4B) and/or are enriched in low-complexity nucleotide tracts, which are known to be prone to polymerase slippage [79]. However there are other mechanisms that could generate relatively large indels between orthologous IGRs, such as the insertion of a large transposon-like element, the expansion of microsatellite-like repeats, or large deletions. We next analyzed in more detail the types of indel accumulation, that contribute to the diversity between IGRs. To assess this, we searched a database of repetitive elements (RepBase, see Methods), and also looked for microsatellite-like repeats within IGRs. As can be observed (see Figure 7), most of the cases cannot be explained by these processes and are probably caused by other types of indel mutations. Within a set of 721 IGR regions (those with a size difference 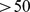 bp between alleles), only in 55 IGRs the main cause of length difference was the insertion of a transposon-like repetitive element (SIRE/Viper/LINE1). And only in 14 of these the transposon-like element was essentially complete (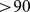
% of the element was contained within the IGR). Similarly, only in 97 cases the expansion/contraction of microsatellite-like repeats could be invoked as the cause of the length difference between IGRs.

One interesting case is a putative protein kinase (TcCLB.510565.70) that has a complete LTR/VIPER element in the IGR of the TcII-like haplotype, which is absent from the TcIII-like haplotype. Further inspection of other genomic data reveals that this element is apparently missing from TcI lineages (neither the SylvioX10, nor the JRcl4 IGR have this element), but present in the TcII lineage (Esmeraldo cl3). This suggests that the insertion pre-dated the hybridization event leading to the current extant TcVI lineage. A complete list of these sort of insertions is provided in Table S4.

We also observed large size differences that could not be attributed to transposons. The IGR downstream of an acidocalcisomal exopolyphosphatase (TcCLB.511577.110) is large (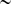 2.2 Kbp) in the TcI (Sylvio X10) and TcIII-like haplotypes, but shorter (only 600 bp) in the TcII-like and TcII (Esmeraldo cl3) haplotypes (See Figure S4). Since there are no repetitive elements within this IGR, it is likely that a single large deletion event took place, while preserving the 3′ UTR of the exopolyphosphatase and a short stretch of the 5′ UTR of the downstream hypothetical protein.

Finally, an example of a large size difference that could be driven by expansion/contraction of microsatellite-like repeats is the IGR downstream of a putative sphingosine kinase (TcCLB.508211.30/TcCLB.507515.120). Figure S5 shows an alignment of this IGR, which displays a number of (CATA/TATG)n, (TA)n, (TAA/TTA)n repeats, along with other imperfect repeats, and homopolymers (mostly (T)n). In this case the contraction/expansion of these repeats could have worked together with an ancestral indel event in shaping the current form of this IGR in the TcII-like lineage.

It is tempting to speculate that in any of these cases the observed variation between IGRs could differentially affect the regulation of upstream and/or downstream transcripts in one or more lineages. This is because in trypanosomes, the co-transcriptional processing of polycistronic transcripts, display a tight coupling of polyadenylation of the upstream CDS and *trans-*splicing of the downstream CDS. A large insertion or deletion could therefore alter this coupling or effectively decouple these processes, potentially altering the expression pattern of one or both flanking CDSs. Unfortunately there are currently no genome-wide studies of gene expression for different lineages or under different conditions that could be compared.

### Intergenic sequences shared by unrelated loci

While analyzing intergenic regions we noticed a number of such regions that produced a higher than expected number of BLASTN hits when doing an all vs all comparison. These additional BLAST hits were produced by the presence of blocks of significant sequence similarity between non-allelic regions. Based on this BLAST analysis, we were able to identify 21 elements of defined sequence, which were shared by 2–4 different IGRs. These sequence elements are embedded within larger IGR regions, but the sequence similarity shared by the non-allelic IGRs in each group is limited to a portion of the IGR, and there is no other sequence similarity between the flanking coding sequences. Information about these IGRs is provided as supplementary information (Dataset S1), including the sequences themselves, as well as multiple sequence alignments and a schematic figure showing the relative location of each element. The mean length of these blocks was 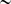 427 bp with an average identity of 90.71%. The relative location of these sequence elements within each group of IGRs varies. In some cases the shared element is at the 5′ end of the IGRs (e.g. case #12), or at a similar short distance to the 5′ end (case #13). In these cases, one could assume that these shared regions could be part of the corresponding 3′-UTRs of the upstream genes. In other cases (case #10), the shared region appears closer to the 3′ end of the IGR (e.g. could form part of the 5′-UTR of the downstream gene). Whereas in other cases the shared element is located either closer to the 3′ or 5′ end in different IGRs.

To further address the functional relevance of these conserved blocks of sequence we first considered the possibility that they were non-coding RNAs, or small ORFs (sORFs). However, a search of the Rfam database revealed only one case assignable to ncRNA in these IGRs (IGR 1869, Case 4, Dataset S1). This IGR contains a Gln tRNA but in a region that is not part of the block of similarity shared with other IGRs. We also failed to reveal similarity between these conserved stretches and recently described ncRNAs in *T. cruzi* [80]. And a BLASTP search against a list of validated sORFs from yeast [81], [82] revealed only partial matches of dubious significance. Finally, to see if these elements were transcribed in *T. cruzi* we took advantage of the recent availability of RNAseq (transcriptomic) data from the TcAdriana strain (TcI, obtained by 454 sequencing of an epimastigote cDNA library, Westeergard G and Vazquez MP, unpublished [83]). After mapping reads to these IGRs, we were able to confirm that nearly all of them (the exception is case #19) showed evidence of expression at different levels. A careful analysis of the mapped reads (looking for specific SNPs and indels) revealed that all the IGRs in these groups were transcribed. We further examined these reads to look for evidence of *trans-*splicing, to see if these could be independent, short transcripts, or if, alternatively, they were part of the UTRs of the upstream genes. However, we did not find any matches to the *T. cruzi* miniexon (spliced leader) sequence in these mapped reads. Therefore, the most plausible explanation is that the transcript that include these shared IGR blocks originate from the 3′UTR of the upstream CDSs (or are part of dicistronic transcripts [84]).

### Probing the diversity of intergenic regions in an expanded panel of strains

**Figure 8:**
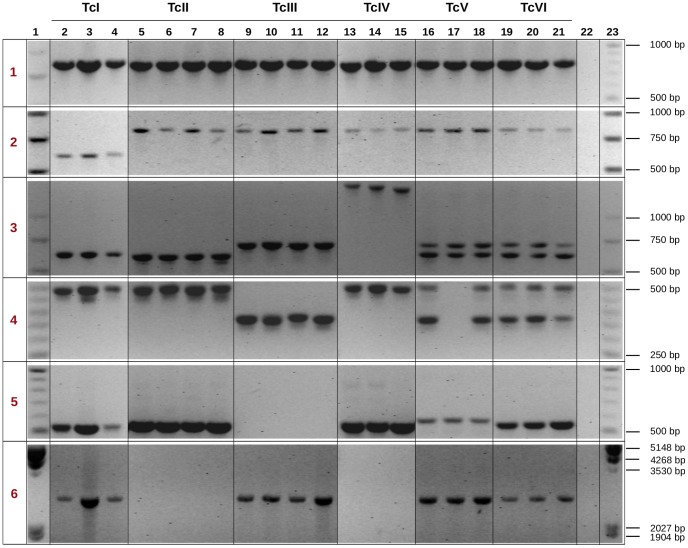
PCR amplification of selected IGR regions in different strains of *T. cruzi*. Selected genomic regions were amplified to validate length and sequence polymorphisms, and resolved in a 2% TBE-agarose gel. Lanes in the gels correspond to: molecular size markers (lanes 1, 23), DNA from *T. cruzi* strains (lanes 2–21), and negative control (lane 22). Strains used (and the corresponding lanes) are: 92122102R (2); Dog Theis (3); CanIII (4); TU18 (5); Mas1 cl1 (6); IVV cl4 (7); Y9 IIB (8); X109-02 (9); M5631 cl5 (10); M6241 cl6 (11); LL051 (12); Mn cl2 (13); Sc43 cl9 (14); TEH53 (15); Tula cl2 (16); CL Brener (17); P63 cl1 (18); Sylvio X10/1 (19); Palv2 (20); Dm28c (21). Numbers in the leftmost column (1–6) correspond to the six selected cases mentioned in the text. All samples were analyzed in the same electrophoresis run, however for clarity purposes, groups of lanes were digitally re-ordered.

To explore the diversity of IGR lengths, and to validate the observed IGR sizes, we selected a number of interesting cases and performed PCR experiments followed by gel electrophoresis. This analysis was done using a complete panel of strains representative of all major lineages/DTUs. As can be seen in Figure 8, except for the lack of amplification of long PCR products, the amplification produced the expected results in all cases. The IGR in case 1, which was selected based on its size conservation in analyzed genomes, also showed no apparent size differences in other examined strains. In cases 2–6, there was a unique IGR length associated with one DTU/lineage in our bioinformatics analysis (see Table S2). In case 2, the unique IGR length observed in Sylvio X10 (TcI) was validated in 2 other strains from the same DTU. In this case, the IGR length observed for the TcII-like and TcIII-like alleles in our bioinformatics analysis is the same observed in all other strains/DTUs analyzed. In case 3, we validated the predicted IGR size, and discovered that all strains analyzed from the TcIV DTU carried a longer IGR region. In case 4 strains from the TcI, TcII and TcIV DTUs showed a similar length that was different from TcIII, whereas the hybrid lineages showed two bands with sizes that agree with the proposed ancestral hybridization of TcII and TcIII parental DTUs. Finally, the fifth and sixth cases correspond to two cases (IGRs 333 and 2320) both of which were already mentioned in the text. IGR 333 corresponds to a small IGR in all lineages, except in the TcIII-like haplotype of CL Brener, where there is a 4.4 Kbp insertion of an LTR/Viper element. Failure of amplification in TcIII strains is expected given the large size of the PCR product and the thermal cycling protocol employed. Similarly, IGR 2320 displayed a large deletion in TcI and in the TcIII-like haplotype (see Fig S5), which was validated in other TcI and TcIII strains. Failure to amplify this IGR in TcII strains is consistent with the prediction of a large IGR size. In this validation study the failure to also amplify this IGR from TcIV strains suggests that this IGR is also large in these strains, or that a chromosomal rearrangement has occurred, which rewired the corresponding upstream and downstream coding sequences.

These results show that the IGR sizes observed *in silico* could be validated experimentally, and in many independent strains from each DTU. Overall we have observed a high degree of IGR size conservation within DTUs, at least in these selected cases. It is important to observe that in all cases the TcII and TcIII strains produced amplification bands of the expected size based on the analysis of the CL Brener (TcVI) genome. This further validates the idea that by analyzing an hybrid genome we can get an insight into the genetic diversity of IGR (this work) and CDS regions [21] of *T. cruzi*. Finally, the expanded strain panel used allowed us to also inspect the size conservation in other DTUs (TcIII, TcIV), for which there is currently no genome sequence available.

### Conclusions

The genome of *T. cruzi* is highly repetitive [41], [42], [85] as well as complex, due to the chromosomal rearrangements observed in different strains, and the hybrid nature of strains from lineages TcV and TcVI [86], [87]. However, both recent work focused on coding sequences [20], [21], as well as this work focused on noncoding IGRs show that in spite of this complexity there is a highly conserved core of genes that have maintained their IGRs, with a significant fraction of them under apparent purifying selection, as suggested by the paucity of accumulation of mutations and/or indels. At least in the subset of genes analyzed, it is clear that in those cases where IGRs differ between the analyzed genomes, indels outnumber other types of mutations (SNPs/base changes due to polymerase read errors or DNA repair and insertions of transposable elements). Although mostly focused on single-copy genes/IGRs, this work represents the first global analysis of genetic diversity of *T. cruzi* non-coding DNA.

## Supporting Information

Figure S1 **Size correlation of orthologous coding sequences.** The plot shows the pairwise comparisons of the length of CDS regions between the three analyzed haplotypes/genomes: A) TcI vs TcII-like, B) TcII-like vs TcIII-like, C) TcI vs TcIII-like. The colored dotted line in each plot marks the mean value of each distribution, while the black dotted lines mark the 5th and 95th percentiles, respectively. Plot axes correspond to length (size) of the coding sequence in base pairs, for each haplotype/genome.
(TIF)

Figure S2
**Comparison of nucleotide diversity values (** 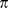 **) between CDS and IGR regions.** The figure shows all possible pairwise comparisons of the data: TcI vs TcII-like, TcII-like vs TcIII-like, and TcI vs TcIII-like.(TIF)

Figure S3
**Distribution of indels, sequence composition and nucleotide diversity in a long genomic region.** The plot shows values of thymidine composition, nucleotide diversity (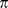) and indels, in a sliding window of 500 bp that was moved in 10 bp intervals. The region corresponds to TcChr35-S (850268–859701), TcChr35-P (850262–859611), and sylviocontig_39 (556–9894). Annotated coding sequences in these region are TcCLB.511717.140–180 (TcChr35-P), TcCLB.506223.50–90 (TcChr35-S) and TCSYLVIO_004150–4154 (GenBank accession ADWP02013978).(TIF)

Figure S4 **Multiple sequence alignment of the intergenic region downstream of an acidocalcisomal exopolyphosphatase.** Bases are colored by class (purine/pyrimidine).
(TIF)

Figure S5 **Multiple sequence alignment of the intergenic region downstream of putative sphingosine kinase.** Bases are colored by class (purine/pyrimidine). Blue lines mark the locations of perfect microsatellite-like repeats.
(TIF)

Table S1 **Summary of data analyzed in this work.** The file outlines the bulk of data that was analysed both for coding and non-coding genomic regions in all three haplotypes.
(XLS)

Table S2 **List of oligonucleotide primers and expected IGR size of genomic regions that were selected for experimental validation.** The table lists the flanking coding sequences (CDS) for each IGR region, and the expected PCR product size for each set of oligonucleotide primers. Case numbers match those in Figure 8.
(XLS)

Table S3 **List of intergenic regions analyzed in this work.** In the spreadsheet file we provide the complete list of intergenic regions used in this work, together with the internal identifier of each IGR, the locus identifiers and/or coordinates of the flanking coding sequences in each genome, the length of the IGR in each genome, and a consensus summary of repetitive elements identified in each IGR.
(XLS)

Table S4 **Presence of repetitive DNA elements in intergenic regions.** The spreadsheet contains a list of simple repeats and repetitive elements found in IGRs. These are the result of searching RepBase[52] with RepeatMasker[53] (see Methods). The table lists the coordinates where the repetitive element is mapped and the coverage (both with respect to the IGR and the repetitive element).
(XLS)

Dataset S1
**Intergenic regions from unrelated loci that share blocks of significant sequence similarity.** The file contains i) a spreadsheet summarizing listing the non-allelic, non-homologous IGR regions that share significant blocks of sequence similarity; ii) multiple sequence alignments where these unrelated IGRs were aligned to highlight the portion of the IGR that is shared; and iii) a figure in PDF format that shows the alignment in context. The alignments are provided in CLUSTAL format (.clw) and as colored renderings in PNG format, as produced by Jalview (purine/pyrimidine color scheme, only applied to regions of the alignment with 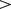
90% identity). The IGR IDs listed in each alignment correspond to those in Table S3.(ZIP)
