# Supplementary figures and images for: A Genome-Wide Analysis of Genetic Diversity in Trypanosoma cruzi Intergenic Regions
Source: PLoS Negl Trop Dis. 2014 May 1;8(5):e2839. doi: 10.1371/journal.pntd.0002839 (PMC4006747; doi:10.1371/journal.pntd.0002839)

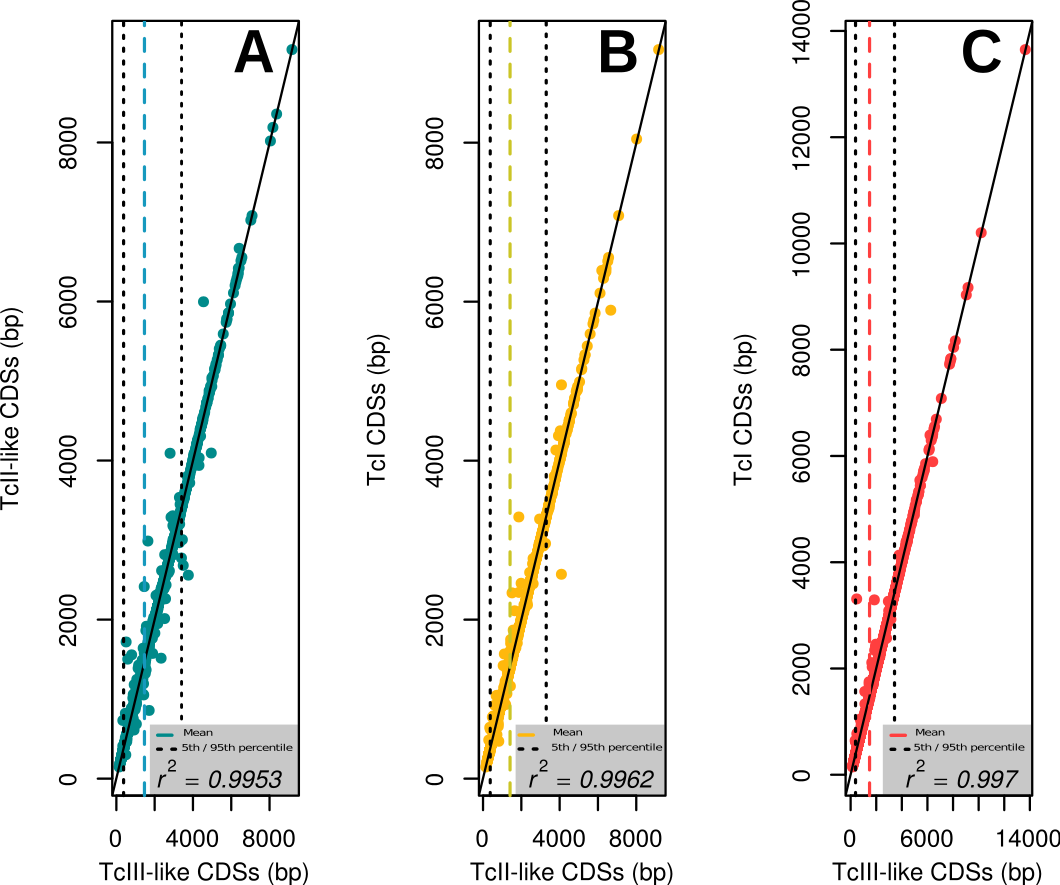

Supplement: Figure S1 — Size correlation of orthologous coding sequences. The plot shows the pairwise comparisons of the length of CDS regions between the three analyzed haplotypes/genomes: A) TcI vs TcII-like, B) TcII-like vs TcIII-like, C) TcI vs TcIII-like. The colored dotted line in each plot marks the mean value of each distribution, while the black dotted lines mark the 5th and 95th percentiles, respectively. Plot axes correspond to length (size) of the coding sequence in base pairs, for each haplotype/genome. (TIF) [file pntd.0002839.s001.tif]

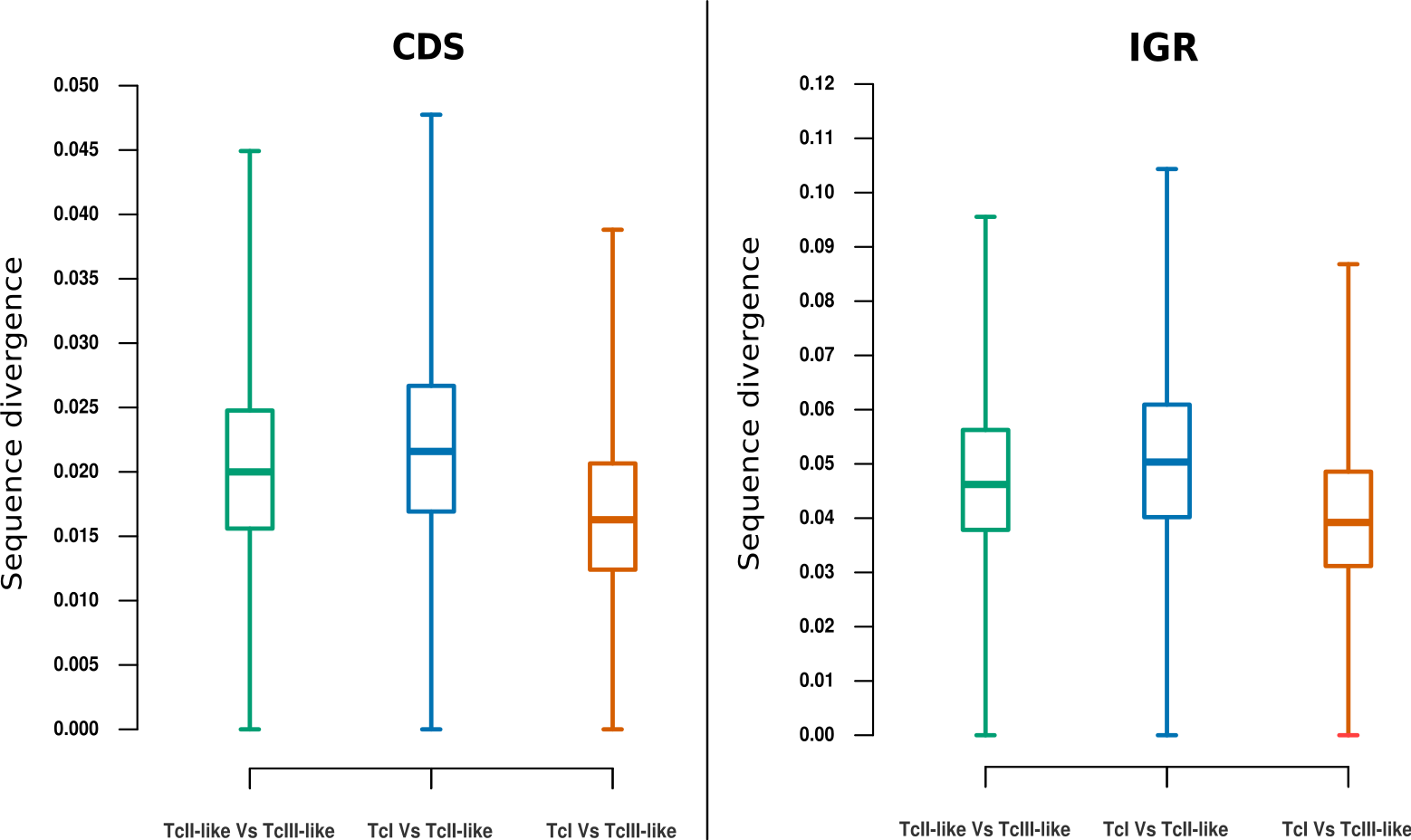

Supplement: Figure S2 — Comparison of nucleotide diversity values ( ) between CDS and IGR regions. The figure shows all possible pairwise comparisons of the data: TcI vs TcII-like, TcII-like vs TcIII-like, and TcI vs TcIII-like. (TIF) [file pntd.0002839.s002.tif]

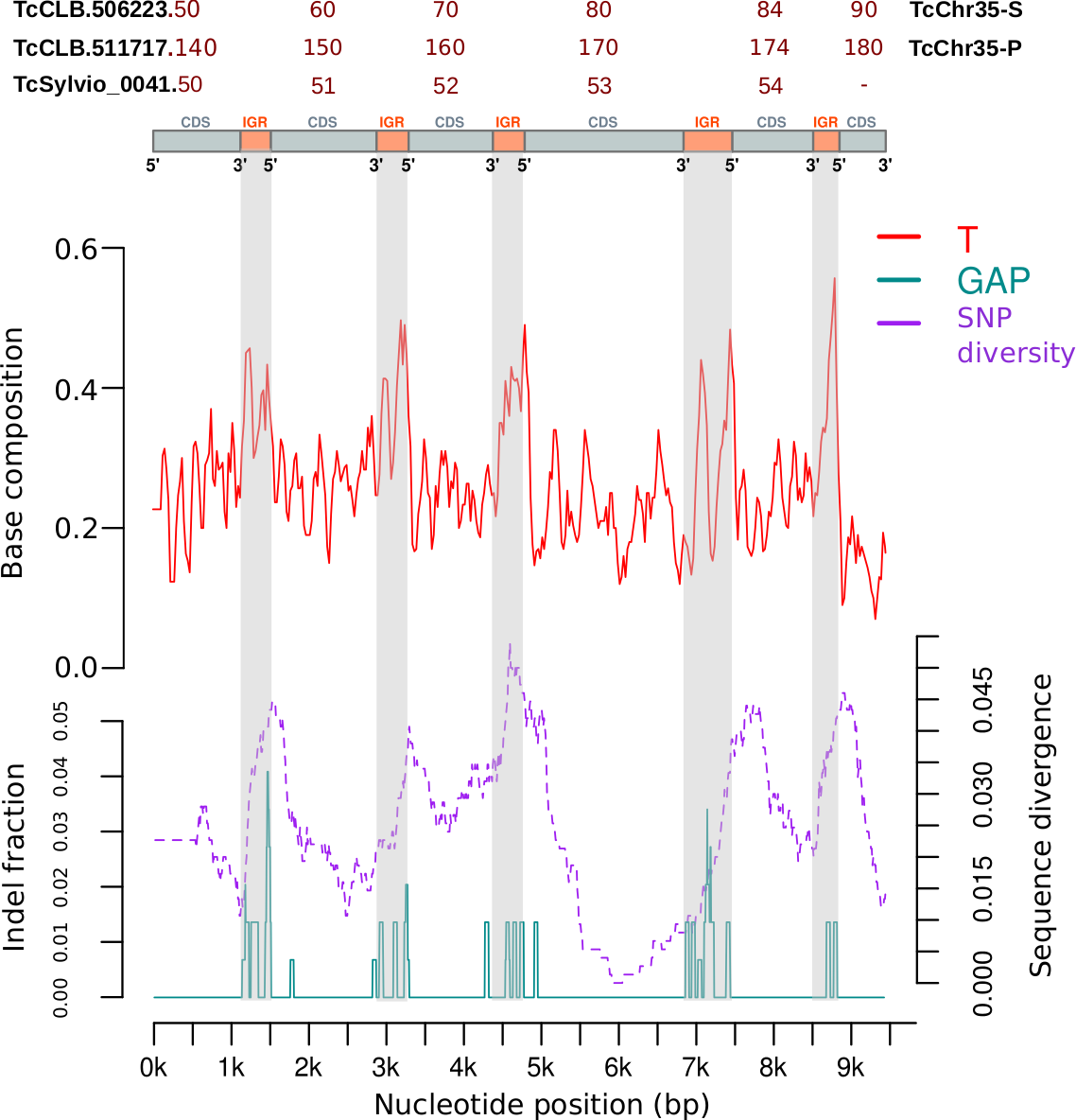

Supplement: Figure S3 — Distribution of indels, sequence composition and nucleotide diversity in a long genomic region. The plot shows values of thymidine composition, nucleotide diversity () and indels, in a sliding window of 500 bp that was moved in 10 bp intervals. The region corresponds to TcChr35-S (850268–859701), TcChr35-P (850262–859611), and sylviocontig_39 (556–9894). Annotated coding sequences in these region are TcCLB.511717.140–180 (TcChr35-P), TcCLB.506223.50–90 (TcChr35-S) and TCSYLVIO_004150–4154 (GenBank accession ADWP02013978). (TIF) [file pntd.0002839.s003.tif]

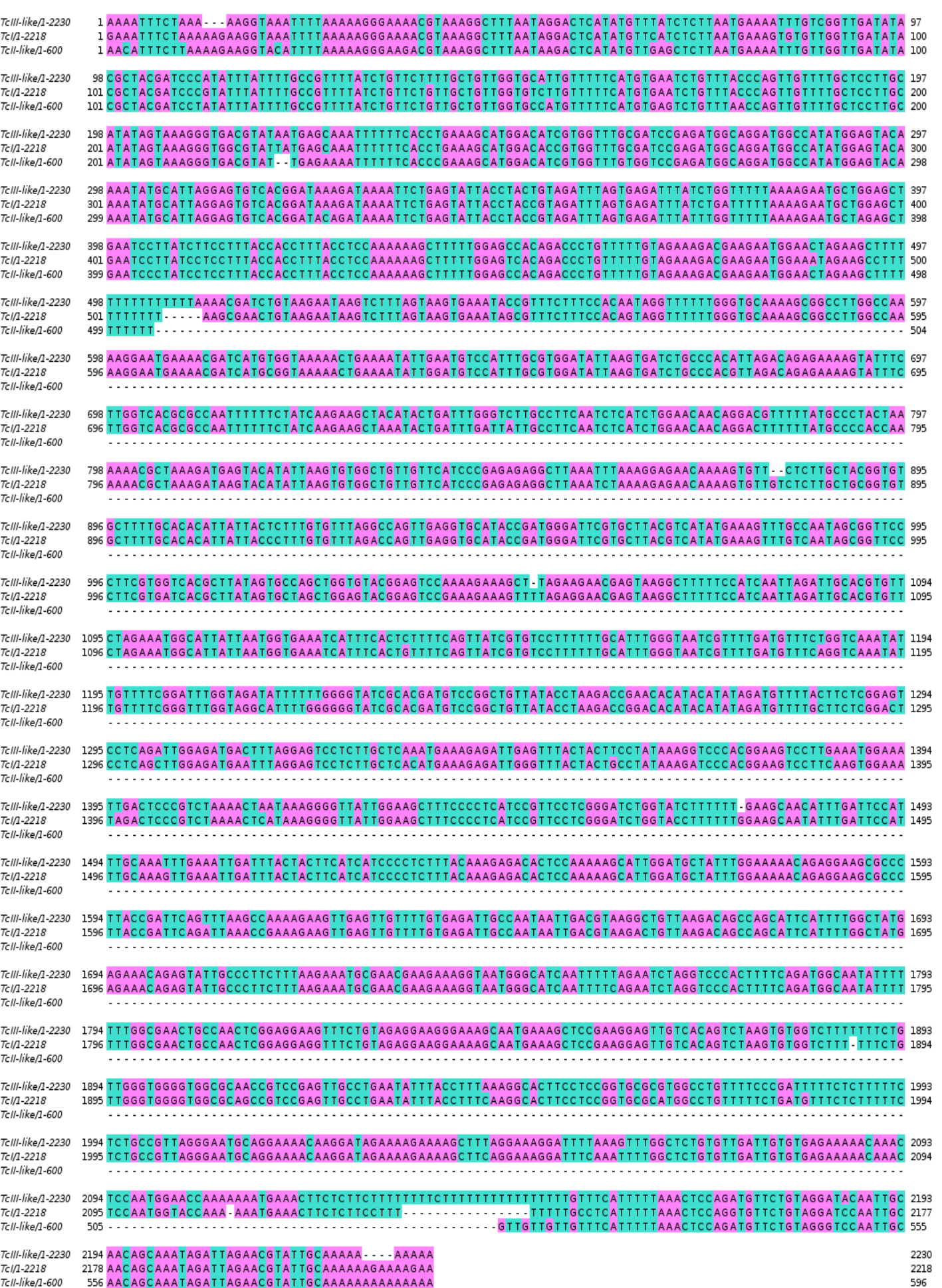

Supplement: Figure S4 — Multiple sequence alignment of the intergenic region downstream of an acidocalcisomal exopolyphosphatase. Bases are colored by class (purine/pyrimidine). (TIF) [file pntd.0002839.s004.tif]

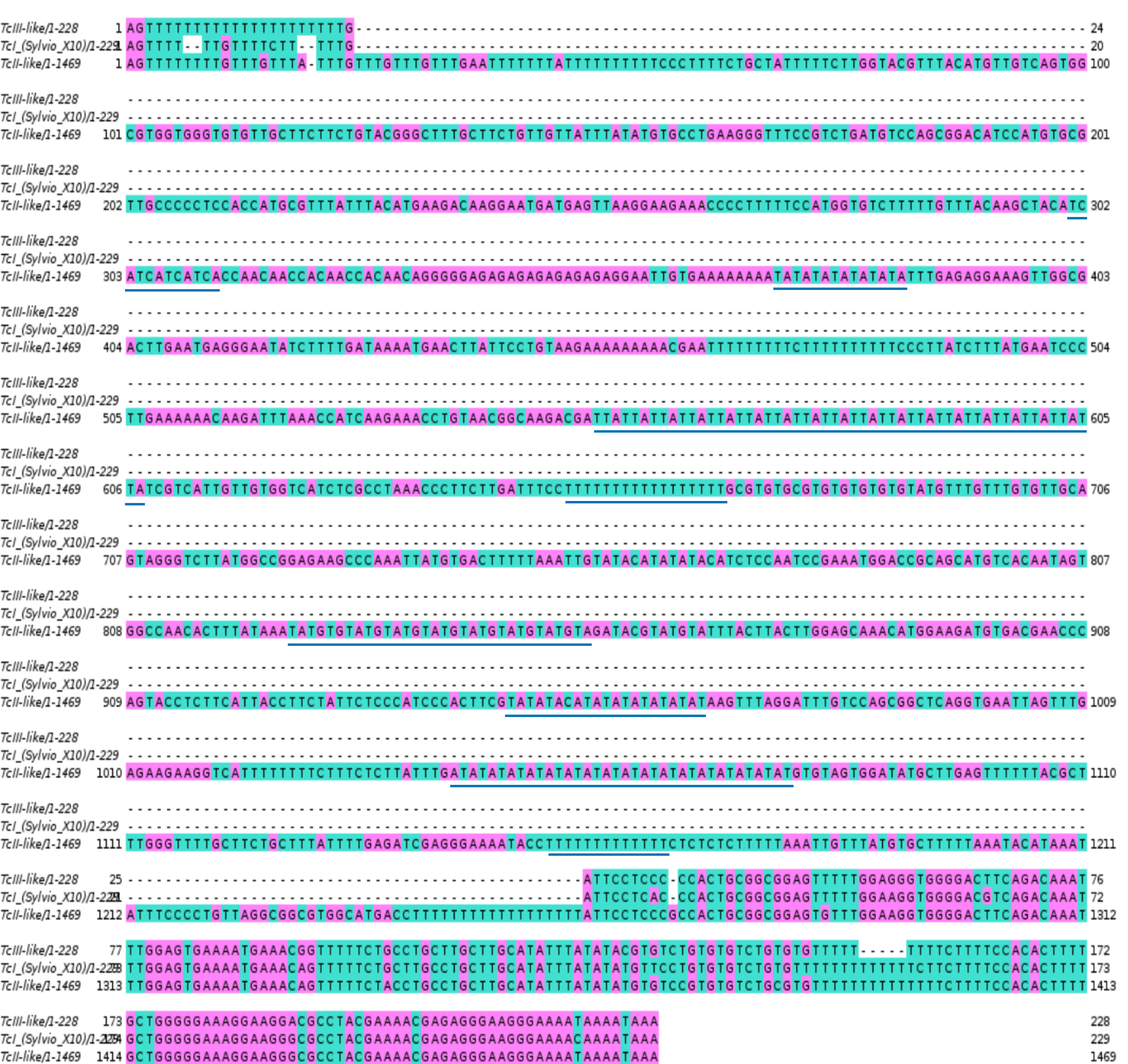

Supplement: Figure S5 — Multiple sequence alignment of the intergenic region downstream of putative sphingosine kinase. Bases are colored by class (purine/pyrimidine). Blue lines mark the locations of perfect microsatellite-like repeats. (TIF) [file pntd.0002839.s005.tif]

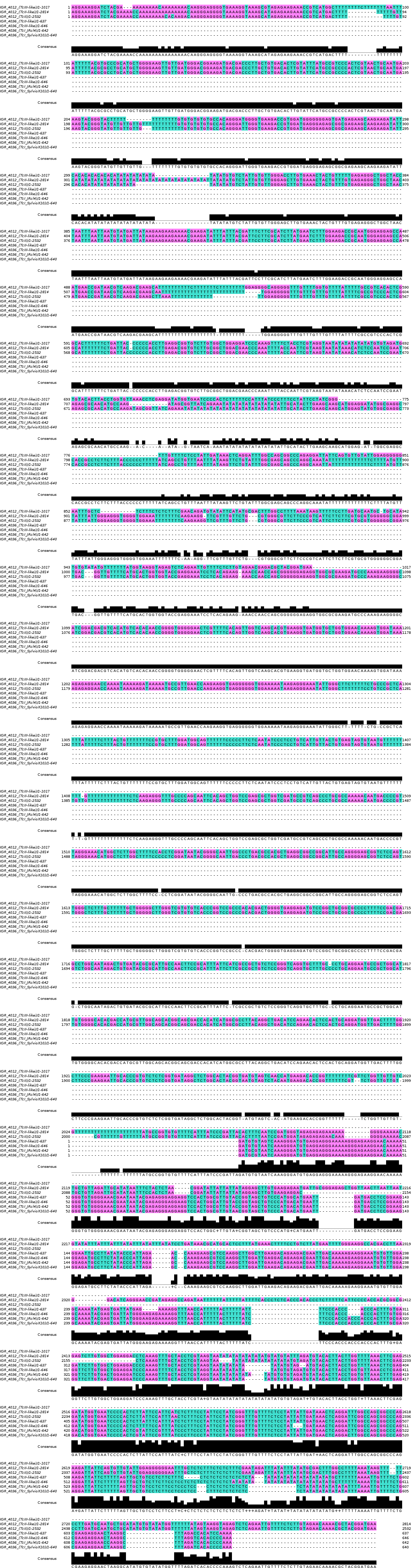

Supplement: Dataset S1 — Intergenic regions from unrelated loci that share blocks of significant sequence similarity. The file contains i) a spreadsheet summarizing listing the non-allelic, non-homologous IGR regions that share significant blocks of sequence similarity; ii) multiple sequence alignments where these unrelated IGRs were aligned to highlight the portion of the IGR that is shared; and iii) a figure in PDF format that shows the alignment in context. The alignments are provided in CLUSTAL format (.clw) and as colored renderings in PNG format, as produced by Jalview (purine/pyrimidine color scheme, only applied to regions of the alignment with 90% identity). The IGR IDs listed in each alignment correspond to those in Table S3. (ZIP) [file pntd.0002839.s010.zip › Dataset S1/alignment-pictures/entire-IGR/IGR_Group_14.png]

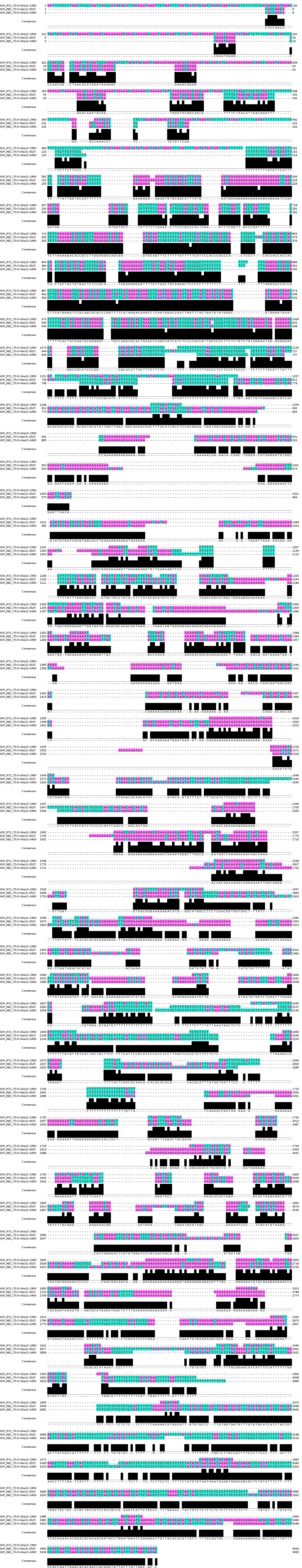

Supplement: Dataset S1 — Intergenic regions from unrelated loci that share blocks of significant sequence similarity. The file contains i) a spreadsheet summarizing listing the non-allelic, non-homologous IGR regions that share significant blocks of sequence similarity; ii) multiple sequence alignments where these unrelated IGRs were aligned to highlight the portion of the IGR that is shared; and iii) a figure in PDF format that shows the alignment in context. The alignments are provided in CLUSTAL format (.clw) and as colored renderings in PNG format, as produced by Jalview (purine/pyrimidine color scheme, only applied to regions of the alignment with 90% identity). The IGR IDs listed in each alignment correspond to those in Table S3. (ZIP) [file pntd.0002839.s010.zip › Dataset S1/alignment-pictures/entire-IGR/IGR_Group_7.png]

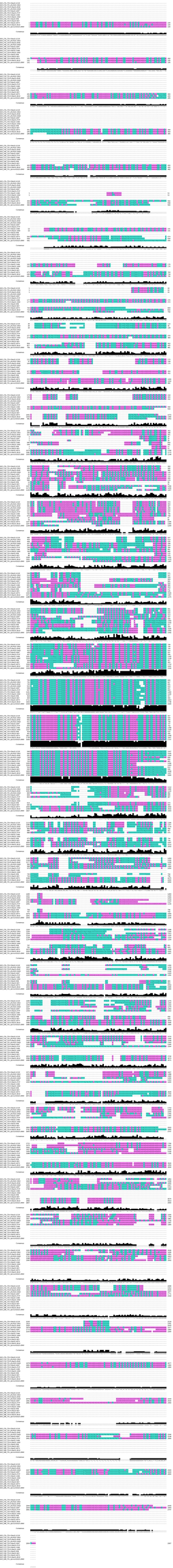

Supplement: Dataset S1 — Intergenic regions from unrelated loci that share blocks of significant sequence similarity. The file contains i) a spreadsheet summarizing listing the non-allelic, non-homologous IGR regions that share significant blocks of sequence similarity; ii) multiple sequence alignments where these unrelated IGRs were aligned to highlight the portion of the IGR that is shared; and iii) a figure in PDF format that shows the alignment in context. The alignments are provided in CLUSTAL format (.clw) and as colored renderings in PNG format, as produced by Jalview (purine/pyrimidine color scheme, only applied to regions of the alignment with 90% identity). The IGR IDs listed in each alignment correspond to those in Table S3. (ZIP) [file pntd.0002839.s010.zip › Dataset S1/alignment-pictures/entire-IGR/IGR_Group_6.png]

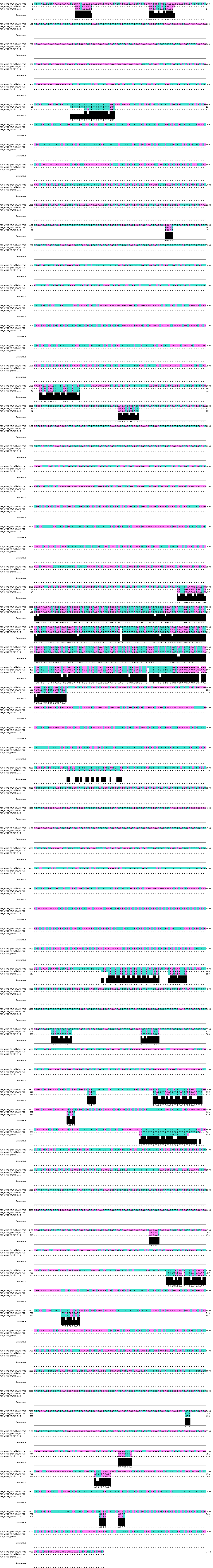

Supplement: Dataset S1 — Intergenic regions from unrelated loci that share blocks of significant sequence similarity. The file contains i) a spreadsheet summarizing listing the non-allelic, non-homologous IGR regions that share significant blocks of sequence similarity; ii) multiple sequence alignments where these unrelated IGRs were aligned to highlight the portion of the IGR that is shared; and iii) a figure in PDF format that shows the alignment in context. The alignments are provided in CLUSTAL format (.clw) and as colored renderings in PNG format, as produced by Jalview (purine/pyrimidine color scheme, only applied to regions of the alignment with 90% identity). The IGR IDs listed in each alignment correspond to those in Table S3. (ZIP) [file pntd.0002839.s010.zip › Dataset S1/alignment-pictures/entire-IGR/IGR_Group_17.png]

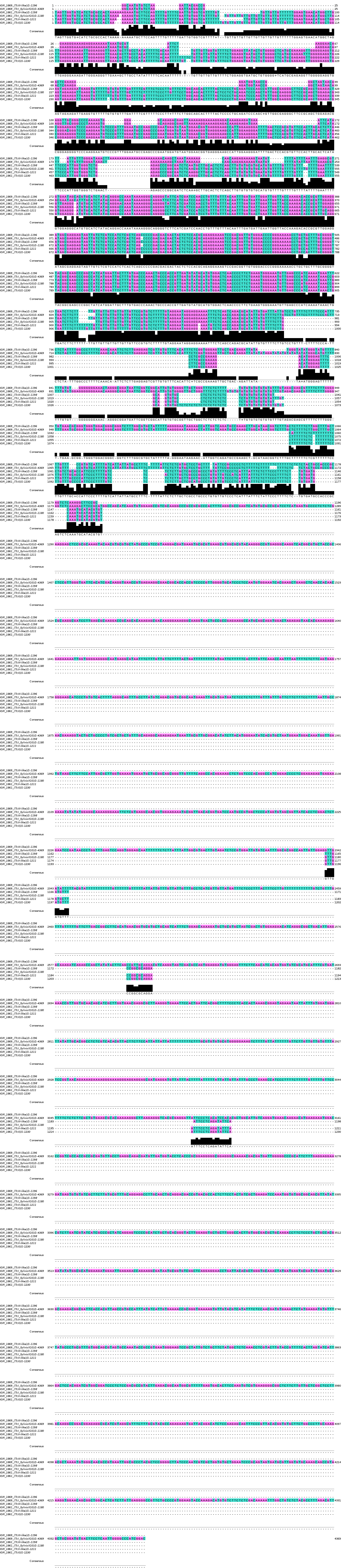

Supplement: Dataset S1 — Intergenic regions from unrelated loci that share blocks of significant sequence similarity. The file contains i) a spreadsheet summarizing listing the non-allelic, non-homologous IGR regions that share significant blocks of sequence similarity; ii) multiple sequence alignments where these unrelated IGRs were aligned to highlight the portion of the IGR that is shared; and iii) a figure in PDF format that shows the alignment in context. The alignments are provided in CLUSTAL format (.clw) and as colored renderings in PNG format, as produced by Jalview (purine/pyrimidine color scheme, only applied to regions of the alignment with 90% identity). The IGR IDs listed in each alignment correspond to those in Table S3. (ZIP) [file pntd.0002839.s010.zip › Dataset S1/alignment-pictures/entire-IGR/IGR_Group_4.png]

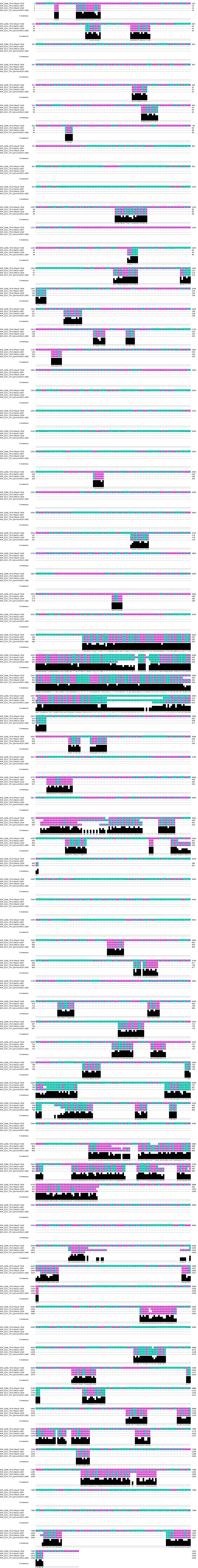

Supplement: Dataset S1 — Intergenic regions from unrelated loci that share blocks of significant sequence similarity. The file contains i) a spreadsheet summarizing listing the non-allelic, non-homologous IGR regions that share significant blocks of sequence similarity; ii) multiple sequence alignments where these unrelated IGRs were aligned to highlight the portion of the IGR that is shared; and iii) a figure in PDF format that shows the alignment in context. The alignments are provided in CLUSTAL format (.clw) and as colored renderings in PNG format, as produced by Jalview (purine/pyrimidine color scheme, only applied to regions of the alignment with 90% identity). The IGR IDs listed in each alignment correspond to those in Table S3. (ZIP) [file pntd.0002839.s010.zip › Dataset S1/alignment-pictures/entire-IGR/IGR_Group_9.png]

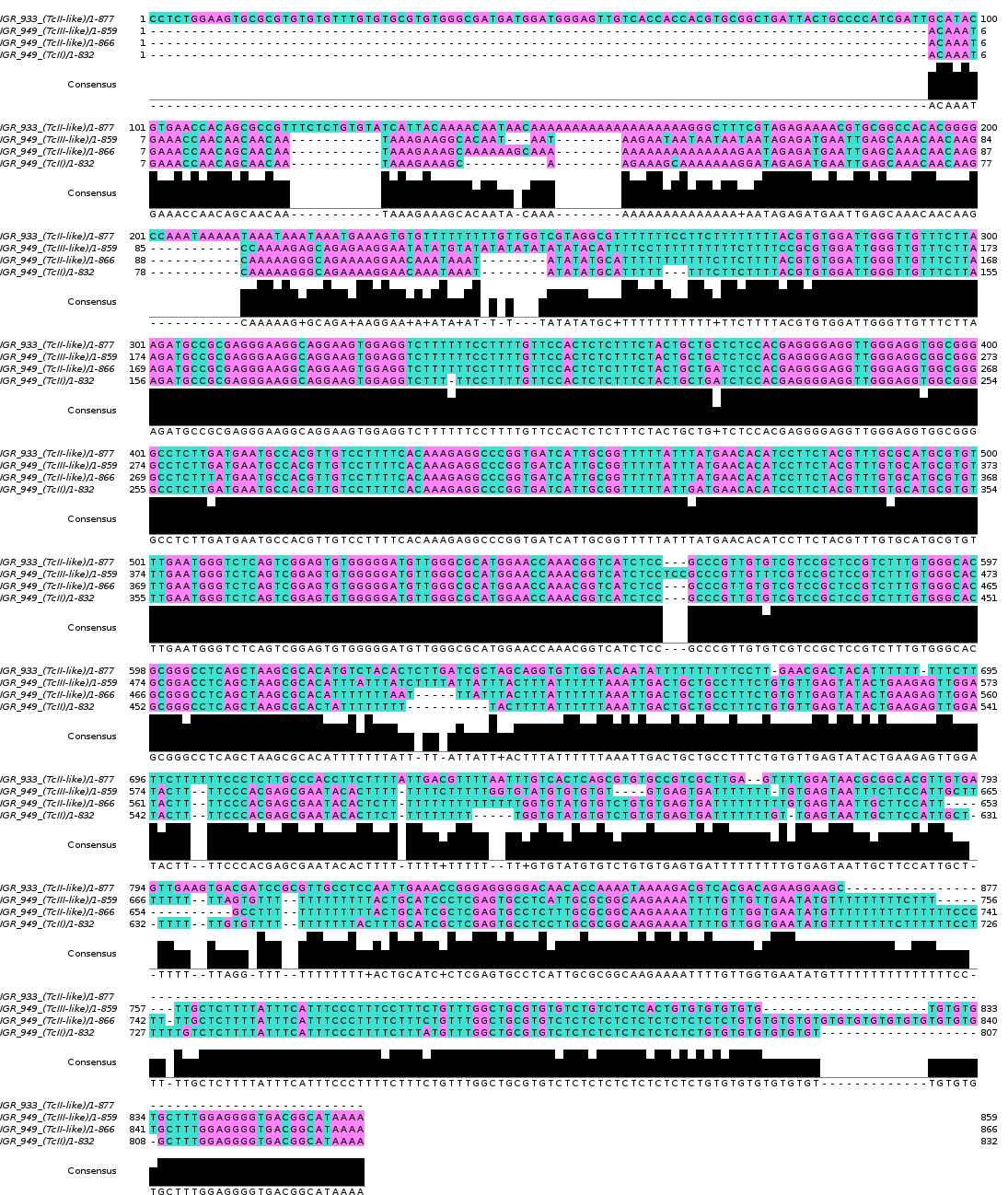

Supplement: Dataset S1 — Intergenic regions from unrelated loci that share blocks of significant sequence similarity. The file contains i) a spreadsheet summarizing listing the non-allelic, non-homologous IGR regions that share significant blocks of sequence similarity; ii) multiple sequence alignments where these unrelated IGRs were aligned to highlight the portion of the IGR that is shared; and iii) a figure in PDF format that shows the alignment in context. The alignments are provided in CLUSTAL format (.clw) and as colored renderings in PNG format, as produced by Jalview (purine/pyrimidine color scheme, only applied to regions of the alignment with 90% identity). The IGR IDs listed in each alignment correspond to those in Table S3. (ZIP) [file pntd.0002839.s010.zip › Dataset S1/alignment-pictures/entire-IGR/IGR_Group_8.png]

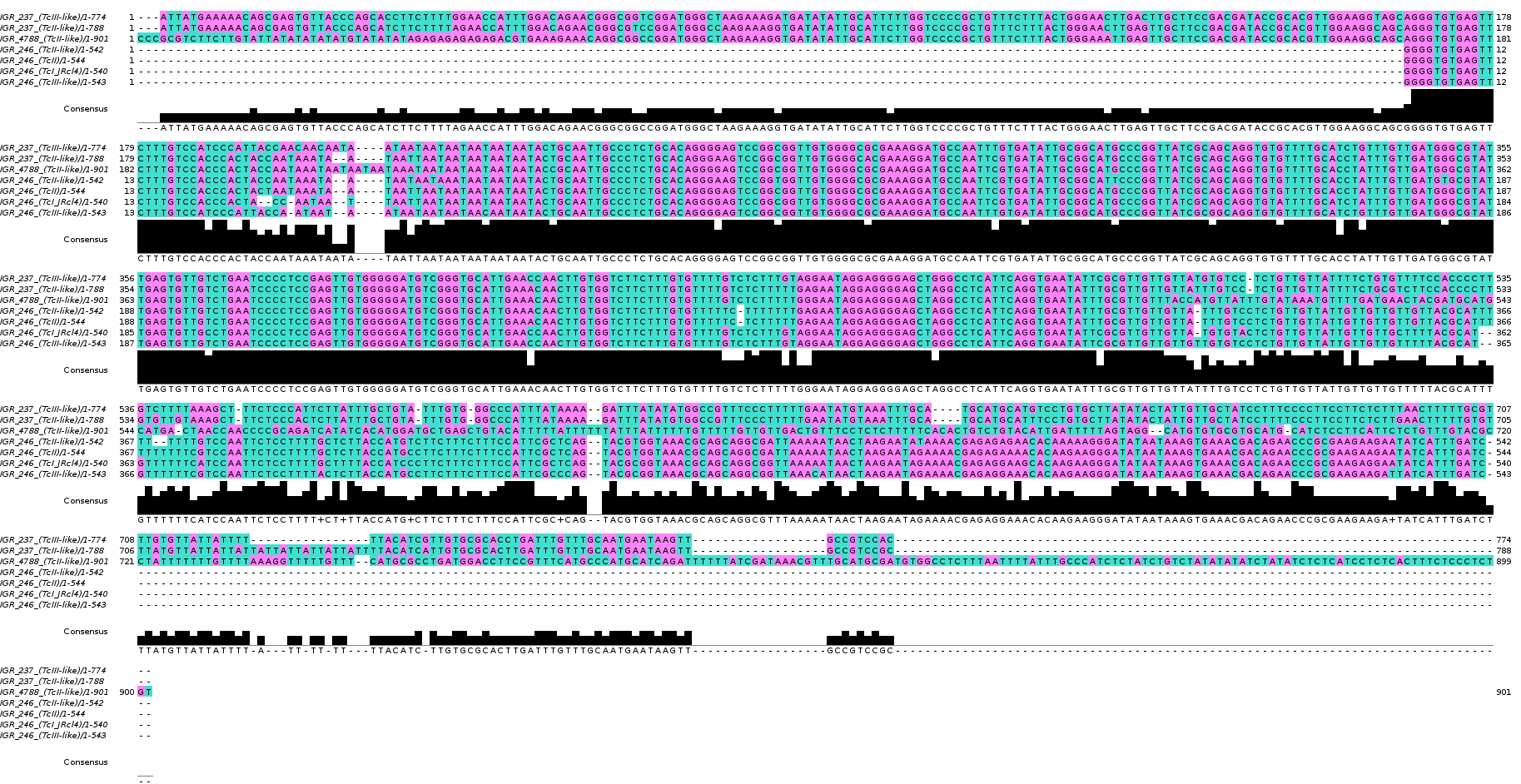

Supplement: Dataset S1 — Intergenic regions from unrelated loci that share blocks of significant sequence similarity. The file contains i) a spreadsheet summarizing listing the non-allelic, non-homologous IGR regions that share significant blocks of sequence similarity; ii) multiple sequence alignments where these unrelated IGRs were aligned to highlight the portion of the IGR that is shared; and iii) a figure in PDF format that shows the alignment in context. The alignments are provided in CLUSTAL format (.clw) and as colored renderings in PNG format, as produced by Jalview (purine/pyrimidine color scheme, only applied to regions of the alignment with 90% identity). The IGR IDs listed in each alignment correspond to those in Table S3. (ZIP) [file pntd.0002839.s010.zip › Dataset S1/alignment-pictures/entire-IGR/IGR_Group_1.png]

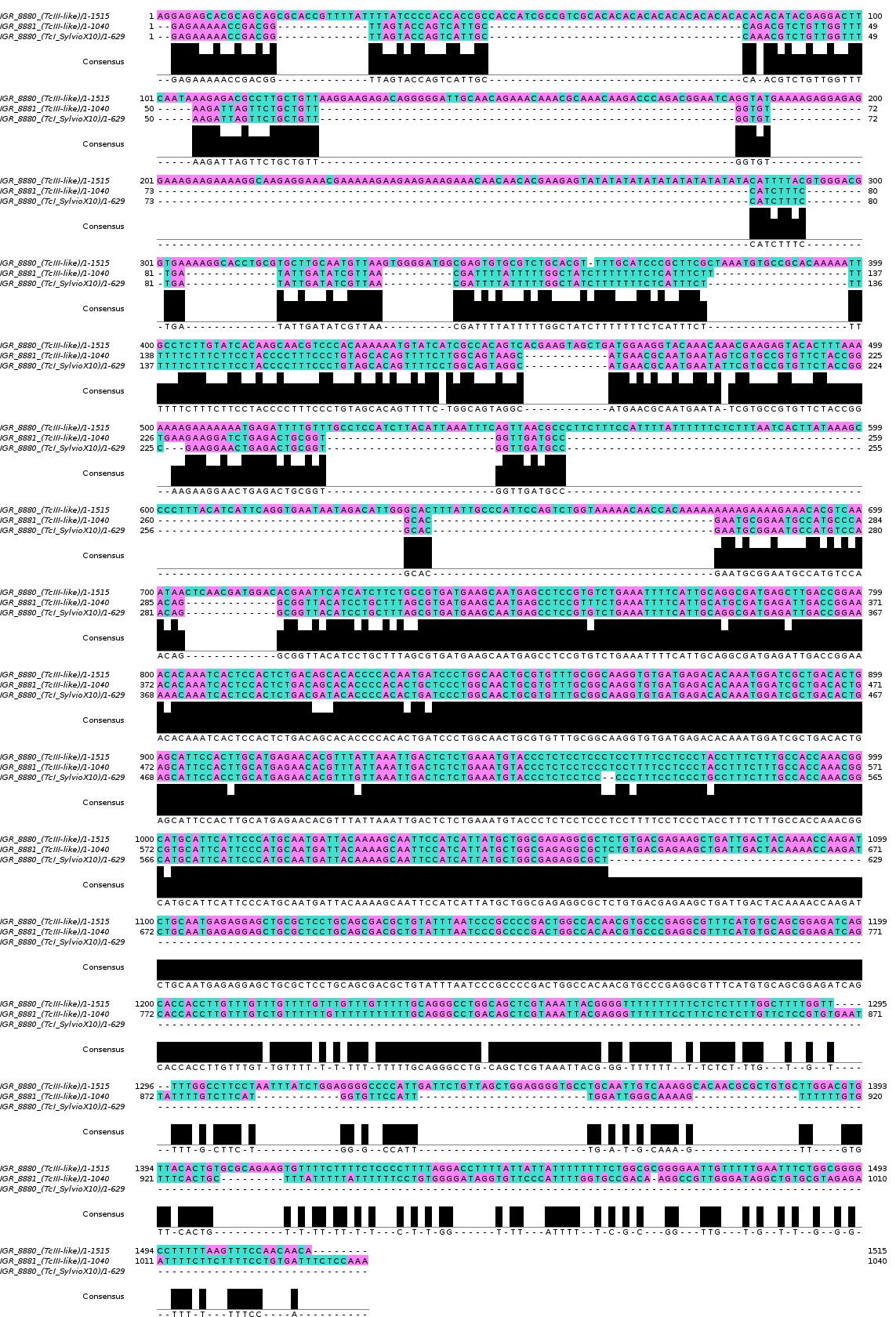

Supplement: Dataset S1 — Intergenic regions from unrelated loci that share blocks of significant sequence similarity. The file contains i) a spreadsheet summarizing listing the non-allelic, non-homologous IGR regions that share significant blocks of sequence similarity; ii) multiple sequence alignments where these unrelated IGRs were aligned to highlight the portion of the IGR that is shared; and iii) a figure in PDF format that shows the alignment in context. The alignments are provided in CLUSTAL format (.clw) and as colored renderings in PNG format, as produced by Jalview (purine/pyrimidine color scheme, only applied to regions of the alignment with 90% identity). The IGR IDs listed in each alignment correspond to those in Table S3. (ZIP) [file pntd.0002839.s010.zip › Dataset S1/alignment-pictures/entire-IGR/IGR_Group_20.png]

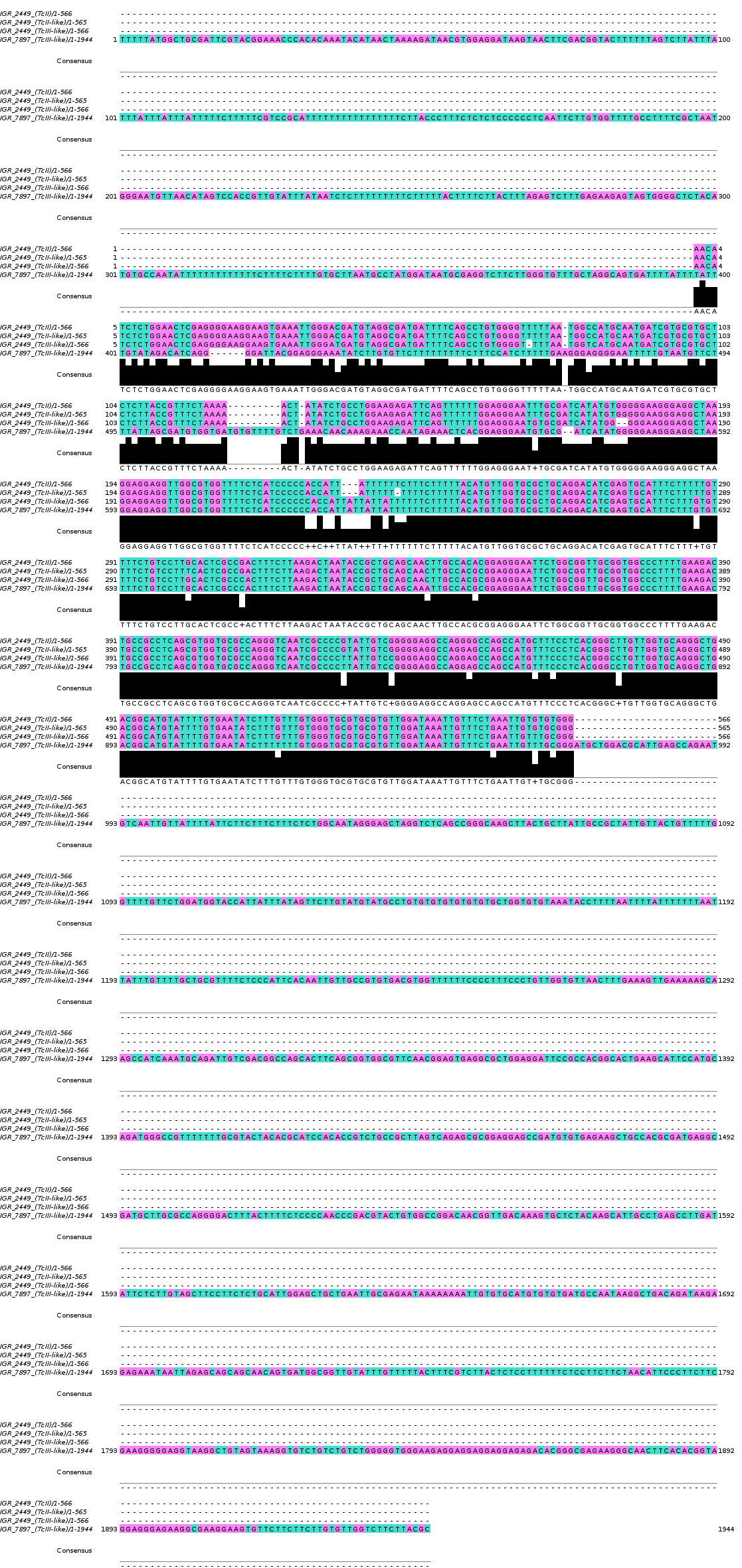

Supplement: Dataset S1 — Intergenic regions from unrelated loci that share blocks of significant sequence similarity. The file contains i) a spreadsheet summarizing listing the non-allelic, non-homologous IGR regions that share significant blocks of sequence similarity; ii) multiple sequence alignments where these unrelated IGRs were aligned to highlight the portion of the IGR that is shared; and iii) a figure in PDF format that shows the alignment in context. The alignments are provided in CLUSTAL format (.clw) and as colored renderings in PNG format, as produced by Jalview (purine/pyrimidine color scheme, only applied to regions of the alignment with 90% identity). The IGR IDs listed in each alignment correspond to those in Table S3. (ZIP) [file pntd.0002839.s010.zip › Dataset S1/alignment-pictures/entire-IGR/IGR_Group_11.png]

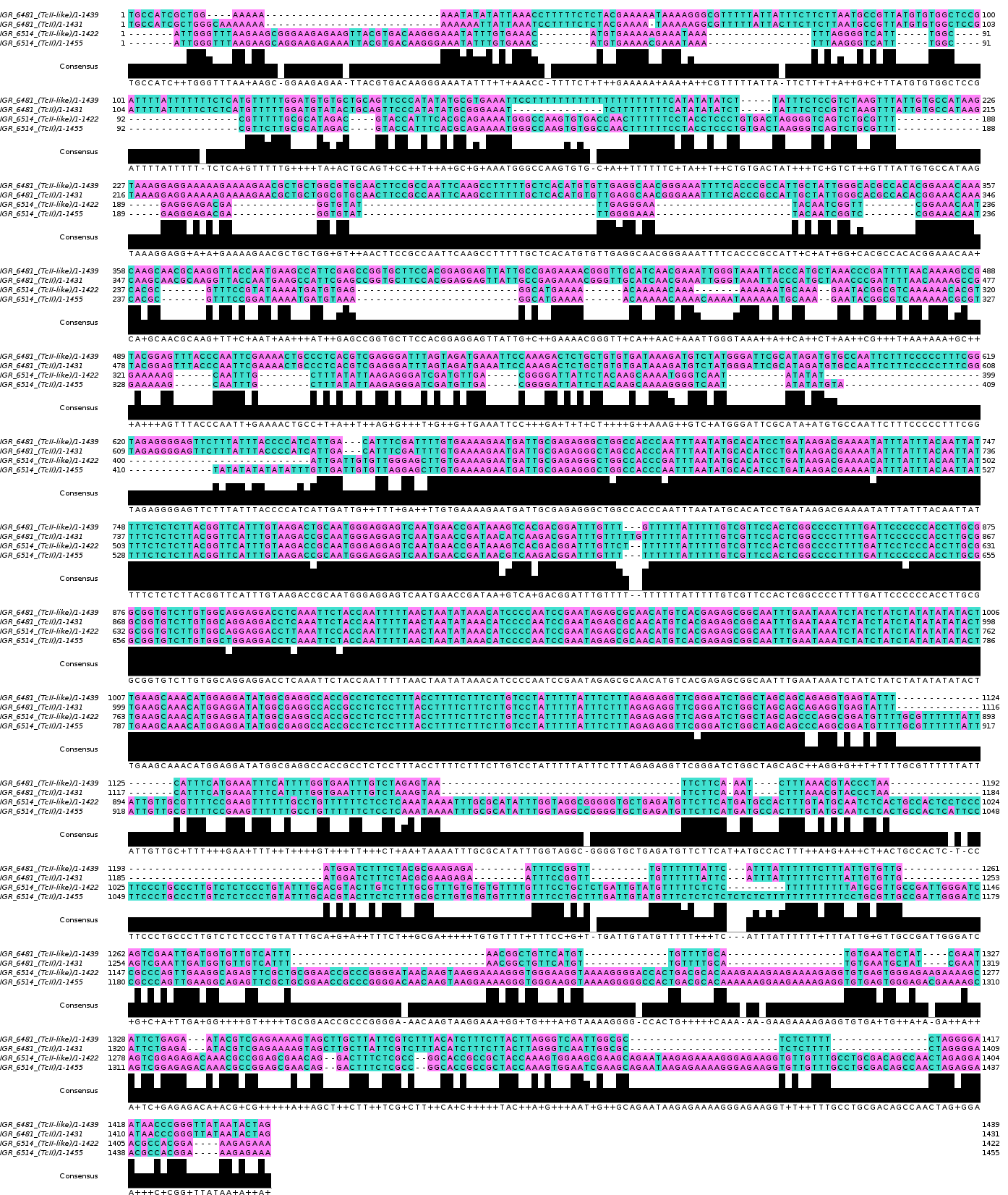

Supplement: Dataset S1 — Intergenic regions from unrelated loci that share blocks of significant sequence similarity. The file contains i) a spreadsheet summarizing listing the non-allelic, non-homologous IGR regions that share significant blocks of sequence similarity; ii) multiple sequence alignments where these unrelated IGRs were aligned to highlight the portion of the IGR that is shared; and iii) a figure in PDF format that shows the alignment in context. The alignments are provided in CLUSTAL format (.clw) and as colored renderings in PNG format, as produced by Jalview (purine/pyrimidine color scheme, only applied to regions of the alignment with 90% identity). The IGR IDs listed in each alignment correspond to those in Table S3. (ZIP) [file pntd.0002839.s010.zip › Dataset S1/alignment-pictures/entire-IGR/IGR_Group_16.png]

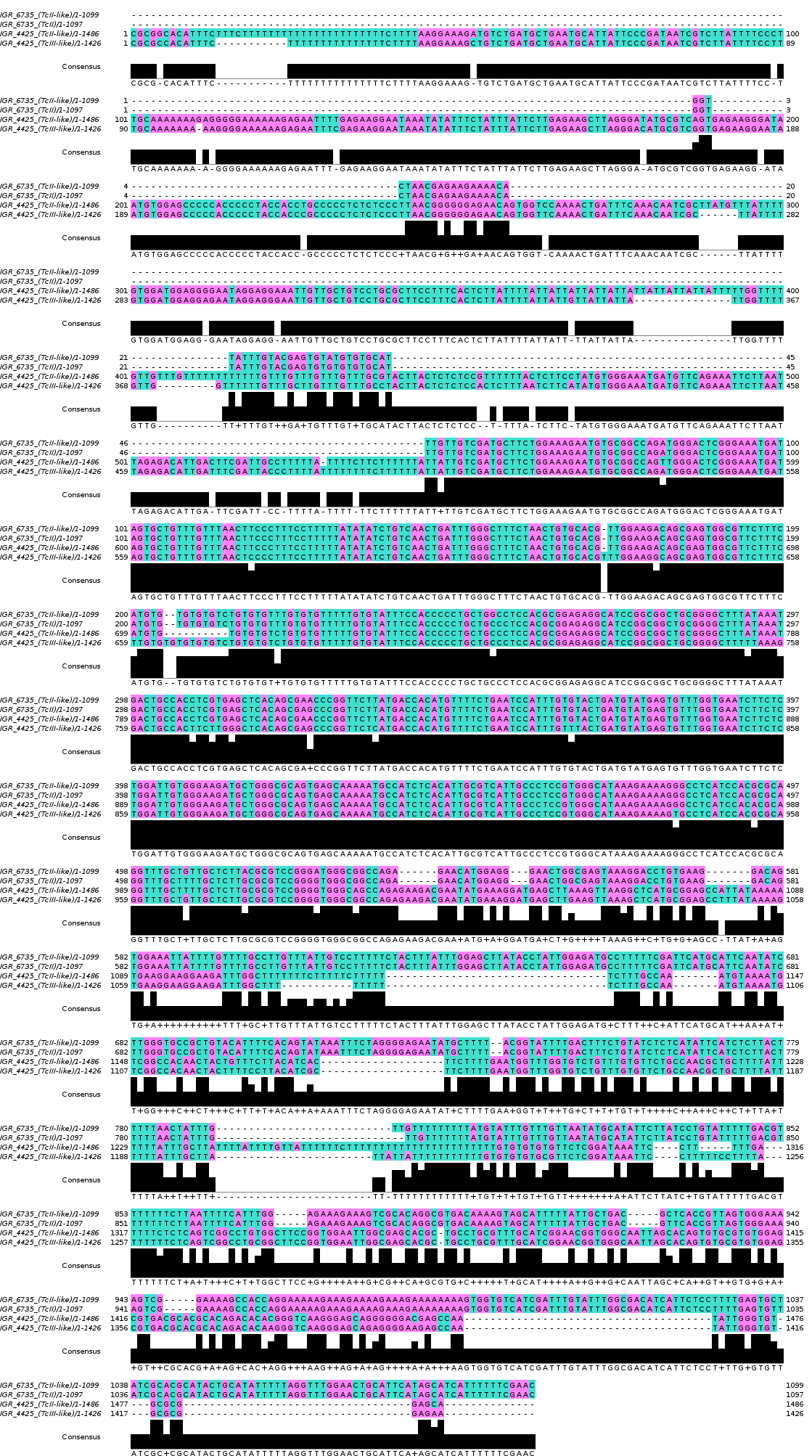

Supplement: Dataset S1 — Intergenic regions from unrelated loci that share blocks of significant sequence similarity. The file contains i) a spreadsheet summarizing listing the non-allelic, non-homologous IGR regions that share significant blocks of sequence similarity; ii) multiple sequence alignments where these unrelated IGRs were aligned to highlight the portion of the IGR that is shared; and iii) a figure in PDF format that shows the alignment in context. The alignments are provided in CLUSTAL format (.clw) and as colored renderings in PNG format, as produced by Jalview (purine/pyrimidine color scheme, only applied to regions of the alignment with 90% identity). The IGR IDs listed in each alignment correspond to those in Table S3. (ZIP) [file pntd.0002839.s010.zip › Dataset S1/alignment-pictures/entire-IGR/IGR_Group_15.png]

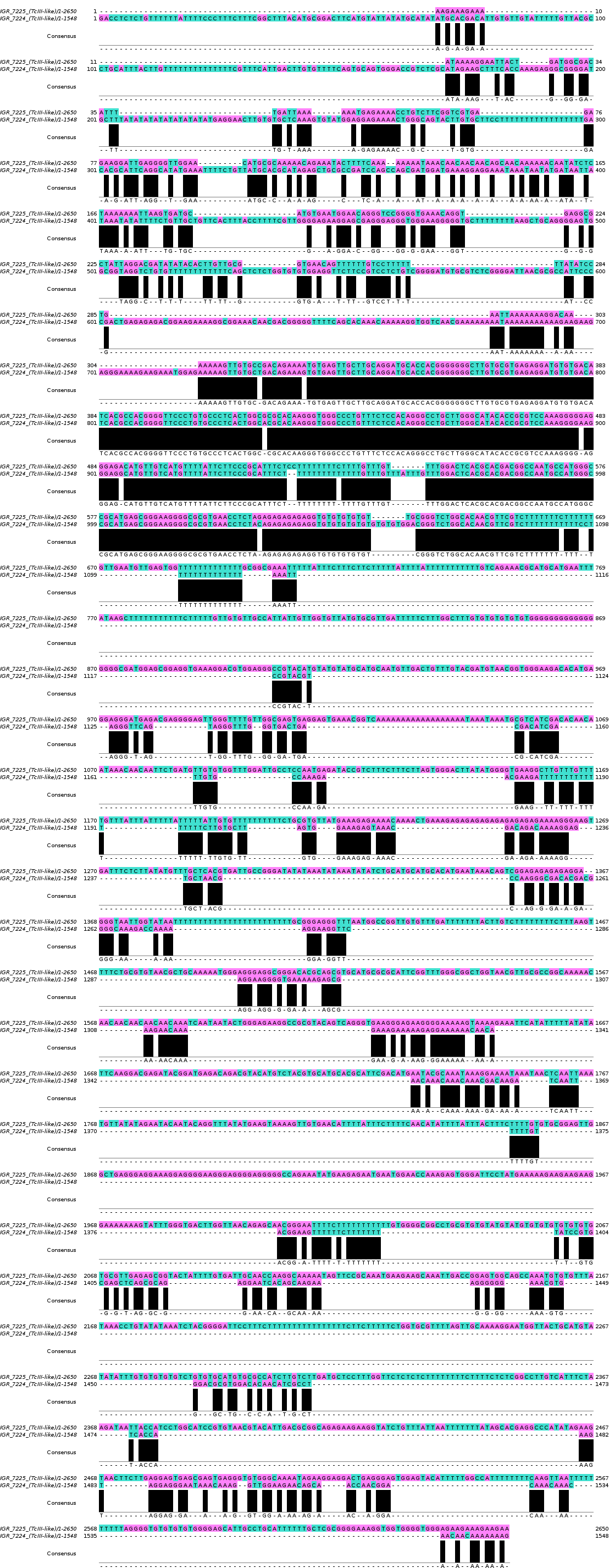

Supplement: Dataset S1 — Intergenic regions from unrelated loci that share blocks of significant sequence similarity. The file contains i) a spreadsheet summarizing listing the non-allelic, non-homologous IGR regions that share significant blocks of sequence similarity; ii) multiple sequence alignments where these unrelated IGRs were aligned to highlight the portion of the IGR that is shared; and iii) a figure in PDF format that shows the alignment in context. The alignments are provided in CLUSTAL format (.clw) and as colored renderings in PNG format, as produced by Jalview (purine/pyrimidine color scheme, only applied to regions of the alignment with 90% identity). The IGR IDs listed in each alignment correspond to those in Table S3. (ZIP) [file pntd.0002839.s010.zip › Dataset S1/alignment-pictures/entire-IGR/IGR_Group_18.png]

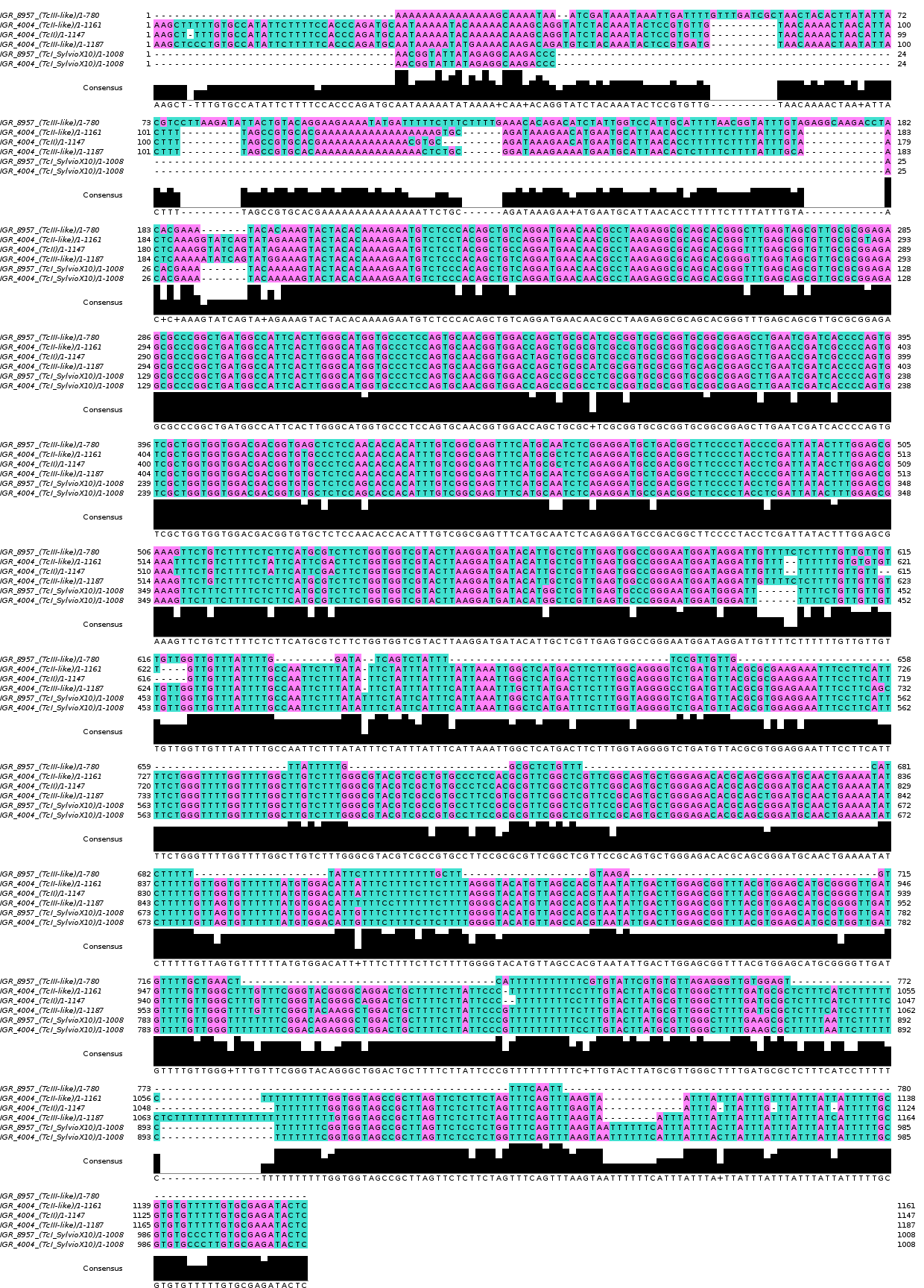

Supplement: Dataset S1 — Intergenic regions from unrelated loci that share blocks of significant sequence similarity. The file contains i) a spreadsheet summarizing listing the non-allelic, non-homologous IGR regions that share significant blocks of sequence similarity; ii) multiple sequence alignments where these unrelated IGRs were aligned to highlight the portion of the IGR that is shared; and iii) a figure in PDF format that shows the alignment in context. The alignments are provided in CLUSTAL format (.clw) and as colored renderings in PNG format, as produced by Jalview (purine/pyrimidine color scheme, only applied to regions of the alignment with 90% identity). The IGR IDs listed in each alignment correspond to those in Table S3. (ZIP) [file pntd.0002839.s010.zip › Dataset S1/alignment-pictures/entire-IGR/IGR_Group_13.png]

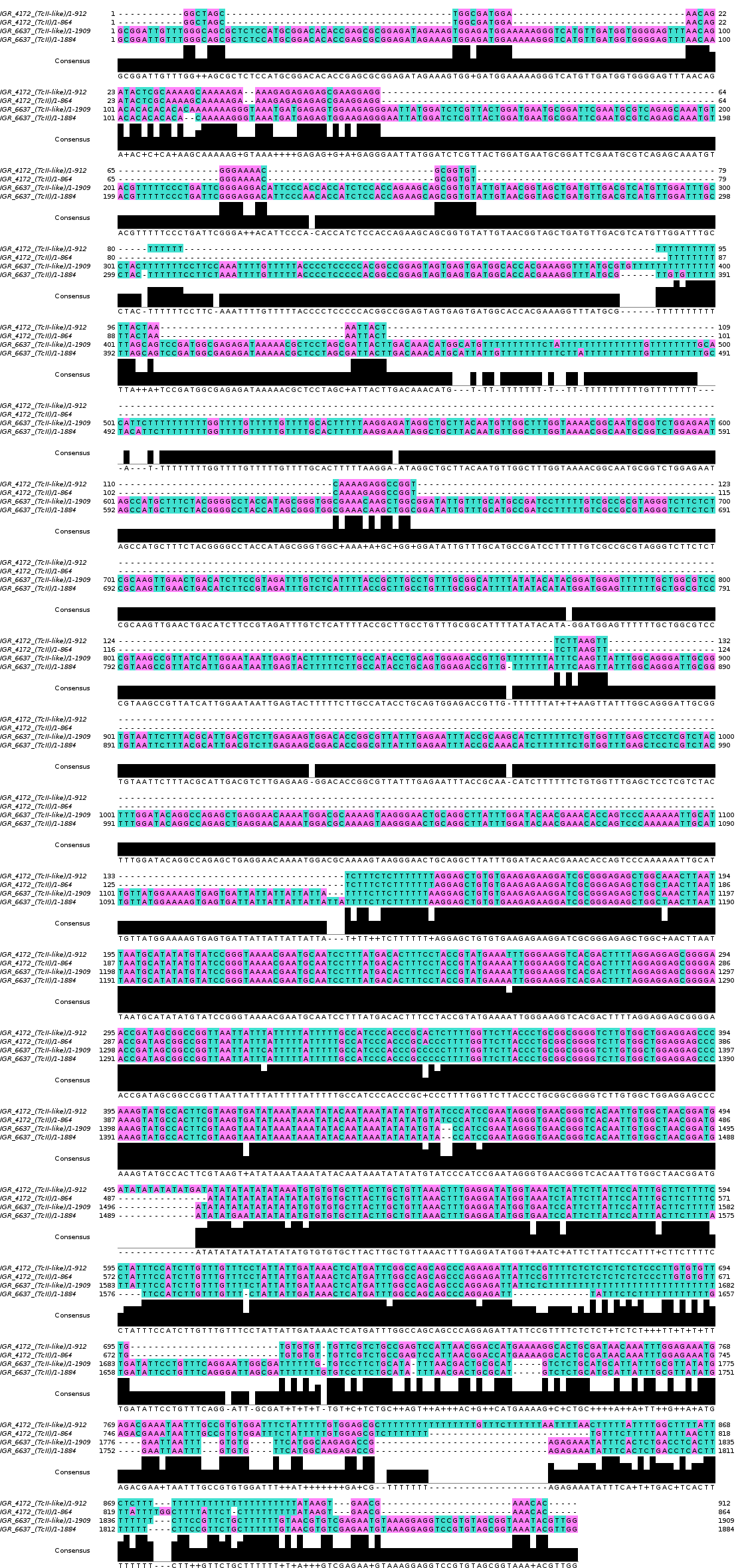

Supplement: Dataset S1 — Intergenic regions from unrelated loci that share blocks of significant sequence similarity. The file contains i) a spreadsheet summarizing listing the non-allelic, non-homologous IGR regions that share significant blocks of sequence similarity; ii) multiple sequence alignments where these unrelated IGRs were aligned to highlight the portion of the IGR that is shared; and iii) a figure in PDF format that shows the alignment in context. The alignments are provided in CLUSTAL format (.clw) and as colored renderings in PNG format, as produced by Jalview (purine/pyrimidine color scheme, only applied to regions of the alignment with 90% identity). The IGR IDs listed in each alignment correspond to those in Table S3. (ZIP) [file pntd.0002839.s010.zip › Dataset S1/alignment-pictures/entire-IGR/IGR_Group_21.png]

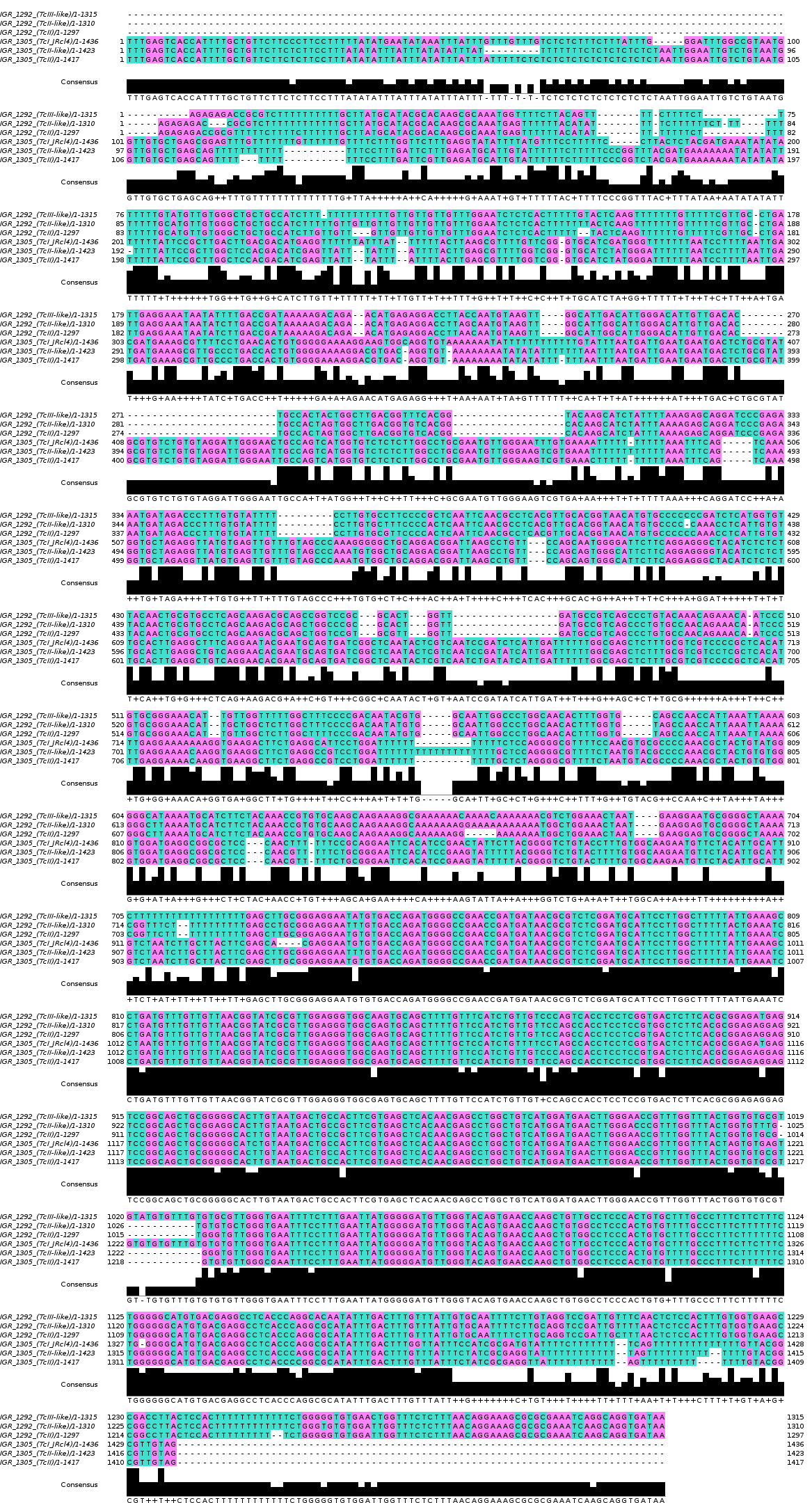

Supplement: Dataset S1 — Intergenic regions from unrelated loci that share blocks of significant sequence similarity. The file contains i) a spreadsheet summarizing listing the non-allelic, non-homologous IGR regions that share significant blocks of sequence similarity; ii) multiple sequence alignments where these unrelated IGRs were aligned to highlight the portion of the IGR that is shared; and iii) a figure in PDF format that shows the alignment in context. The alignments are provided in CLUSTAL format (.clw) and as colored renderings in PNG format, as produced by Jalview (purine/pyrimidine color scheme, only applied to regions of the alignment with 90% identity). The IGR IDs listed in each alignment correspond to those in Table S3. (ZIP) [file pntd.0002839.s010.zip › Dataset S1/alignment-pictures/entire-IGR/IGR_Group_10.png]

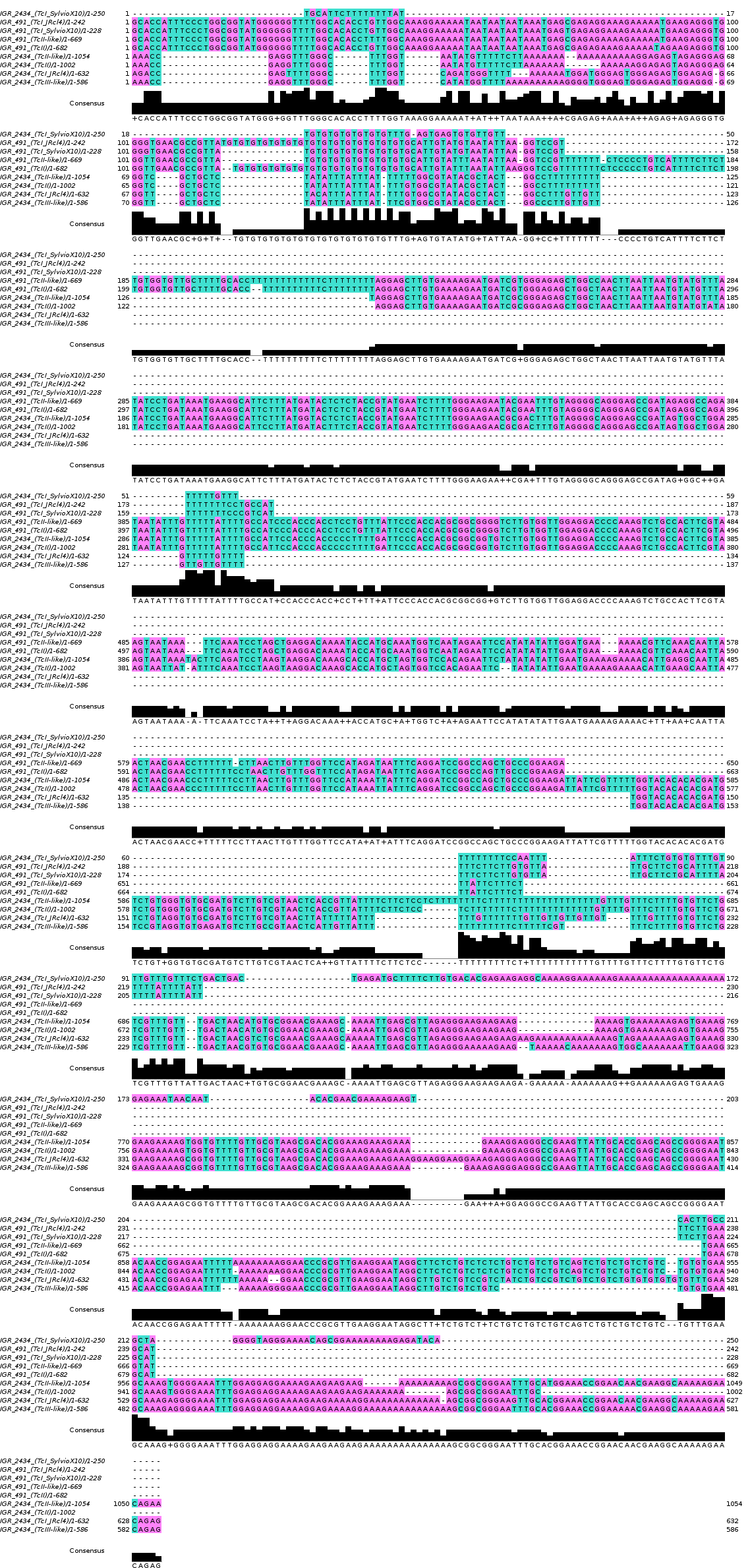

Supplement: Dataset S1 — Intergenic regions from unrelated loci that share blocks of significant sequence similarity. The file contains i) a spreadsheet summarizing listing the non-allelic, non-homologous IGR regions that share significant blocks of sequence similarity; ii) multiple sequence alignments where these unrelated IGRs were aligned to highlight the portion of the IGR that is shared; and iii) a figure in PDF format that shows the alignment in context. The alignments are provided in CLUSTAL format (.clw) and as colored renderings in PNG format, as produced by Jalview (purine/pyrimidine color scheme, only applied to regions of the alignment with 90% identity). The IGR IDs listed in each alignment correspond to those in Table S3. (ZIP) [file pntd.0002839.s010.zip › Dataset S1/alignment-pictures/entire-IGR/IGR_Group_2.png]

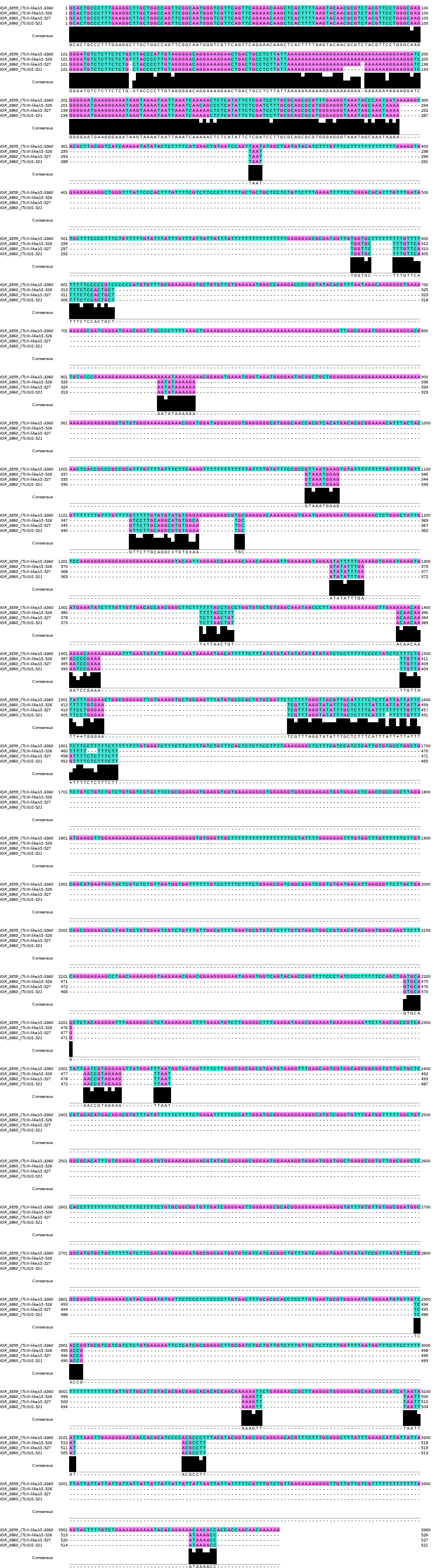

Supplement: Dataset S1 — Intergenic regions from unrelated loci that share blocks of significant sequence similarity. The file contains i) a spreadsheet summarizing listing the non-allelic, non-homologous IGR regions that share significant blocks of sequence similarity; ii) multiple sequence alignments where these unrelated IGRs were aligned to highlight the portion of the IGR that is shared; and iii) a figure in PDF format that shows the alignment in context. The alignments are provided in CLUSTAL format (.clw) and as colored renderings in PNG format, as produced by Jalview (purine/pyrimidine color scheme, only applied to regions of the alignment with 90% identity). The IGR IDs listed in each alignment correspond to those in Table S3. (ZIP) [file pntd.0002839.s010.zip › Dataset S1/alignment-pictures/entire-IGR/IGR_Group_12.png]

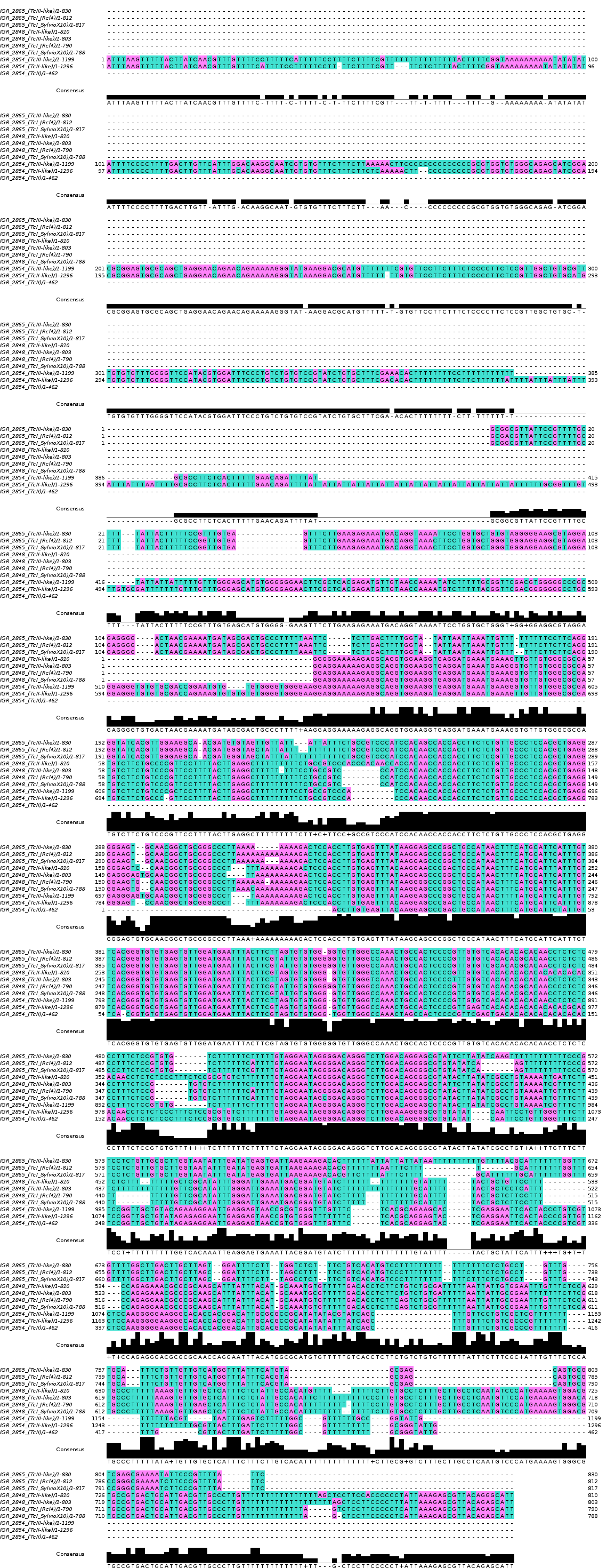

Supplement: Dataset S1 — Intergenic regions from unrelated loci that share blocks of significant sequence similarity. The file contains i) a spreadsheet summarizing listing the non-allelic, non-homologous IGR regions that share significant blocks of sequence similarity; ii) multiple sequence alignments where these unrelated IGRs were aligned to highlight the portion of the IGR that is shared; and iii) a figure in PDF format that shows the alignment in context. The alignments are provided in CLUSTAL format (.clw) and as colored renderings in PNG format, as produced by Jalview (purine/pyrimidine color scheme, only applied to regions of the alignment with 90% identity). The IGR IDs listed in each alignment correspond to those in Table S3. (ZIP) [file pntd.0002839.s010.zip › Dataset S1/alignment-pictures/entire-IGR/IGR_Group_5.png]

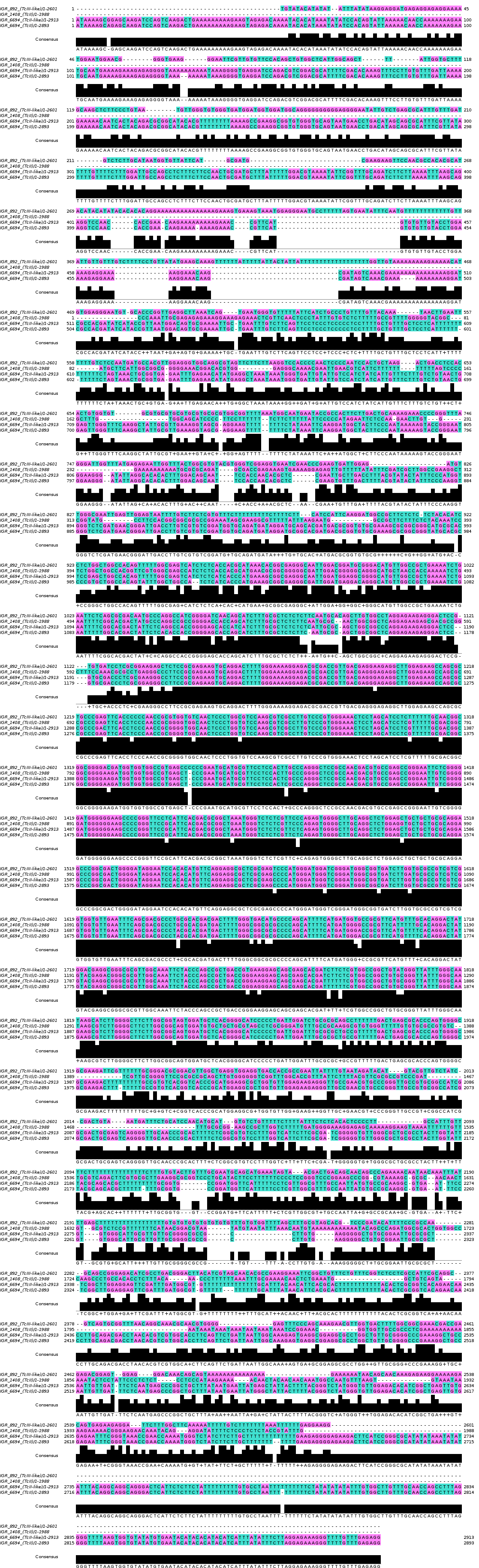

Supplement: Dataset S1 — Intergenic regions from unrelated loci that share blocks of significant sequence similarity. The file contains i) a spreadsheet summarizing listing the non-allelic, non-homologous IGR regions that share significant blocks of sequence similarity; ii) multiple sequence alignments where these unrelated IGRs were aligned to highlight the portion of the IGR that is shared; and iii) a figure in PDF format that shows the alignment in context. The alignments are provided in CLUSTAL format (.clw) and as colored renderings in PNG format, as produced by Jalview (purine/pyrimidine color scheme, only applied to regions of the alignment with 90% identity). The IGR IDs listed in each alignment correspond to those in Table S3. (ZIP) [file pntd.0002839.s010.zip › Dataset S1/alignment-pictures/entire-IGR/IGR_Group_3.png]

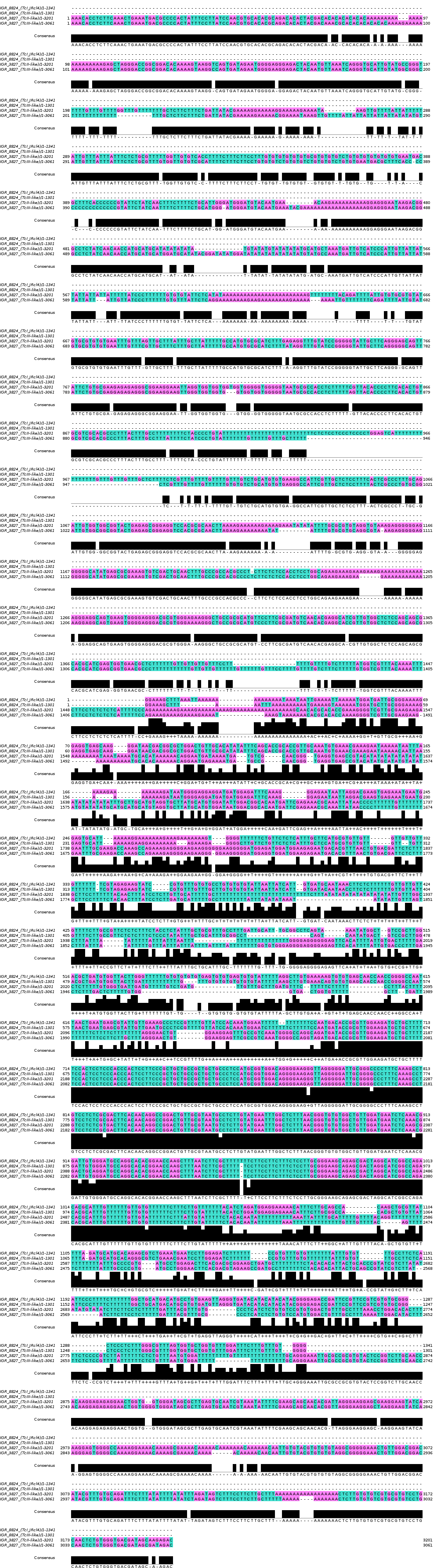

Supplement: Dataset S1 — Intergenic regions from unrelated loci that share blocks of significant sequence similarity. The file contains i) a spreadsheet summarizing listing the non-allelic, non-homologous IGR regions that share significant blocks of sequence similarity; ii) multiple sequence alignments where these unrelated IGRs were aligned to highlight the portion of the IGR that is shared; and iii) a figure in PDF format that shows the alignment in context. The alignments are provided in CLUSTAL format (.clw) and as colored renderings in PNG format, as produced by Jalview (purine/pyrimidine color scheme, only applied to regions of the alignment with 90% identity). The IGR IDs listed in each alignment correspond to those in Table S3. (ZIP) [file pntd.0002839.s010.zip › Dataset S1/alignment-pictures/entire-IGR/IGR_Group_19.png]

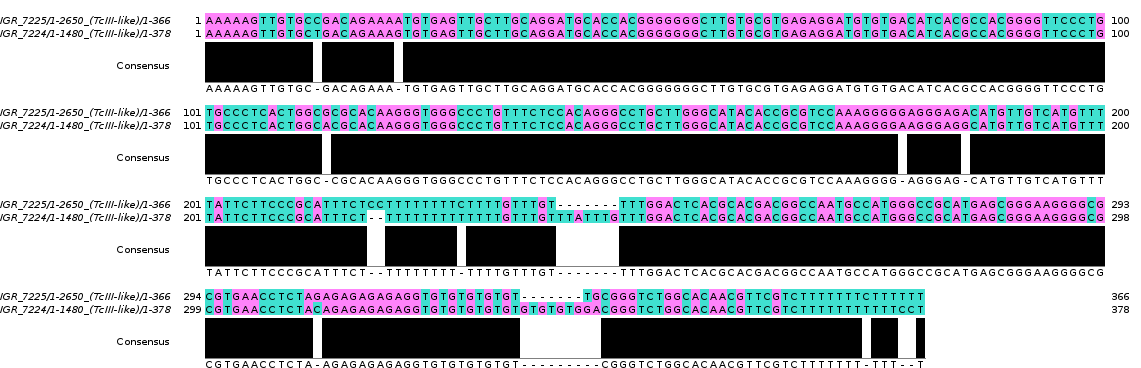

Supplement: Dataset S1 — Intergenic regions from unrelated loci that share blocks of significant sequence similarity. The file contains i) a spreadsheet summarizing listing the non-allelic, non-homologous IGR regions that share significant blocks of sequence similarity; ii) multiple sequence alignments where these unrelated IGRs were aligned to highlight the portion of the IGR that is shared; and iii) a figure in PDF format that shows the alignment in context. The alignments are provided in CLUSTAL format (.clw) and as colored renderings in PNG format, as produced by Jalview (purine/pyrimidine color scheme, only applied to regions of the alignment with 90% identity). The IGR IDs listed in each alignment correspond to those in Table S3. (ZIP) [file pntd.0002839.s010.zip › Dataset S1/alignment-pictures/motifs-only/Motif_18.png]

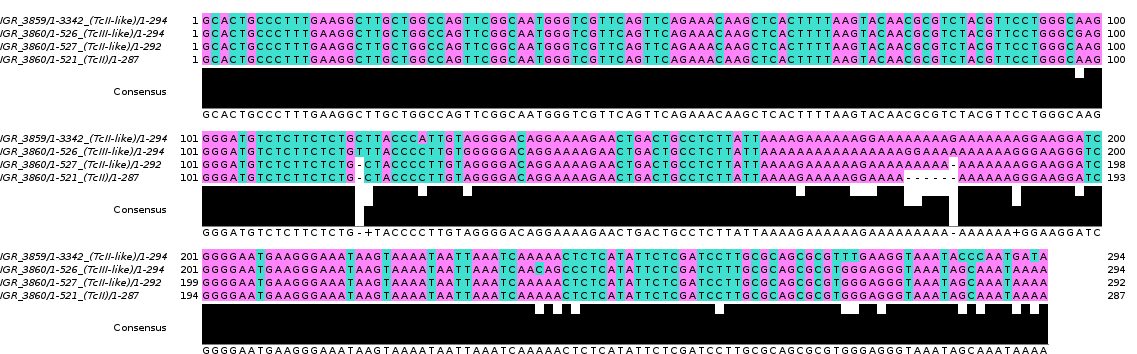

Supplement: Dataset S1 — Intergenic regions from unrelated loci that share blocks of significant sequence similarity. The file contains i) a spreadsheet summarizing listing the non-allelic, non-homologous IGR regions that share significant blocks of sequence similarity; ii) multiple sequence alignments where these unrelated IGRs were aligned to highlight the portion of the IGR that is shared; and iii) a figure in PDF format that shows the alignment in context. The alignments are provided in CLUSTAL format (.clw) and as colored renderings in PNG format, as produced by Jalview (purine/pyrimidine color scheme, only applied to regions of the alignment with 90% identity). The IGR IDs listed in each alignment correspond to those in Table S3. (ZIP) [file pntd.0002839.s010.zip › Dataset S1/alignment-pictures/motifs-only/Motif_12.png]

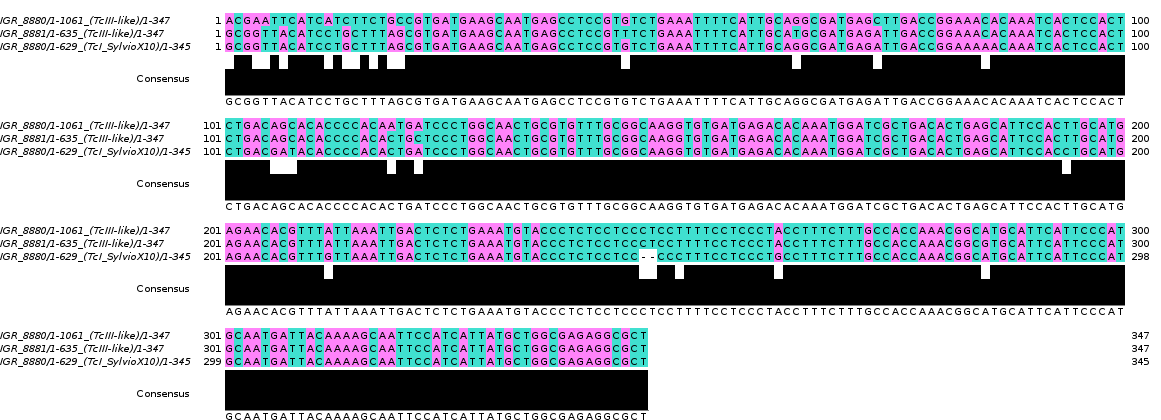

Supplement: Dataset S1 — Intergenic regions from unrelated loci that share blocks of significant sequence similarity. The file contains i) a spreadsheet summarizing listing the non-allelic, non-homologous IGR regions that share significant blocks of sequence similarity; ii) multiple sequence alignments where these unrelated IGRs were aligned to highlight the portion of the IGR that is shared; and iii) a figure in PDF format that shows the alignment in context. The alignments are provided in CLUSTAL format (.clw) and as colored renderings in PNG format, as produced by Jalview (purine/pyrimidine color scheme, only applied to regions of the alignment with 90% identity). The IGR IDs listed in each alignment correspond to those in Table S3. (ZIP) [file pntd.0002839.s010.zip › Dataset S1/alignment-pictures/motifs-only/Motif_20.png]

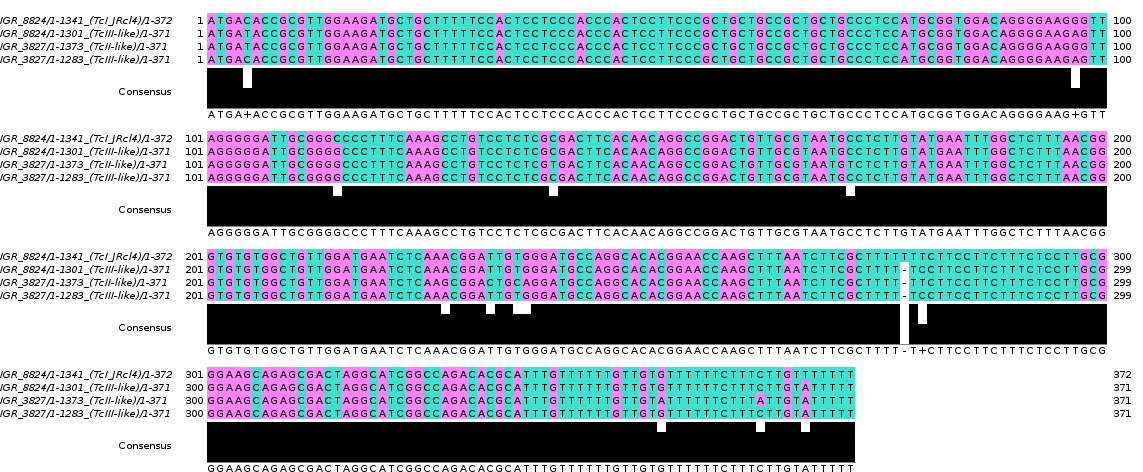

Supplement: Dataset S1 — Intergenic regions from unrelated loci that share blocks of significant sequence similarity. The file contains i) a spreadsheet summarizing listing the non-allelic, non-homologous IGR regions that share significant blocks of sequence similarity; ii) multiple sequence alignments where these unrelated IGRs were aligned to highlight the portion of the IGR that is shared; and iii) a figure in PDF format that shows the alignment in context. The alignments are provided in CLUSTAL format (.clw) and as colored renderings in PNG format, as produced by Jalview (purine/pyrimidine color scheme, only applied to regions of the alignment with 90% identity). The IGR IDs listed in each alignment correspond to those in Table S3. (ZIP) [file pntd.0002839.s010.zip › Dataset S1/alignment-pictures/motifs-only/Motif_19.png]

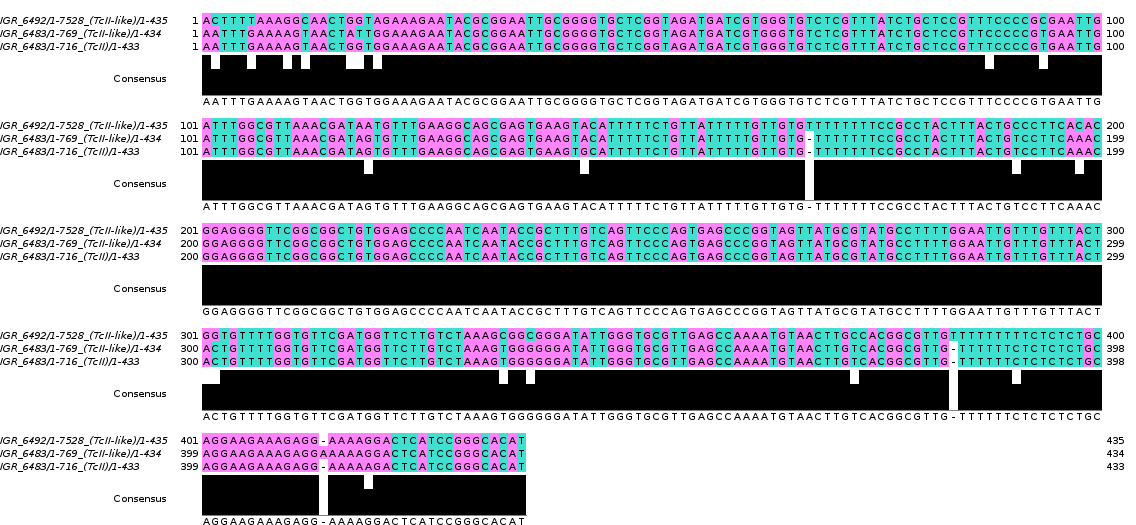

Supplement: Dataset S1 — Intergenic regions from unrelated loci that share blocks of significant sequence similarity. The file contains i) a spreadsheet summarizing listing the non-allelic, non-homologous IGR regions that share significant blocks of sequence similarity; ii) multiple sequence alignments where these unrelated IGRs were aligned to highlight the portion of the IGR that is shared; and iii) a figure in PDF format that shows the alignment in context. The alignments are provided in CLUSTAL format (.clw) and as colored renderings in PNG format, as produced by Jalview (purine/pyrimidine color scheme, only applied to regions of the alignment with 90% identity). The IGR IDs listed in each alignment correspond to those in Table S3. (ZIP) [file pntd.0002839.s010.zip › Dataset S1/alignment-pictures/motifs-only/Motif_17.png]

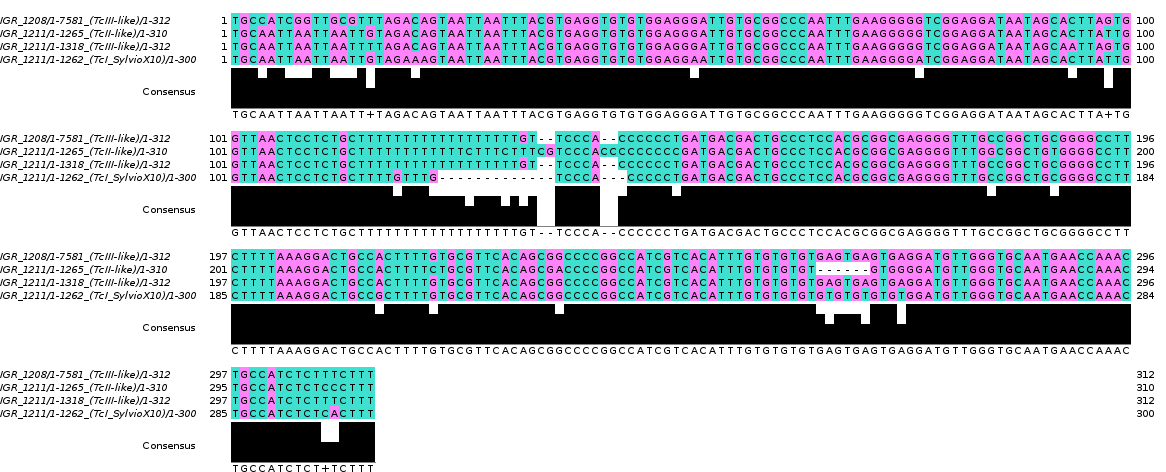

Supplement: Dataset S1 — Intergenic regions from unrelated loci that share blocks of significant sequence similarity. The file contains i) a spreadsheet summarizing listing the non-allelic, non-homologous IGR regions that share significant blocks of sequence similarity; ii) multiple sequence alignments where these unrelated IGRs were aligned to highlight the portion of the IGR that is shared; and iii) a figure in PDF format that shows the alignment in context. The alignments are provided in CLUSTAL format (.clw) and as colored renderings in PNG format, as produced by Jalview (purine/pyrimidine color scheme, only applied to regions of the alignment with 90% identity). The IGR IDs listed in each alignment correspond to those in Table S3. (ZIP) [file pntd.0002839.s010.zip › Dataset S1/alignment-pictures/motifs-only/Motif_9.png]

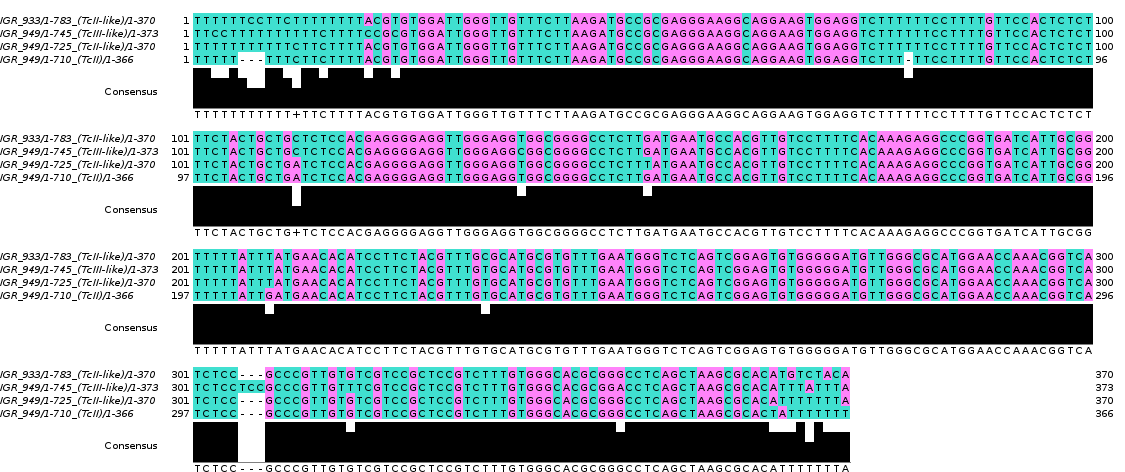

Supplement: Dataset S1 — Intergenic regions from unrelated loci that share blocks of significant sequence similarity. The file contains i) a spreadsheet summarizing listing the non-allelic, non-homologous IGR regions that share significant blocks of sequence similarity; ii) multiple sequence alignments where these unrelated IGRs were aligned to highlight the portion of the IGR that is shared; and iii) a figure in PDF format that shows the alignment in context. The alignments are provided in CLUSTAL format (.clw) and as colored renderings in PNG format, as produced by Jalview (purine/pyrimidine color scheme, only applied to regions of the alignment with 90% identity). The IGR IDs listed in each alignment correspond to those in Table S3. (ZIP) [file pntd.0002839.s010.zip › Dataset S1/alignment-pictures/motifs-only/Motif_8.png]

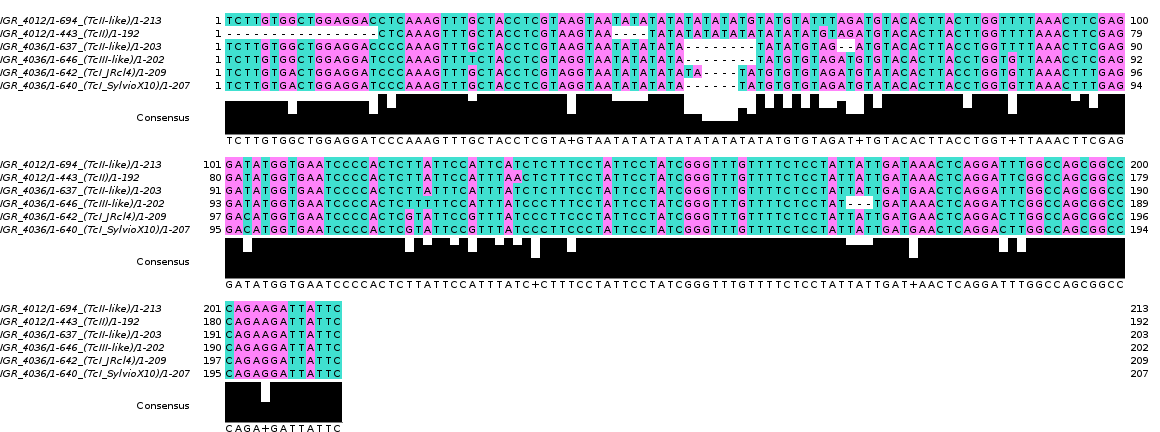

Supplement: Dataset S1 — Intergenic regions from unrelated loci that share blocks of significant sequence similarity. The file contains i) a spreadsheet summarizing listing the non-allelic, non-homologous IGR regions that share significant blocks of sequence similarity; ii) multiple sequence alignments where these unrelated IGRs were aligned to highlight the portion of the IGR that is shared; and iii) a figure in PDF format that shows the alignment in context. The alignments are provided in CLUSTAL format (.clw) and as colored renderings in PNG format, as produced by Jalview (purine/pyrimidine color scheme, only applied to regions of the alignment with 90% identity). The IGR IDs listed in each alignment correspond to those in Table S3. (ZIP) [file pntd.0002839.s010.zip › Dataset S1/alignment-pictures/motifs-only/Motif_14.png]

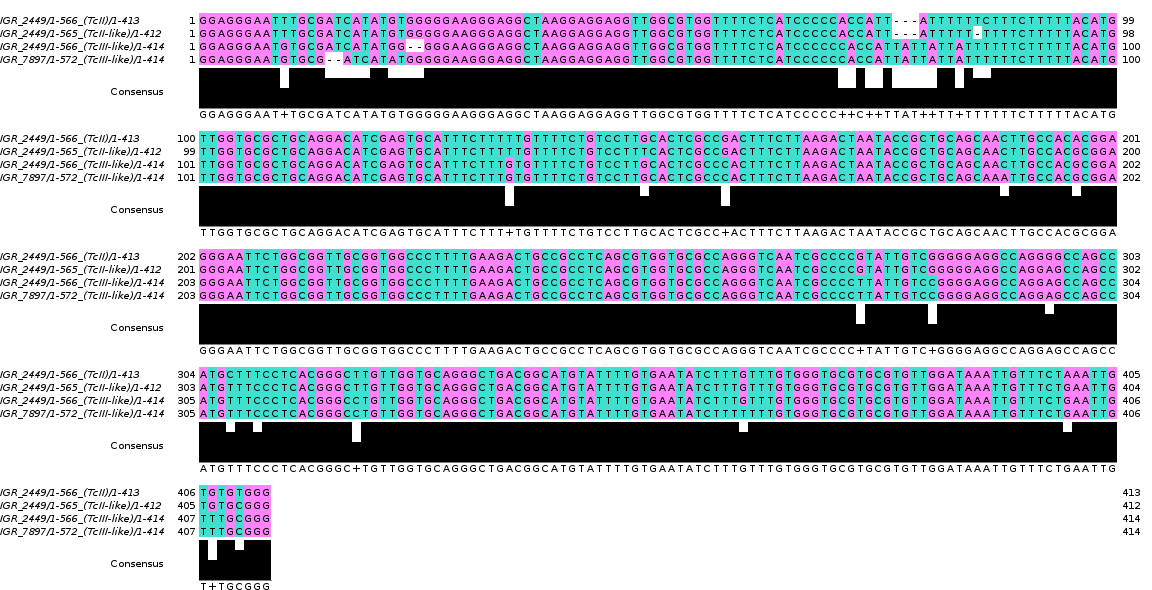

Supplement: Dataset S1 — Intergenic regions from unrelated loci that share blocks of significant sequence similarity. The file contains i) a spreadsheet summarizing listing the non-allelic, non-homologous IGR regions that share significant blocks of sequence similarity; ii) multiple sequence alignments where these unrelated IGRs were aligned to highlight the portion of the IGR that is shared; and iii) a figure in PDF format that shows the alignment in context. The alignments are provided in CLUSTAL format (.clw) and as colored renderings in PNG format, as produced by Jalview (purine/pyrimidine color scheme, only applied to regions of the alignment with 90% identity). The IGR IDs listed in each alignment correspond to those in Table S3. (ZIP) [file pntd.0002839.s010.zip › Dataset S1/alignment-pictures/motifs-only/Motif_11.png]

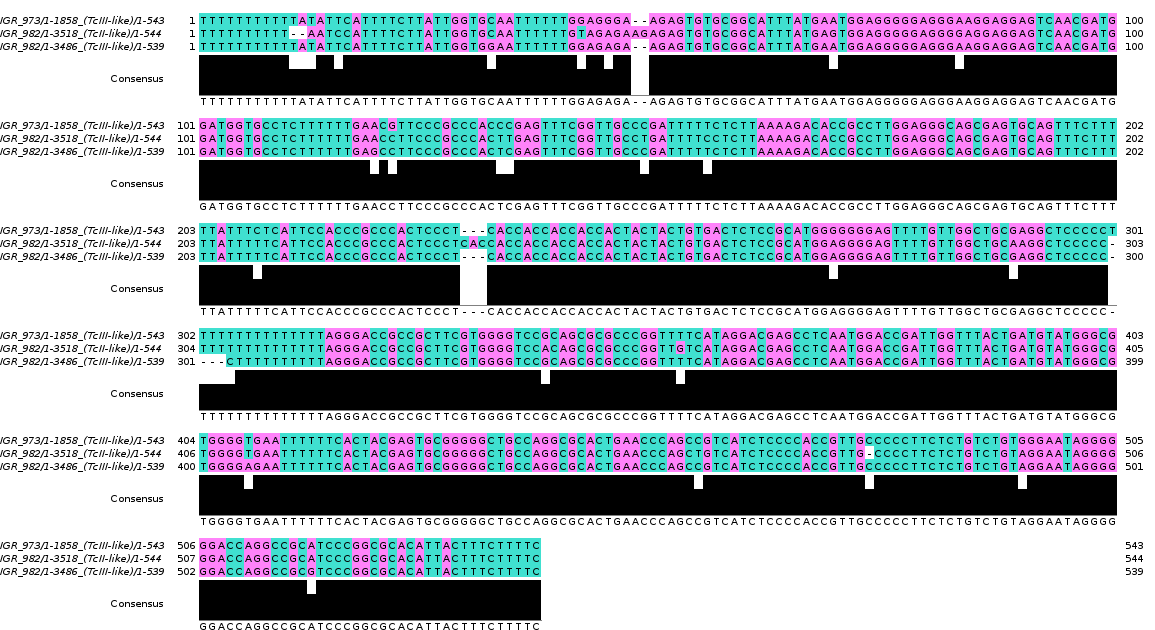

Supplement: Dataset S1 — Intergenic regions from unrelated loci that share blocks of significant sequence similarity. The file contains i) a spreadsheet summarizing listing the non-allelic, non-homologous IGR regions that share significant blocks of sequence similarity; ii) multiple sequence alignments where these unrelated IGRs were aligned to highlight the portion of the IGR that is shared; and iii) a figure in PDF format that shows the alignment in context. The alignments are provided in CLUSTAL format (.clw) and as colored renderings in PNG format, as produced by Jalview (purine/pyrimidine color scheme, only applied to regions of the alignment with 90% identity). The IGR IDs listed in each alignment correspond to those in Table S3. (ZIP) [file pntd.0002839.s010.zip › Dataset S1/alignment-pictures/motifs-only/Motif_7.png]

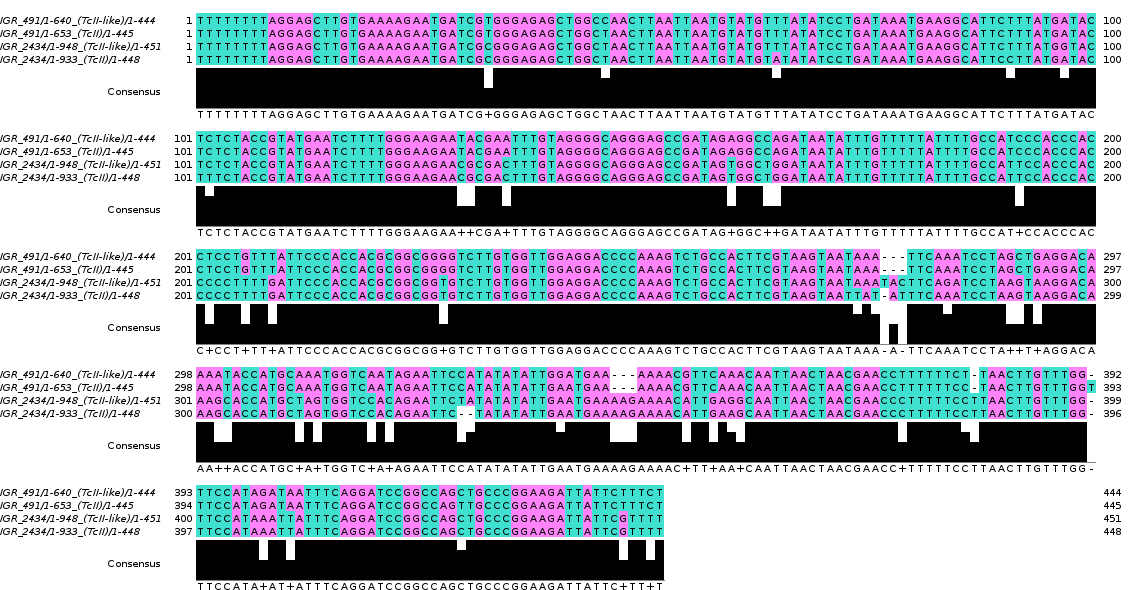

Supplement: Dataset S1 — Intergenic regions from unrelated loci that share blocks of significant sequence similarity. The file contains i) a spreadsheet summarizing listing the non-allelic, non-homologous IGR regions that share significant blocks of sequence similarity; ii) multiple sequence alignments where these unrelated IGRs were aligned to highlight the portion of the IGR that is shared; and iii) a figure in PDF format that shows the alignment in context. The alignments are provided in CLUSTAL format (.clw) and as colored renderings in PNG format, as produced by Jalview (purine/pyrimidine color scheme, only applied to regions of the alignment with 90% identity). The IGR IDs listed in each alignment correspond to those in Table S3. (ZIP) [file pntd.0002839.s010.zip › Dataset S1/alignment-pictures/motifs-only/Motif_2.png]

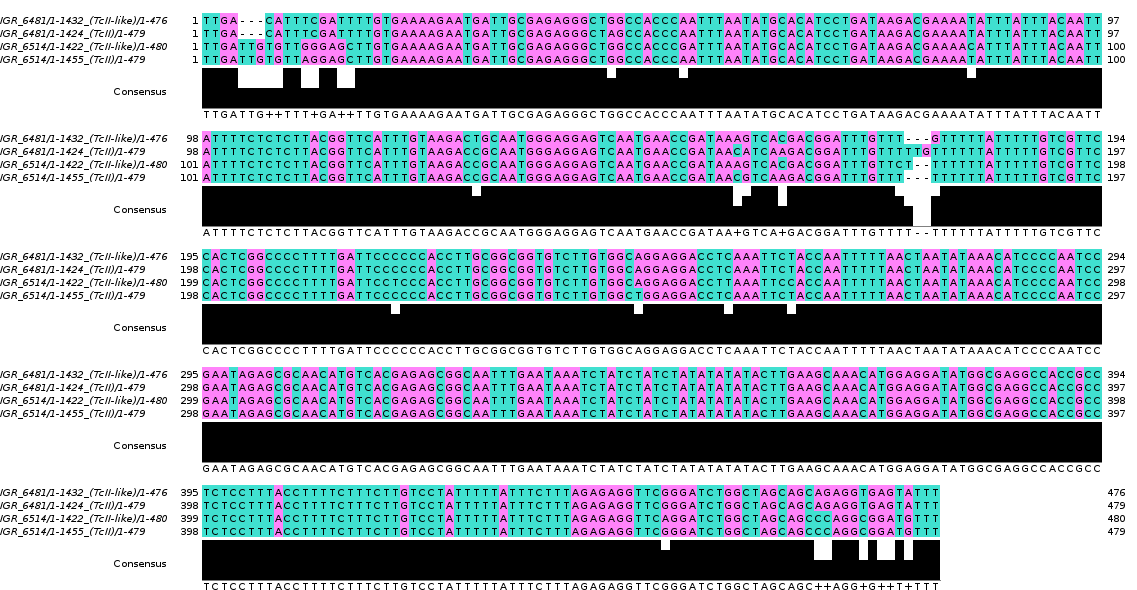

Supplement: Dataset S1 — Intergenic regions from unrelated loci that share blocks of significant sequence similarity. The file contains i) a spreadsheet summarizing listing the non-allelic, non-homologous IGR regions that share significant blocks of sequence similarity; ii) multiple sequence alignments where these unrelated IGRs were aligned to highlight the portion of the IGR that is shared; and iii) a figure in PDF format that shows the alignment in context. The alignments are provided in CLUSTAL format (.clw) and as colored renderings in PNG format, as produced by Jalview (purine/pyrimidine color scheme, only applied to regions of the alignment with 90% identity). The IGR IDs listed in each alignment correspond to those in Table S3. (ZIP) [file pntd.0002839.s010.zip › Dataset S1/alignment-pictures/motifs-only/Motif_16.png]

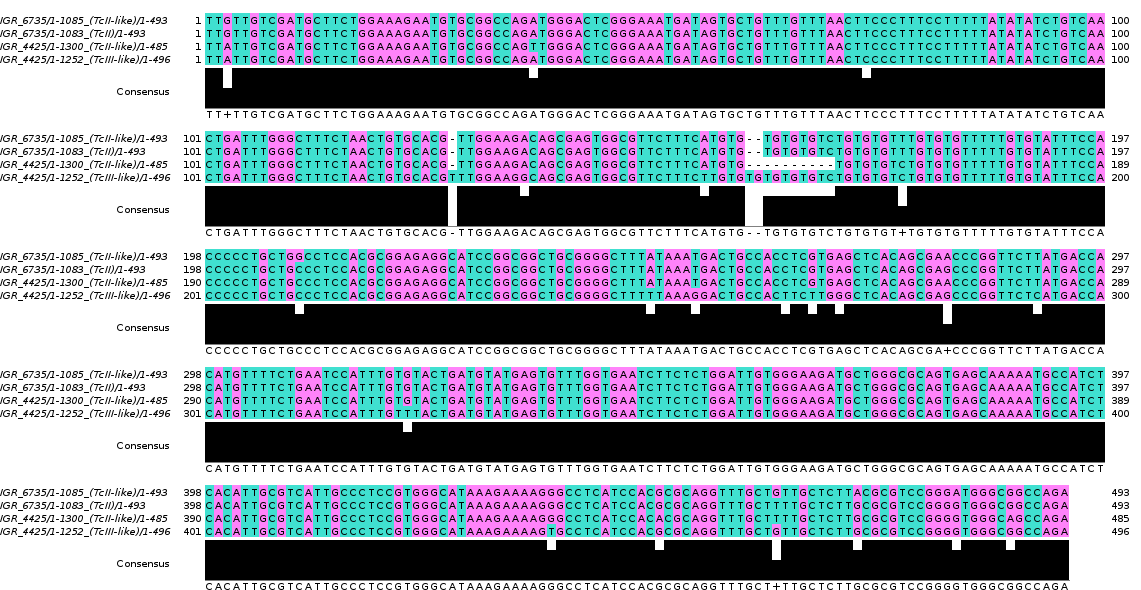

Supplement: Dataset S1 — Intergenic regions from unrelated loci that share blocks of significant sequence similarity. The file contains i) a spreadsheet summarizing listing the non-allelic, non-homologous IGR regions that share significant blocks of sequence similarity; ii) multiple sequence alignments where these unrelated IGRs were aligned to highlight the portion of the IGR that is shared; and iii) a figure in PDF format that shows the alignment in context. The alignments are provided in CLUSTAL format (.clw) and as colored renderings in PNG format, as produced by Jalview (purine/pyrimidine color scheme, only applied to regions of the alignment with 90% identity). The IGR IDs listed in each alignment correspond to those in Table S3. (ZIP) [file pntd.0002839.s010.zip › Dataset S1/alignment-pictures/motifs-only/Motif_15.png]

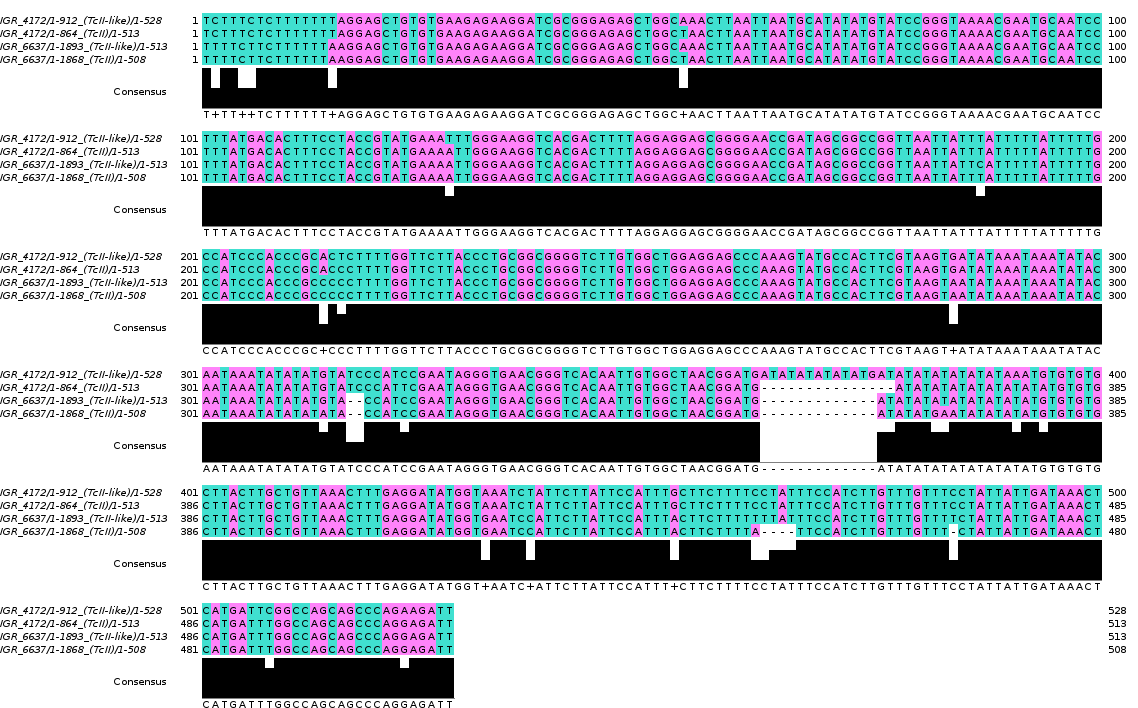

Supplement: Dataset S1 — Intergenic regions from unrelated loci that share blocks of significant sequence similarity. The file contains i) a spreadsheet summarizing listing the non-allelic, non-homologous IGR regions that share significant blocks of sequence similarity; ii) multiple sequence alignments where these unrelated IGRs were aligned to highlight the portion of the IGR that is shared; and iii) a figure in PDF format that shows the alignment in context. The alignments are provided in CLUSTAL format (.clw) and as colored renderings in PNG format, as produced by Jalview (purine/pyrimidine color scheme, only applied to regions of the alignment with 90% identity). The IGR IDs listed in each alignment correspond to those in Table S3. (ZIP) [file pntd.0002839.s010.zip › Dataset S1/alignment-pictures/motifs-only/Motif_21.png]

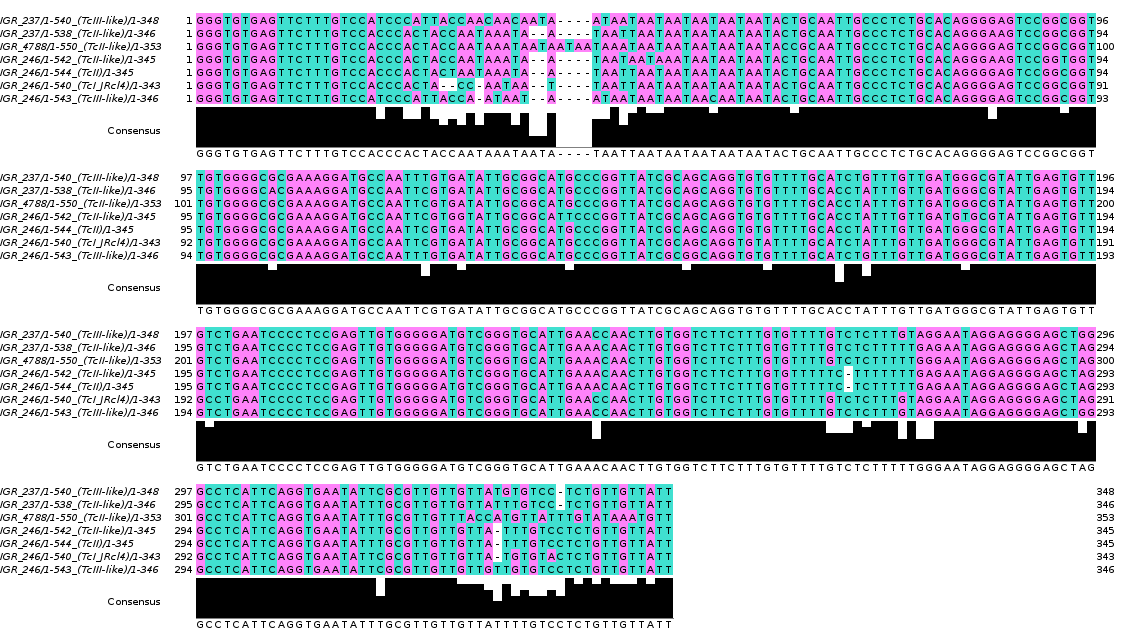

Supplement: Dataset S1 — Intergenic regions from unrelated loci that share blocks of significant sequence similarity. The file contains i) a spreadsheet summarizing listing the non-allelic, non-homologous IGR regions that share significant blocks of sequence similarity; ii) multiple sequence alignments where these unrelated IGRs were aligned to highlight the portion of the IGR that is shared; and iii) a figure in PDF format that shows the alignment in context. The alignments are provided in CLUSTAL format (.clw) and as colored renderings in PNG format, as produced by Jalview (purine/pyrimidine color scheme, only applied to regions of the alignment with 90% identity). The IGR IDs listed in each alignment correspond to those in Table S3. (ZIP) [file pntd.0002839.s010.zip › Dataset S1/alignment-pictures/motifs-only/Motif_1.png]

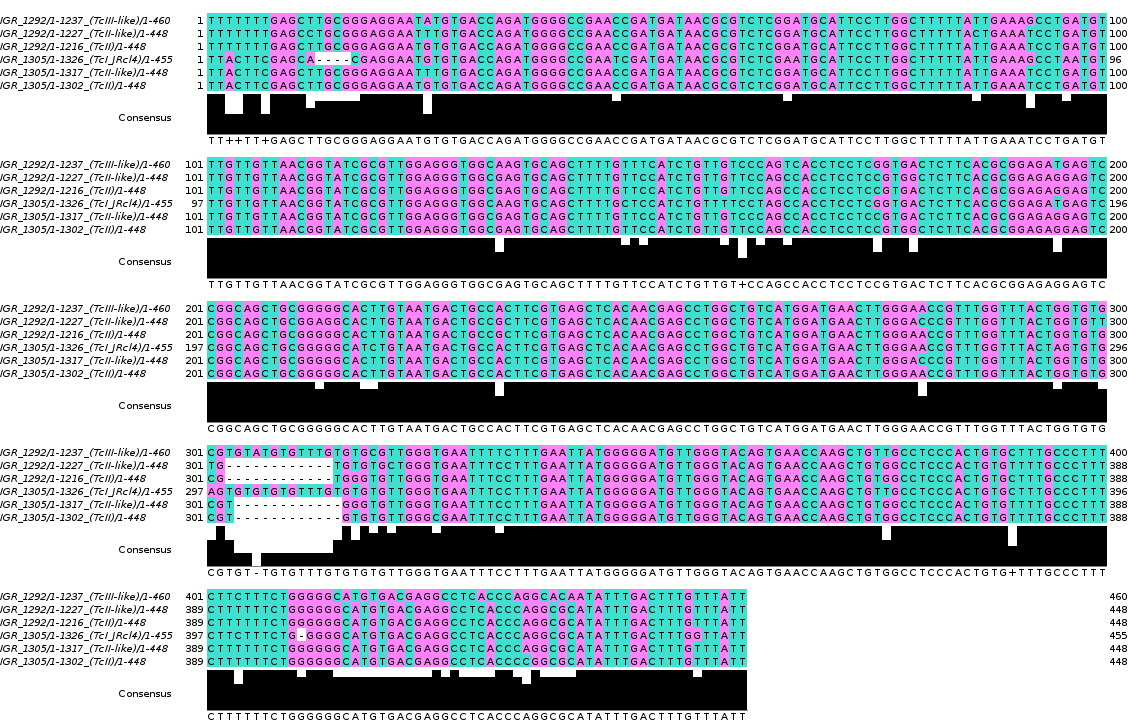

Supplement: Dataset S1 — Intergenic regions from unrelated loci that share blocks of significant sequence similarity. The file contains i) a spreadsheet summarizing listing the non-allelic, non-homologous IGR regions that share significant blocks of sequence similarity; ii) multiple sequence alignments where these unrelated IGRs were aligned to highlight the portion of the IGR that is shared; and iii) a figure in PDF format that shows the alignment in context. The alignments are provided in CLUSTAL format (.clw) and as colored renderings in PNG format, as produced by Jalview (purine/pyrimidine color scheme, only applied to regions of the alignment with 90% identity). The IGR IDs listed in each alignment correspond to those in Table S3. (ZIP) [file pntd.0002839.s010.zip › Dataset S1/alignment-pictures/motifs-only/Motif_10.png]

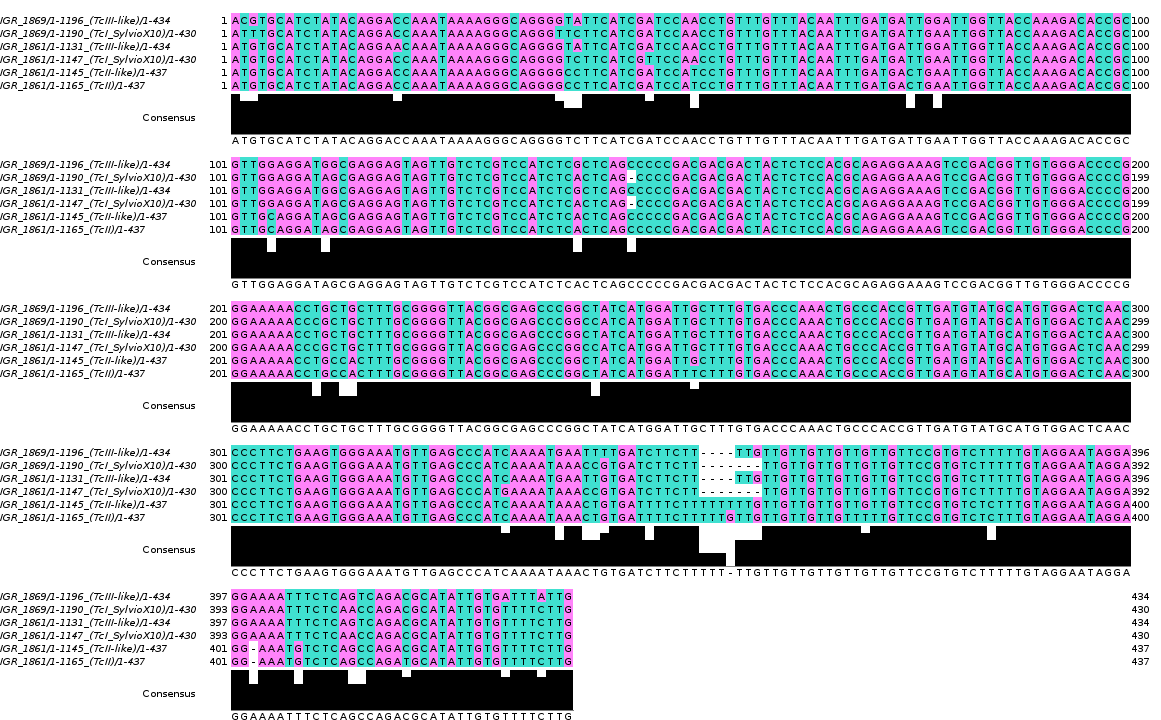

Supplement: Dataset S1 — Intergenic regions from unrelated loci that share blocks of significant sequence similarity. The file contains i) a spreadsheet summarizing listing the non-allelic, non-homologous IGR regions that share significant blocks of sequence similarity; ii) multiple sequence alignments where these unrelated IGRs were aligned to highlight the portion of the IGR that is shared; and iii) a figure in PDF format that shows the alignment in context. The alignments are provided in CLUSTAL format (.clw) and as colored renderings in PNG format, as produced by Jalview (purine/pyrimidine color scheme, only applied to regions of the alignment with 90% identity). The IGR IDs listed in each alignment correspond to those in Table S3. (ZIP) [file pntd.0002839.s010.zip › Dataset S1/alignment-pictures/motifs-only/Motif_4.png]

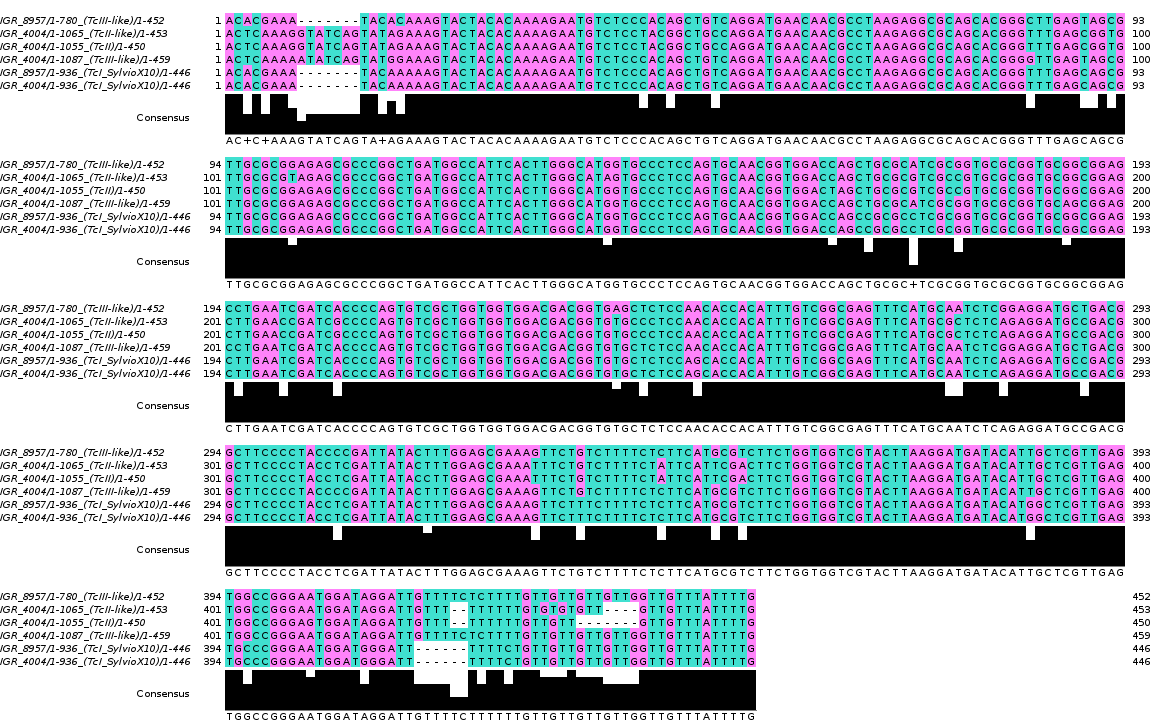

Supplement: Dataset S1 — Intergenic regions from unrelated loci that share blocks of significant sequence similarity. The file contains i) a spreadsheet summarizing listing the non-allelic, non-homologous IGR regions that share significant blocks of sequence similarity; ii) multiple sequence alignments where these unrelated IGRs were aligned to highlight the portion of the IGR that is shared; and iii) a figure in PDF format that shows the alignment in context. The alignments are provided in CLUSTAL format (.clw) and as colored renderings in PNG format, as produced by Jalview (purine/pyrimidine color scheme, only applied to regions of the alignment with 90% identity). The IGR IDs listed in each alignment correspond to those in Table S3. (ZIP) [file pntd.0002839.s010.zip › Dataset S1/alignment-pictures/motifs-only/Motif_13.png]

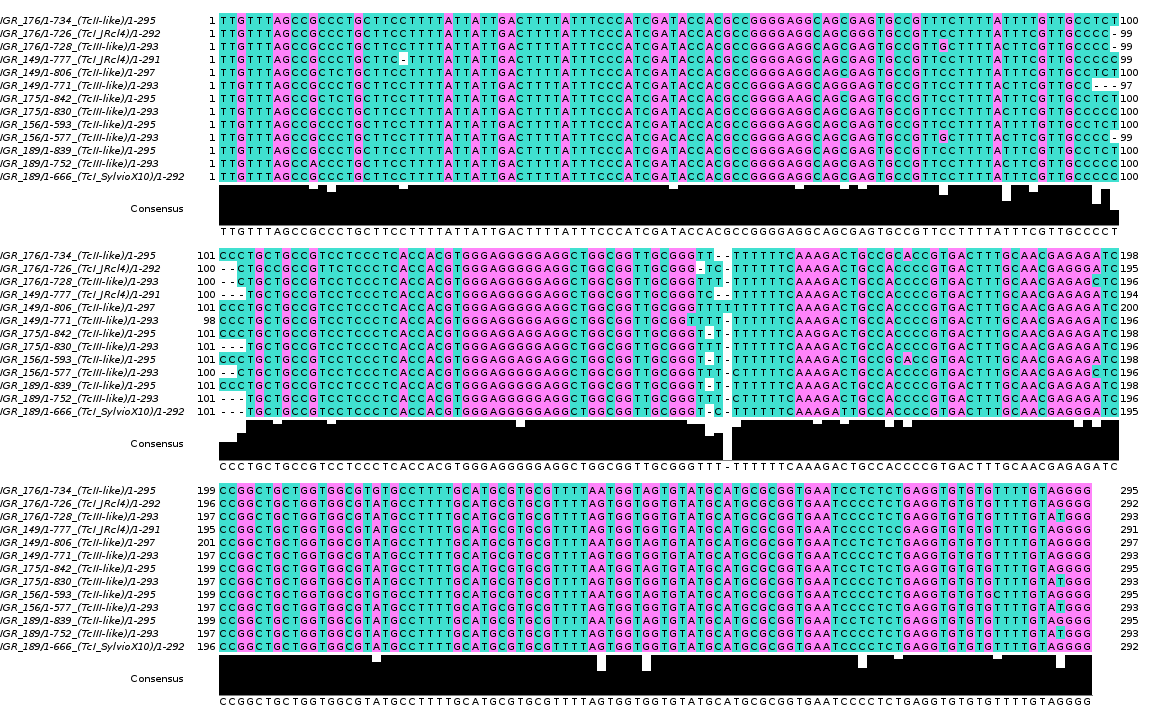

Supplement: Dataset S1 — Intergenic regions from unrelated loci that share blocks of significant sequence similarity. The file contains i) a spreadsheet summarizing listing the non-allelic, non-homologous IGR regions that share significant blocks of sequence similarity; ii) multiple sequence alignments where these unrelated IGRs were aligned to highlight the portion of the IGR that is shared; and iii) a figure in PDF format that shows the alignment in context. The alignments are provided in CLUSTAL format (.clw) and as colored renderings in PNG format, as produced by Jalview (purine/pyrimidine color scheme, only applied to regions of the alignment with 90% identity). The IGR IDs listed in each alignment correspond to those in Table S3. (ZIP) [file pntd.0002839.s010.zip › Dataset S1/alignment-pictures/motifs-only/Motif_6.png]

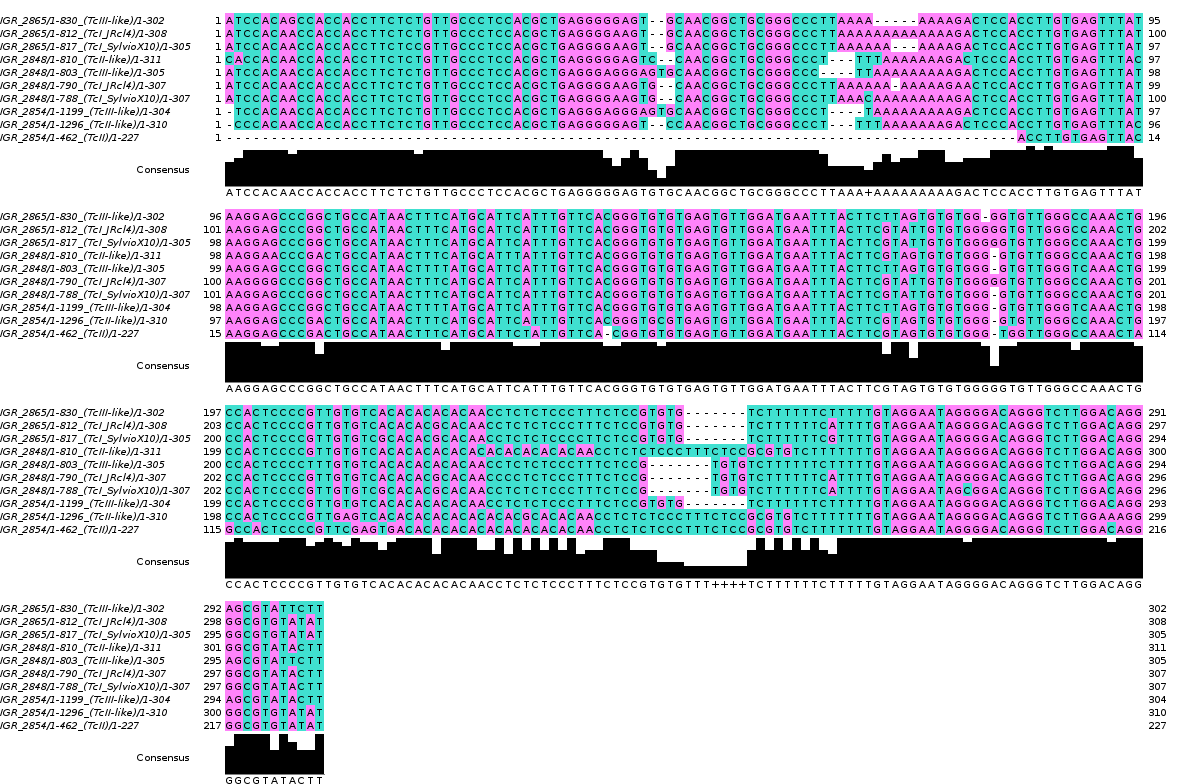

Supplement: Dataset S1 — Intergenic regions from unrelated loci that share blocks of significant sequence similarity. The file contains i) a spreadsheet summarizing listing the non-allelic, non-homologous IGR regions that share significant blocks of sequence similarity; ii) multiple sequence alignments where these unrelated IGRs were aligned to highlight the portion of the IGR that is shared; and iii) a figure in PDF format that shows the alignment in context. The alignments are provided in CLUSTAL format (.clw) and as colored renderings in PNG format, as produced by Jalview (purine/pyrimidine color scheme, only applied to regions of the alignment with 90% identity). The IGR IDs listed in each alignment correspond to those in Table S3. (ZIP) [file pntd.0002839.s010.zip › Dataset S1/alignment-pictures/motifs-only/Motif_5.png]

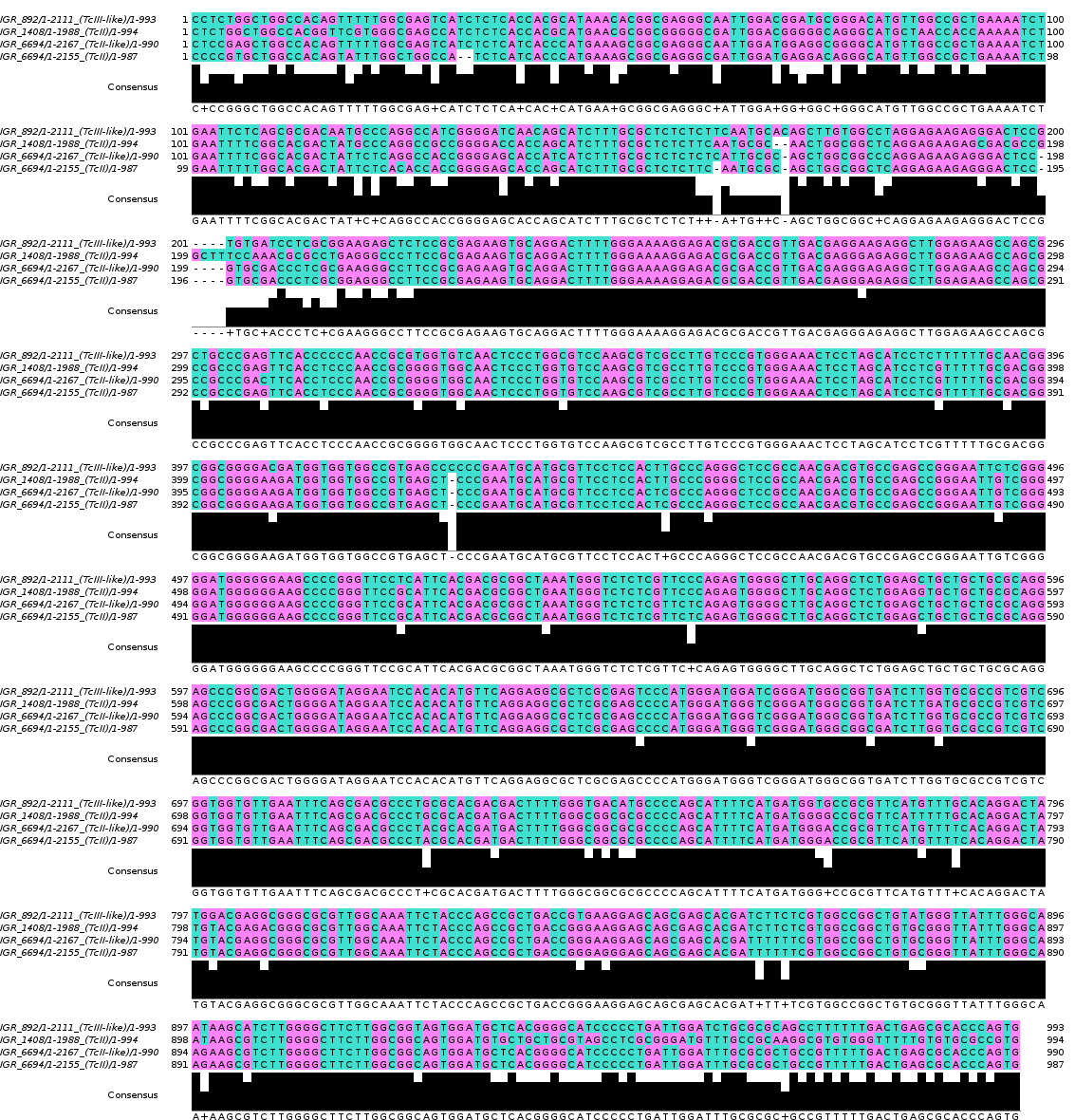

Supplement: Dataset S1 — Intergenic regions from unrelated loci that share blocks of significant sequence similarity. The file contains i) a spreadsheet summarizing listing the non-allelic, non-homologous IGR regions that share significant blocks of sequence similarity; ii) multiple sequence alignments where these unrelated IGRs were aligned to highlight the portion of the IGR that is shared; and iii) a figure in PDF format that shows the alignment in context. The alignments are provided in CLUSTAL format (.clw) and as colored renderings in PNG format, as produced by Jalview (purine/pyrimidine color scheme, only applied to regions of the alignment with 90% identity). The IGR IDs listed in each alignment correspond to those in Table S3. (ZIP) [file pntd.0002839.s010.zip › Dataset S1/alignment-pictures/motifs-only/Motif_3.png]
