# Supplementary material for: A Genome-Wide Analysis of Genetic Diversity in Trypanosoma cruzi Intergenic Regions
Source: PLoS Negl Trop Dis. 2014 May 1;8(5):e2839. doi: 10.1371/journal.pntd.0002839 (PMC4006747; doi:10.1371/journal.pntd.0002839)

Conserved sequence motif #1

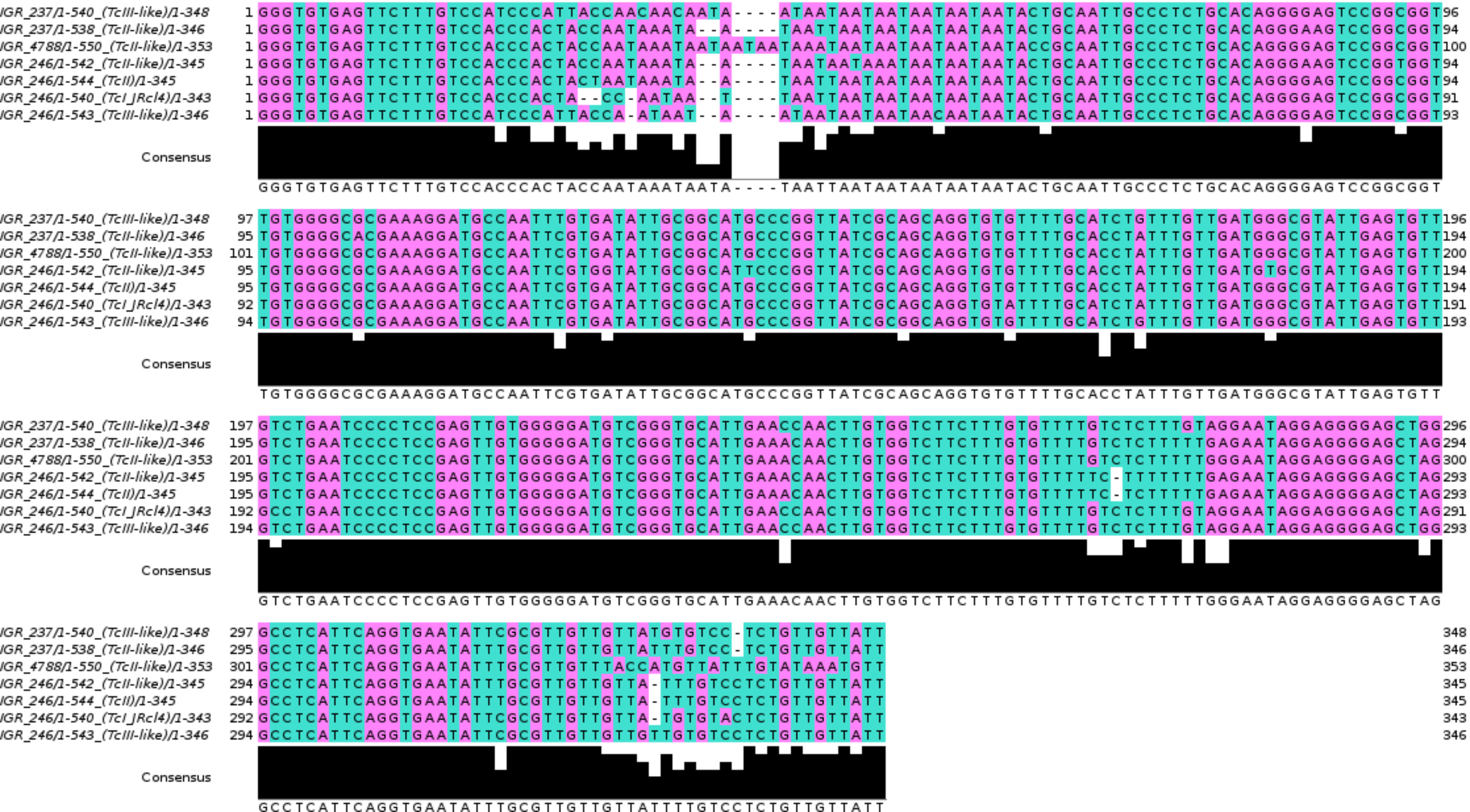

Conserved sequence motif #2

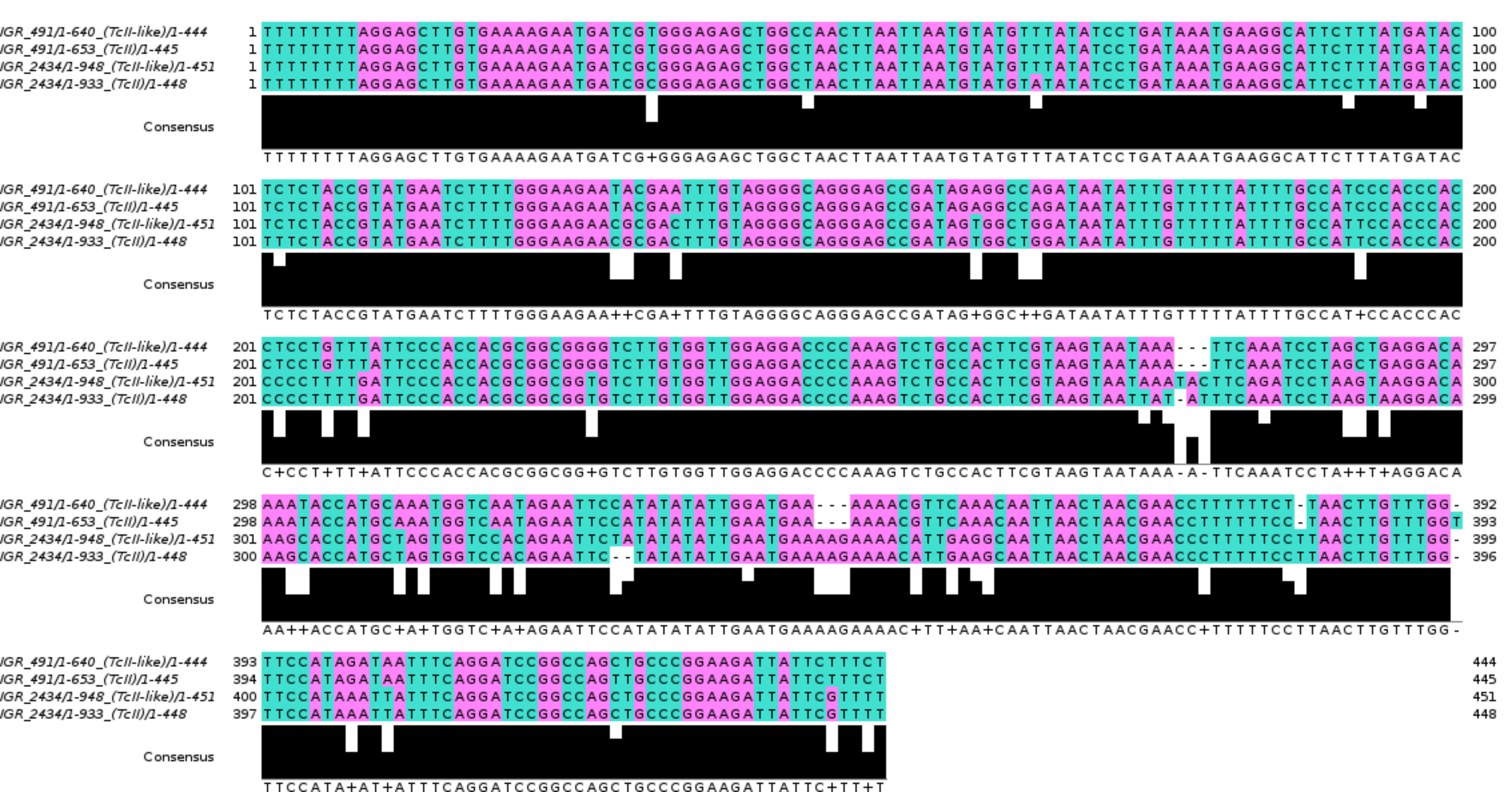

Conserved sequence motif #3

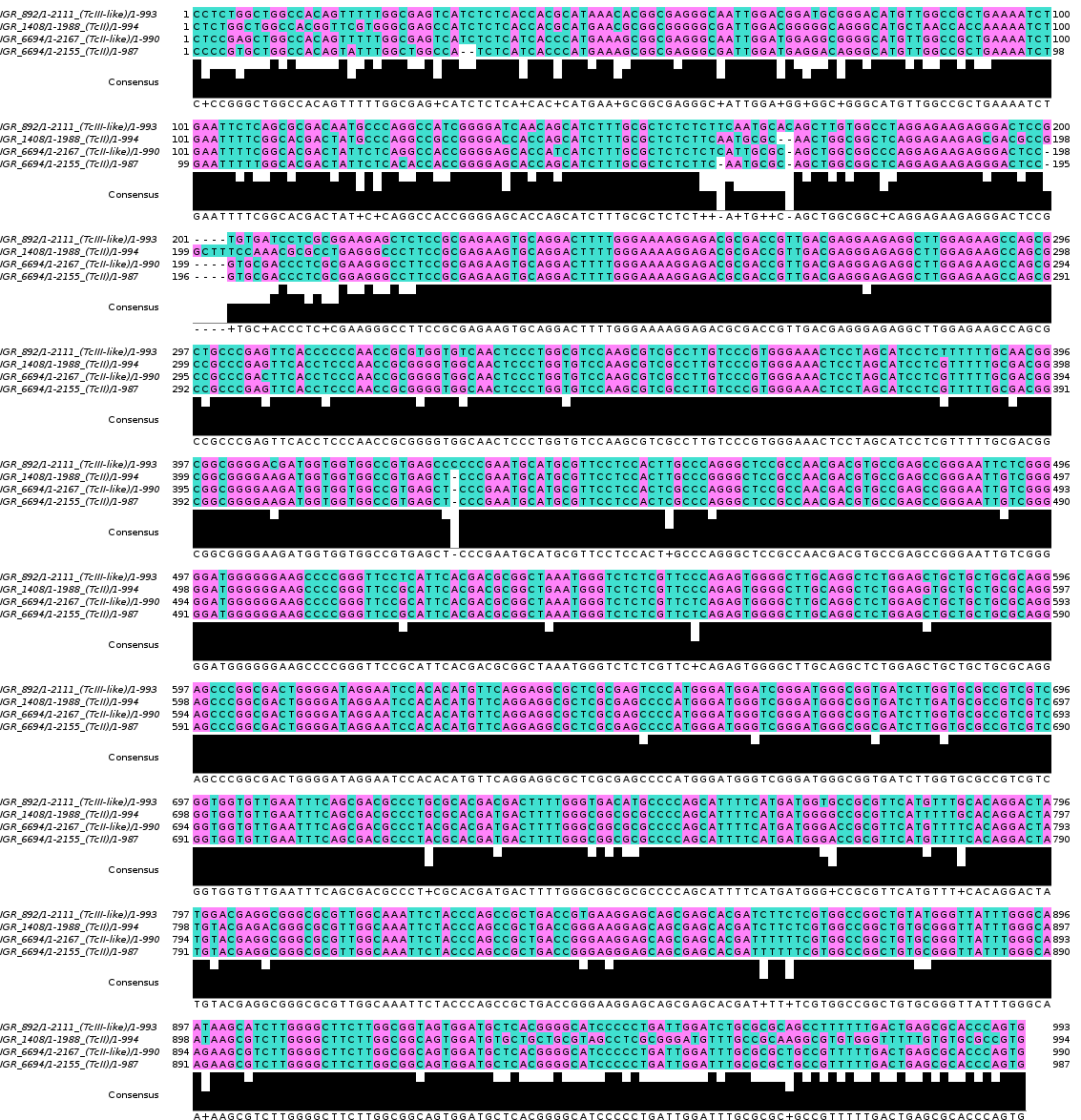

Conserved sequence motif #4

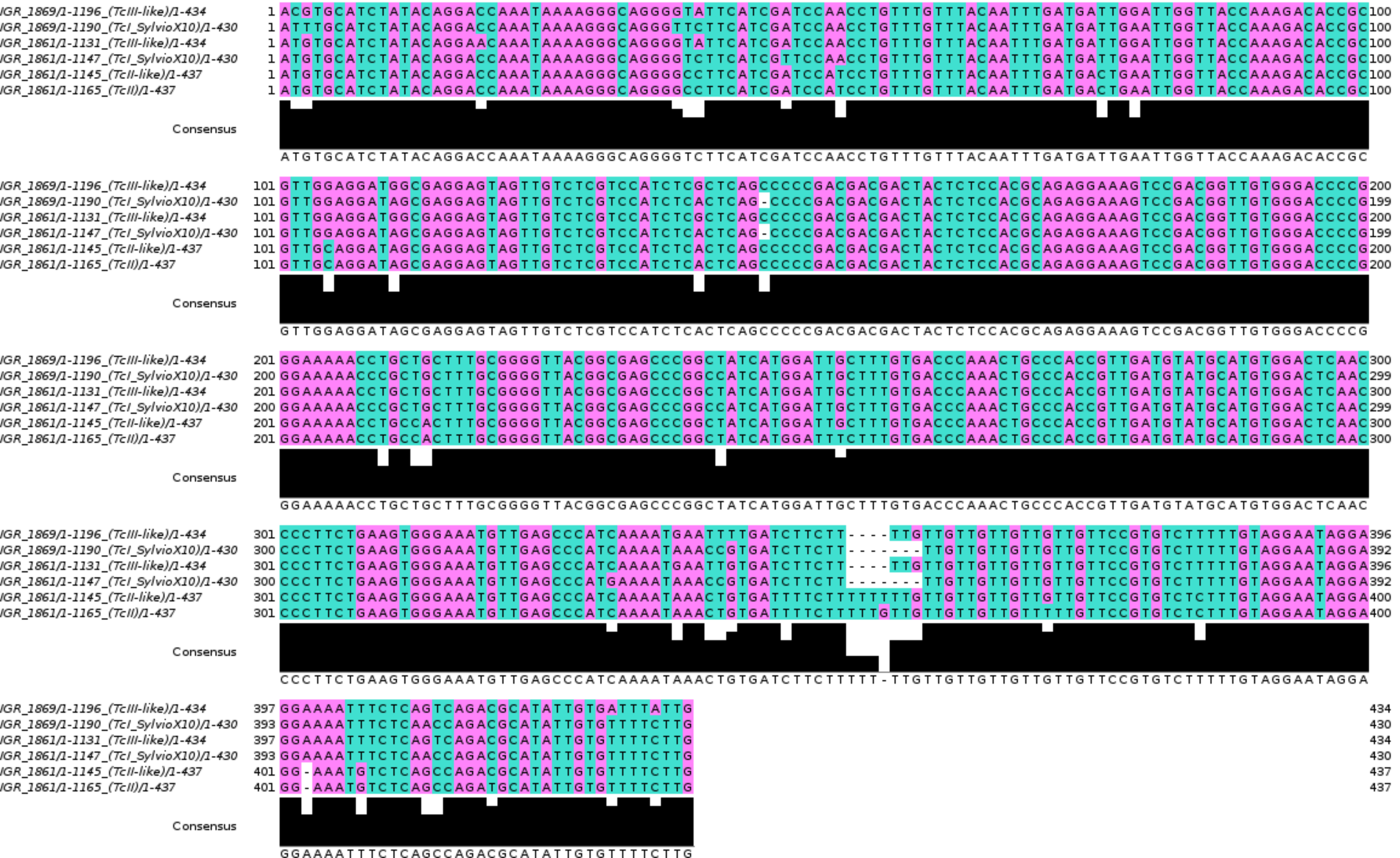

Conserved sequence motif #5

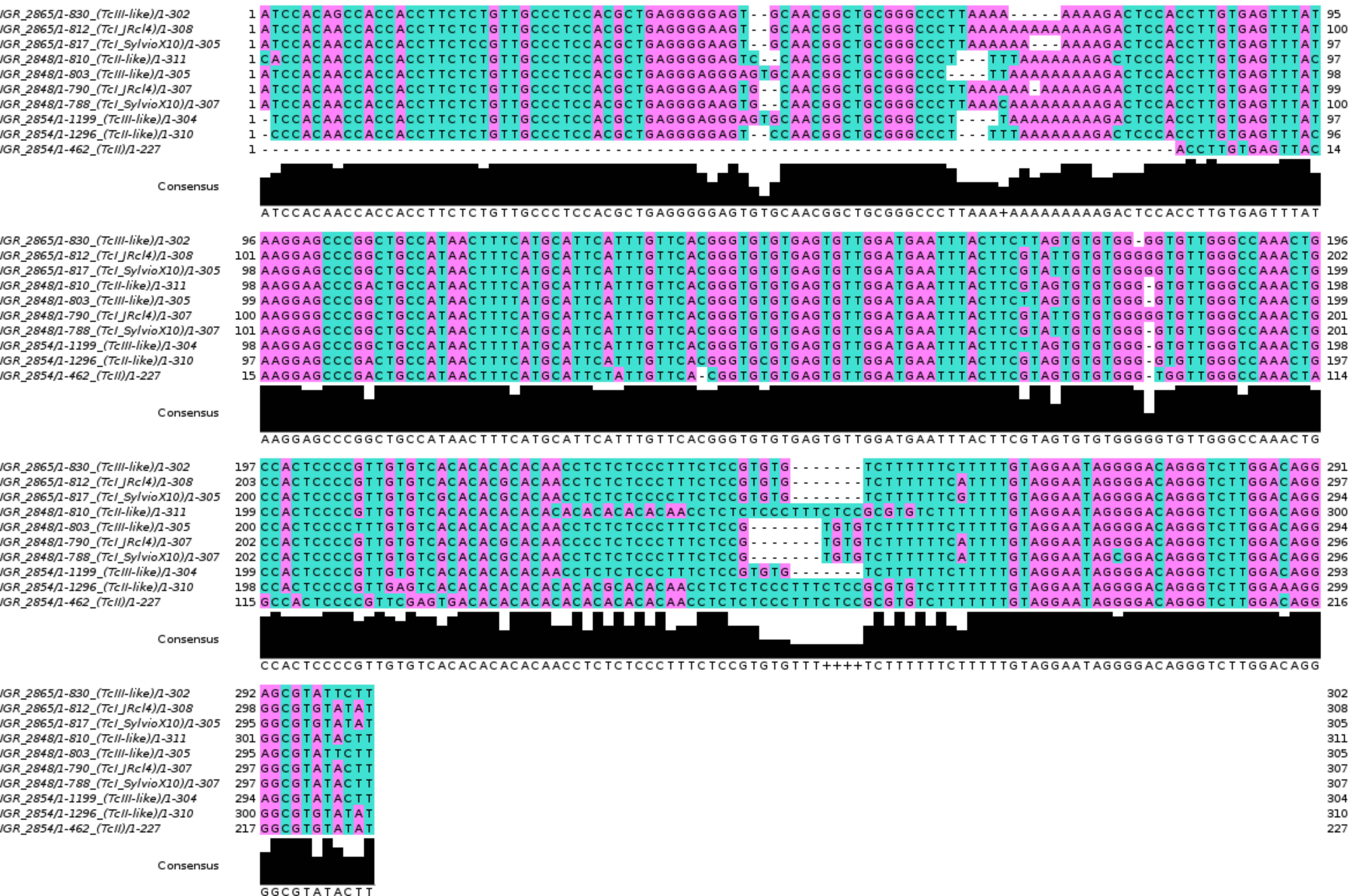

Conserved sequence motif #6

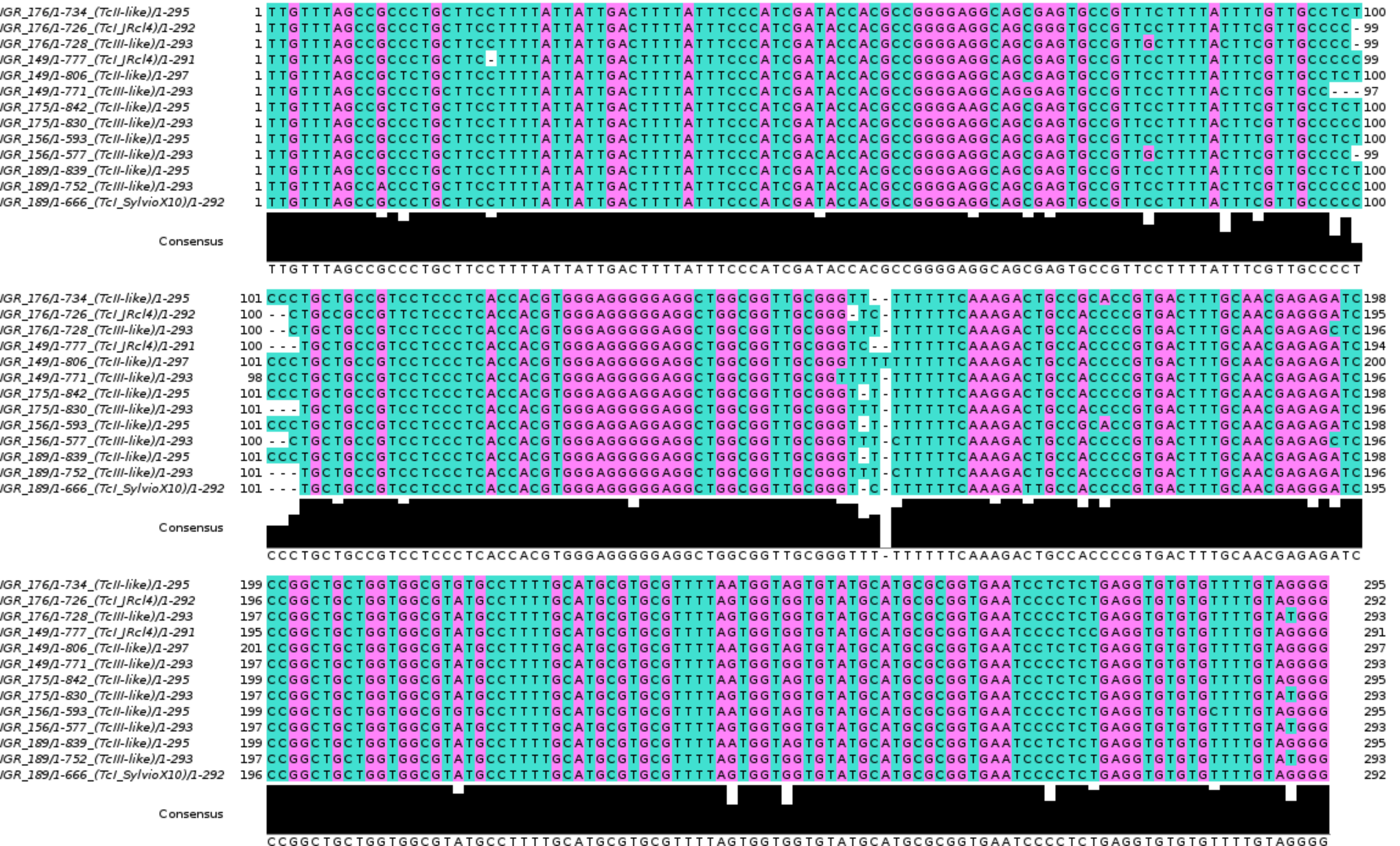

Conserved sequence motif #7

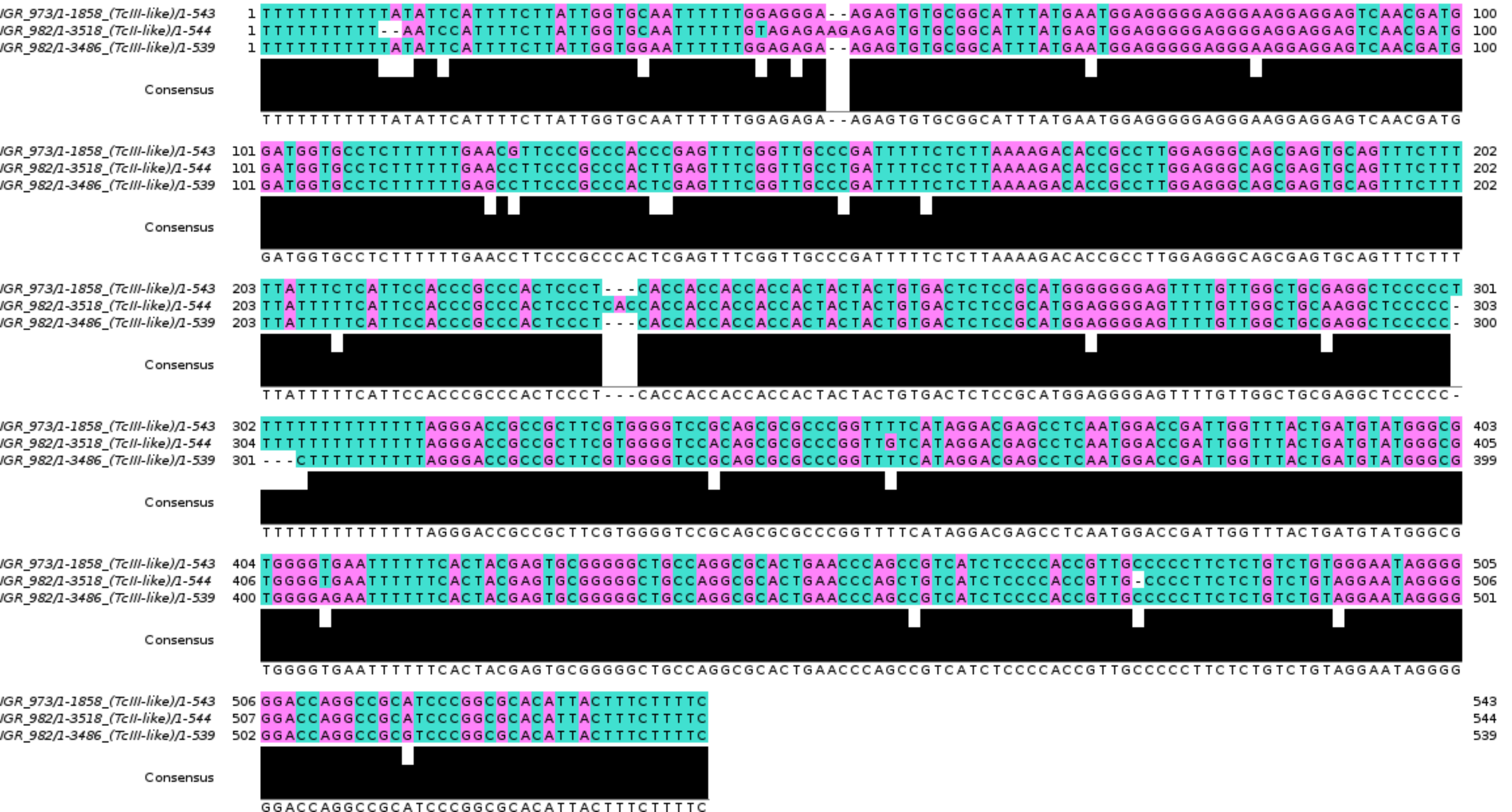

Conserved sequence motif #8

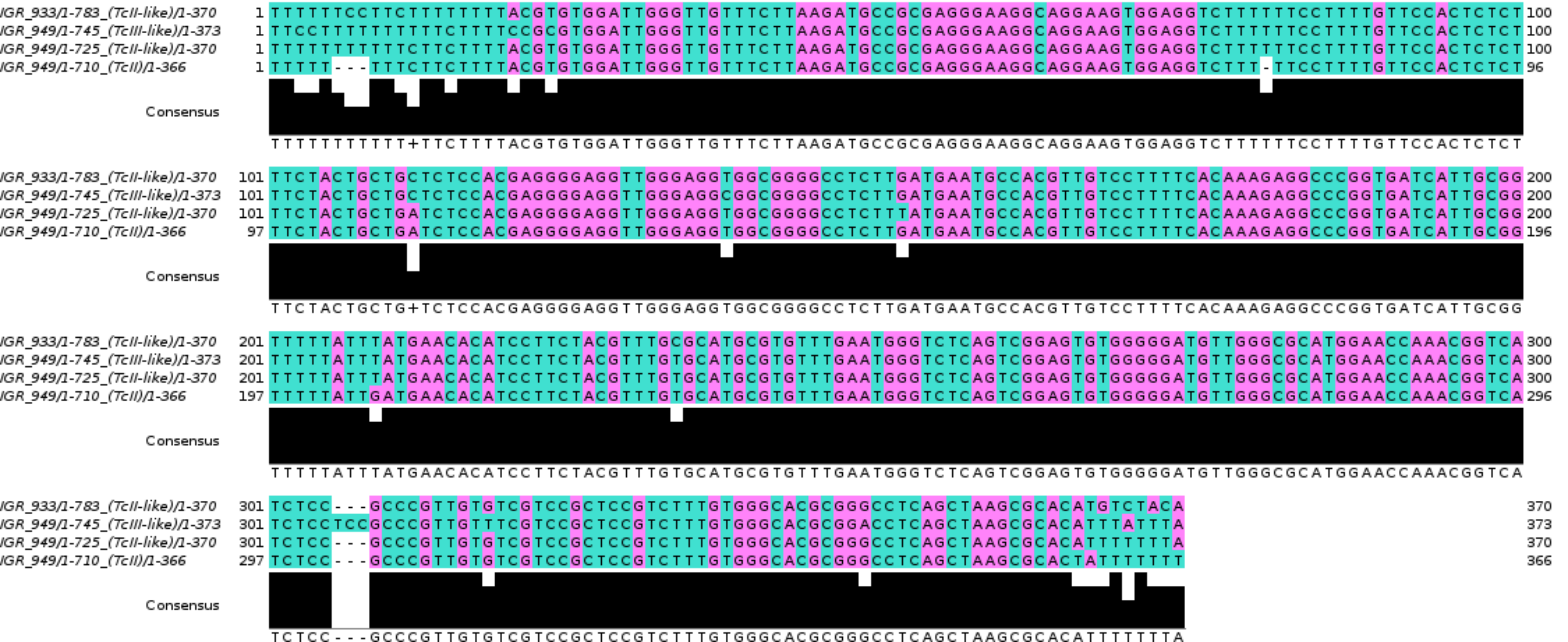

Conserved sequence motif #9

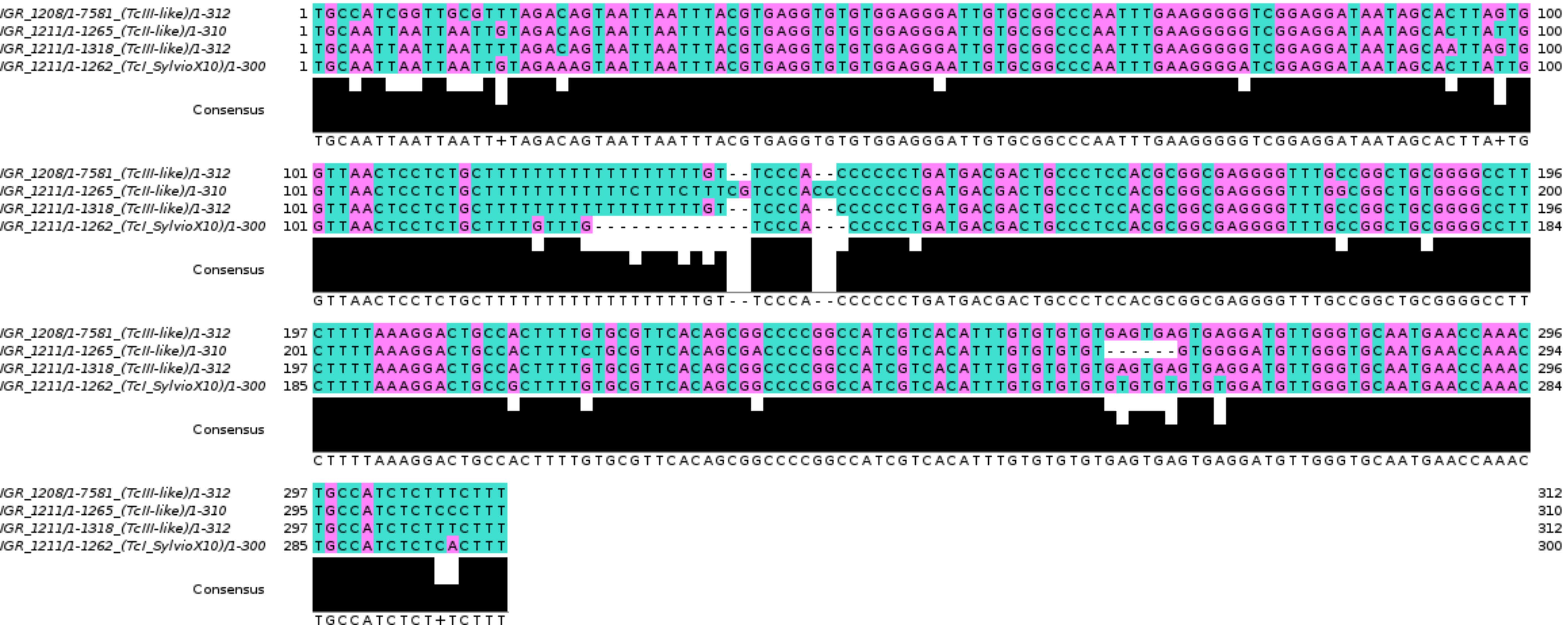

Conserved sequence motif #10

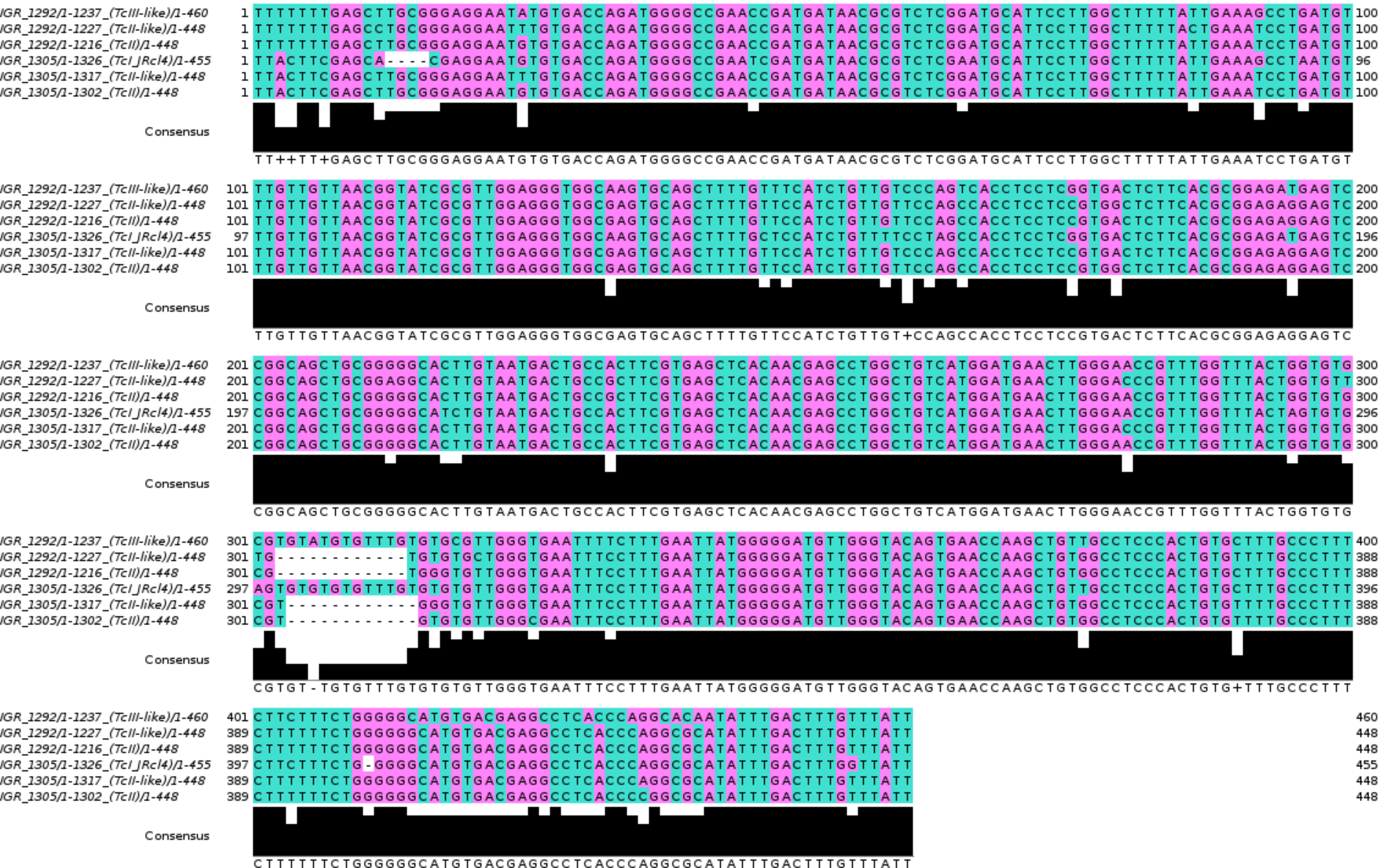

Conserved sequence motif #11

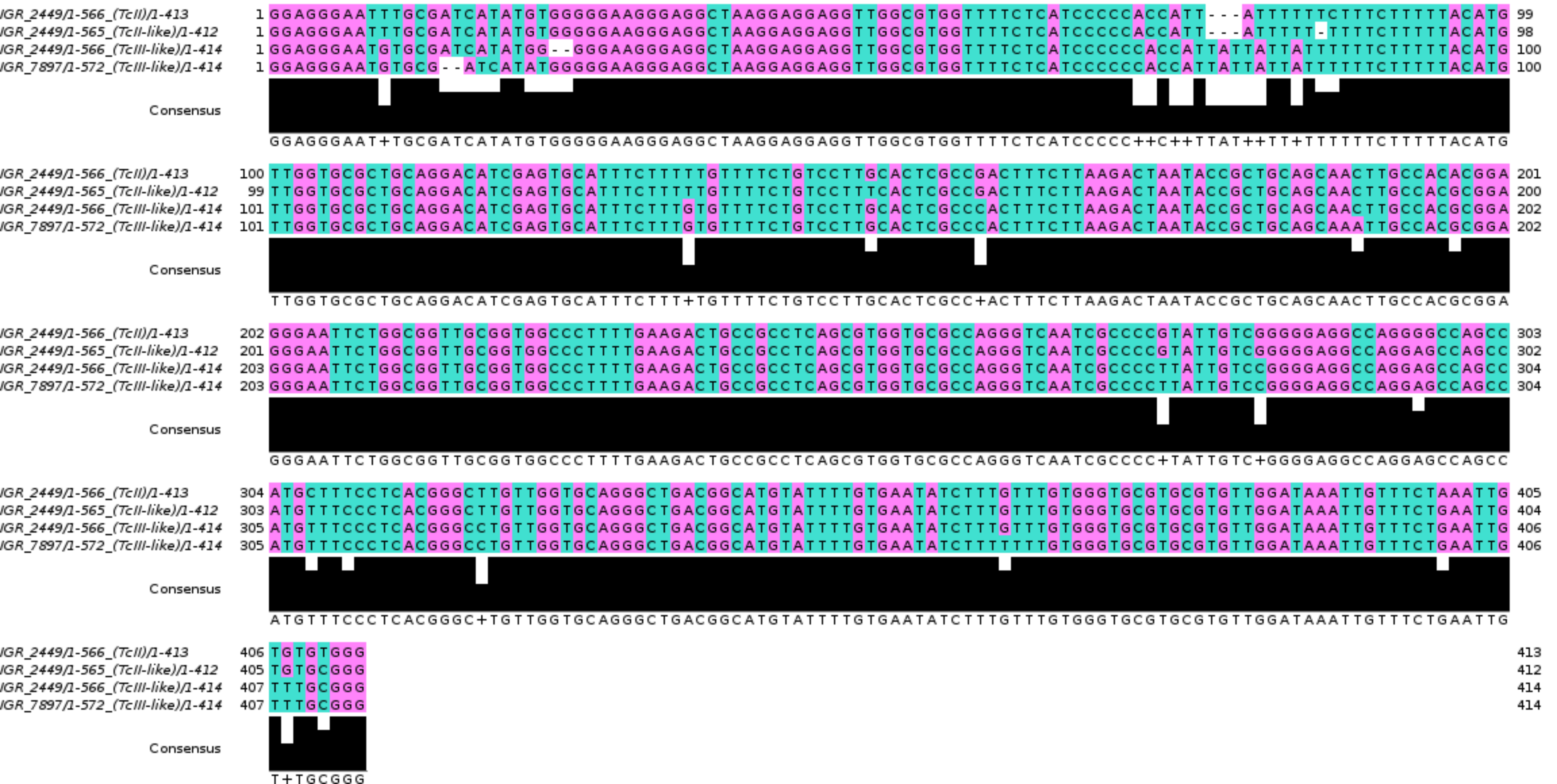

Conserved sequence motif #12

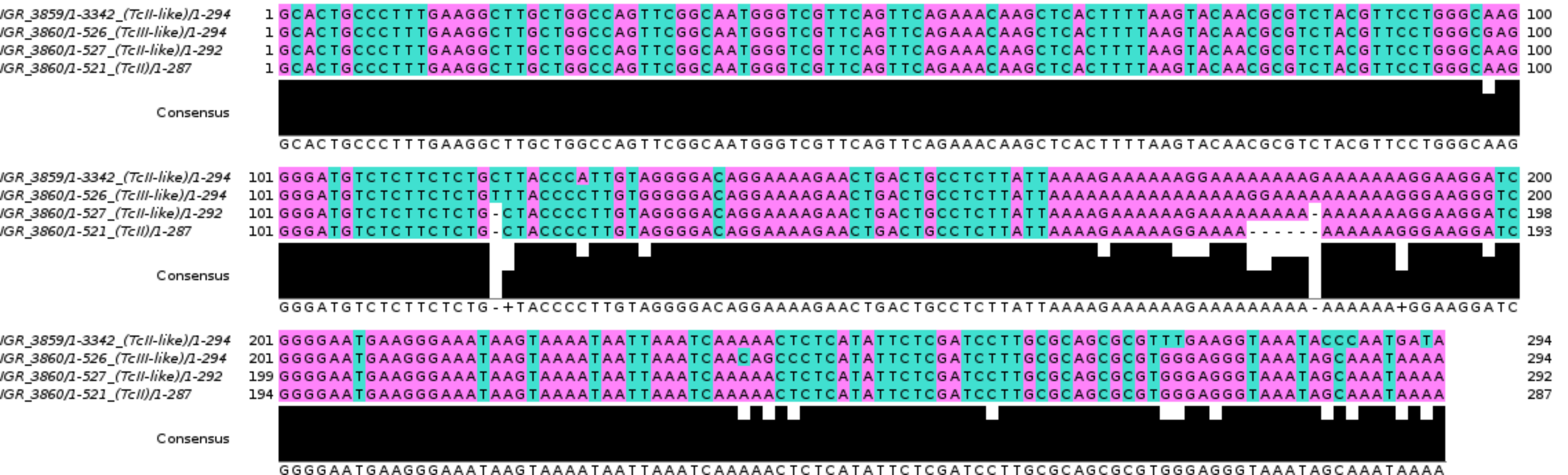

Conserved sequence motif #13

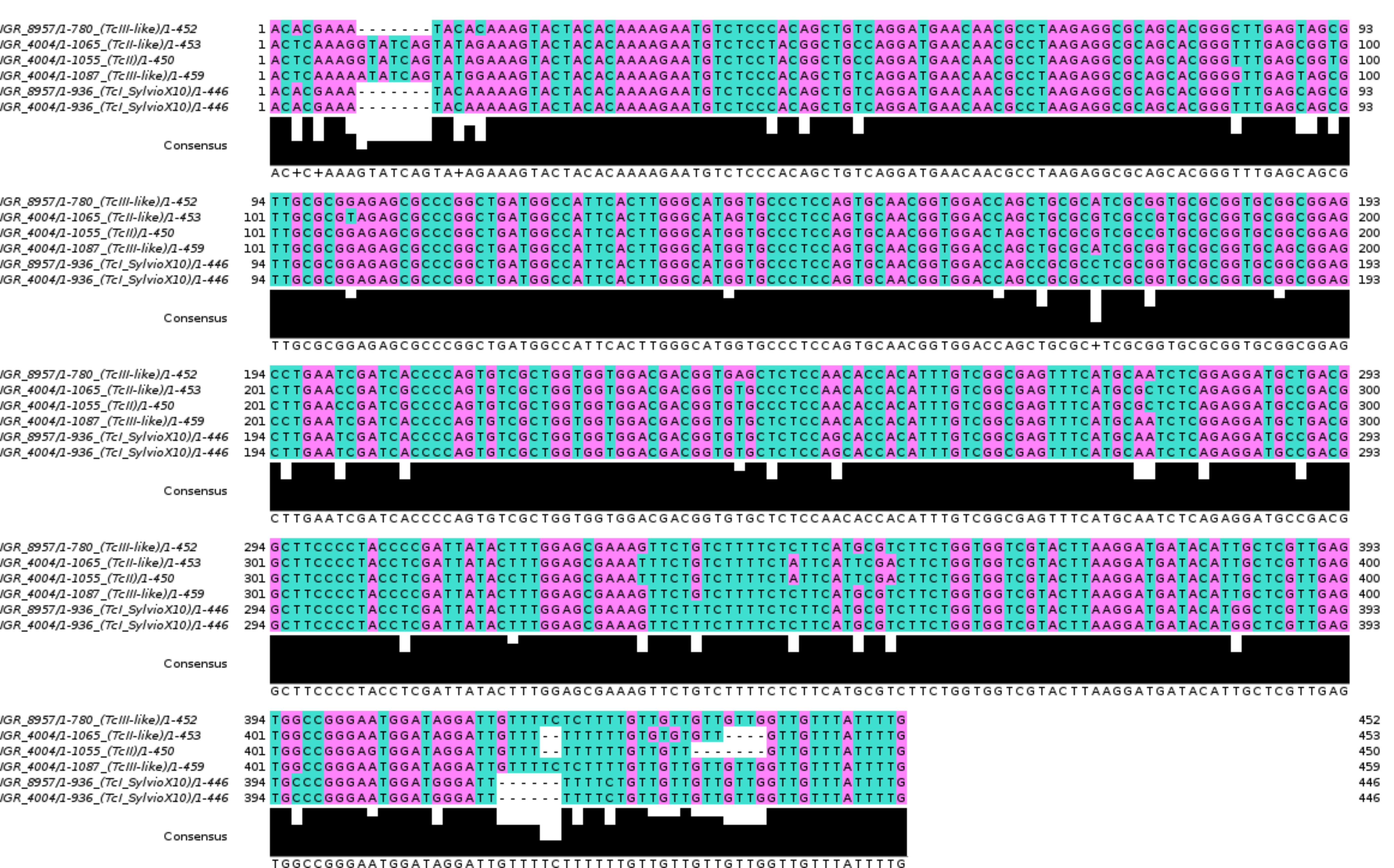

Conserved sequence motif #14

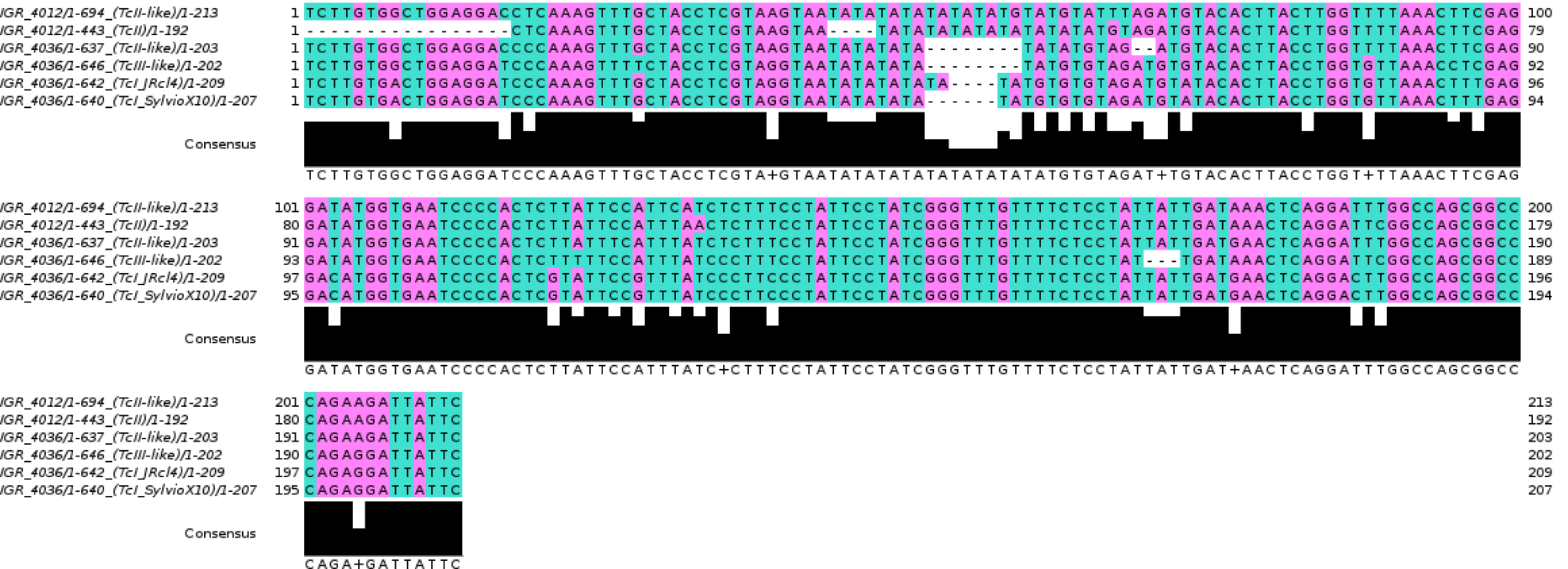

Conserved sequence motif #15

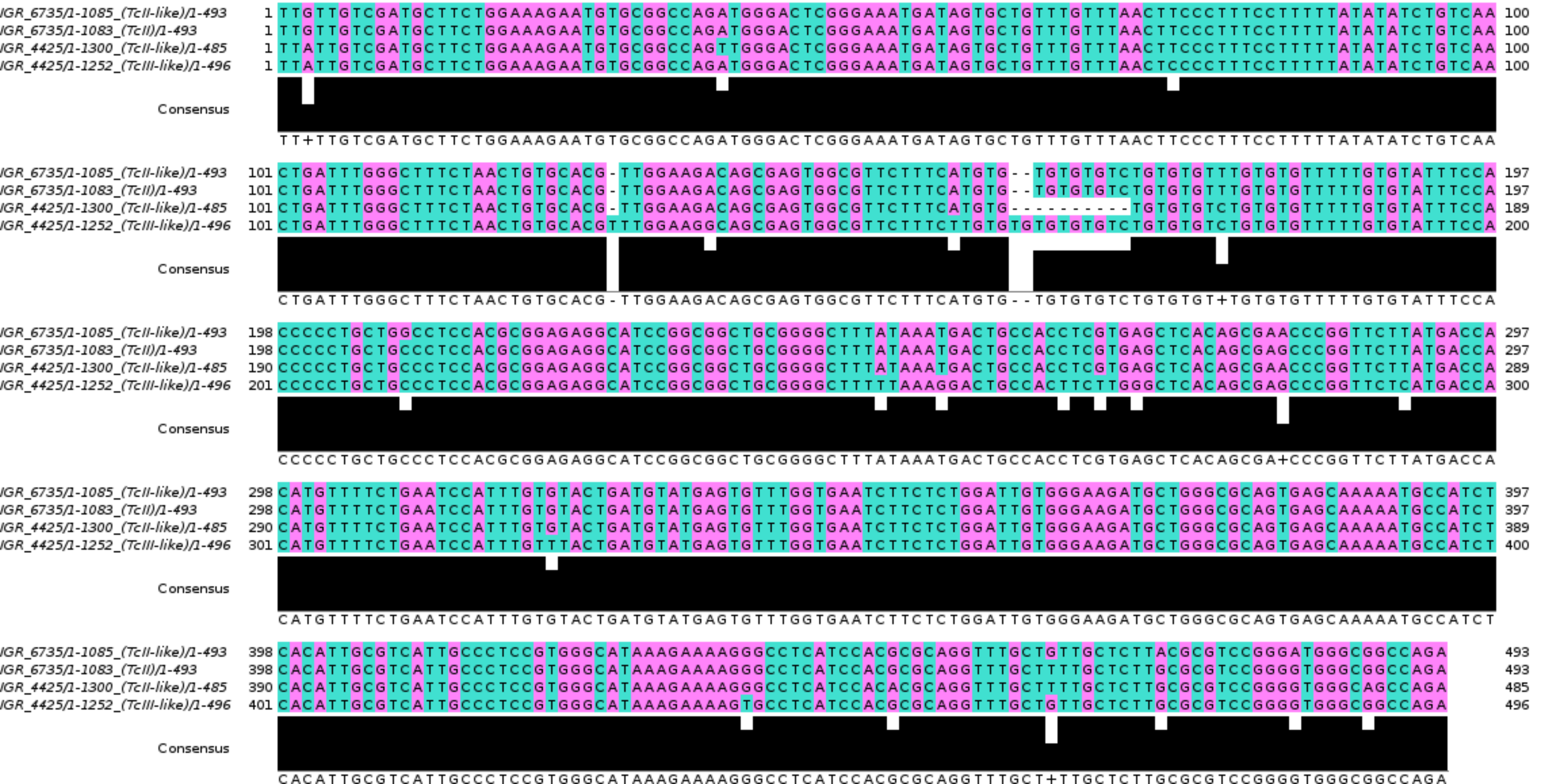

Conserved sequence motif #16

```
IGR_6481/1-1432_(TcII-like)/1-476     1 TTGA---CATTTCGATTTTGTGAAAAGAATGATTGCGAGAGGGCTGGCCACCCAATTTAATATGCACATCCTGATAAGACGAAAATATTTATTTACAATT 97
IGR_6481/1-1424_(TcII)/1-479          1 TTGA---CATTTCGATTTTGTGAAAAGAATGATTGCGAGAGGGCTAGCCACCCAATTTAATATGCACATCCTGATAAGACGAAAATATTTATTTACAATT 97
IGR_6514/1-1422_(TcII-like)/1-480     1 TTGATTGTGTTGGGAGCTTGTGAAAAGAATGATTGCGAGAGGGCTGGCCACCCGATTTAATATGCACATCCTGATAAGACGAAAACATTTATTTACAATT 100
IGR_6514/1-1455_(TcII)/1-479          1 TTGATTGTGTTAGGAGCTTGTGAAAAGAATGATTGCGAGAGGGCTGGCCACCCAATTTAATATGCACATCCTGATAAGACGAAAATATTTATTTACAATT 100
Consensus                               TTGATTG++TTT+GA++TTGTGAAAAGAATGATTGCGAGAGGGCTGGCCACCCAATTTAATATGCACATCCTGATAAGACGAAAATATTTATTTACAATT

IGR_6481/1-1432_(TcII-like)/1-476    98 ATTTTCTCTCTTACGGTTCATTTGTAAGACTGCAATGGGAGGAGTCAATGAACCGATAAAGTCACGACGGATTTGTTT---GTTTTTATTTTTGTCGTTC 194
IGR_6481/1-1424_(TcII)/1-479         98 ATTTTCTCTCTTACGGTTCATTTGTAAGACCGCAATGGGAGGAGTCAATGAACCGATAACATCAAGACGGATTTGTTTTTGTTTTTTATTTTTGTCGTTC 197
IGR_6514/1-1422_(TcII-like)/1-480   101 ATTTTCTCTCTTACGGTTCATTTGTAAGACCGCAATGGGAGGAGTCAATGAACCGATAAAGTCACGACGGATTTGTTCT--TTTTTTATTTTTGTCGTTC 198
IGR_6514/1-1455_(TcII)/1-479        101 ATTTTCTCTCTTACGGTTCATTTGTAAGACCGCAATGGGAGGAGTCAATGAACCGATAACGTCAAGACGGATTTGTTT---TTTTTTATTTTTGTCGTTC 197
Consensus                               ATTTTCTCTCTTACGGTTCATTTGTAAGACCGCAATGGGAGGAGTCAATGAACCGATAA+GTCA+GACGGATTTGTTTT--TTTTTTATTTTTGTCGTTC

IGR_6481/1-1432_(TcII-like)/1-476   195 CACTCGGCCCCTTTTGATTCCCCCCACCTTGCGGCGGTGTCTTGTGGCAGGAGGACCTCAAATTCTACCAATTTTTAACTAATATAAACATCCCCAATCC 294
IGR_6481/1-1424_(TcII)/1-479        198 CACTCGGCCCCTTTTGATTCCCCCCACCTTGCGGCGGTGTCTTGTGGCAGGAGGACCTCAAATTCTACCAATTTTTAACTAATATAAACATCCCCAATCC 297
IGR_6514/1-1422_(TcII-like)/1-480   199 CACTCGGCCCCTTTTGATTCCTCCCACCTTGCGGCGGTGTCTTGTGGCAGGAGGACCTTAAATTCCACCAATTTTTAACTAATATAAACATCCCCAATCC 298
IGR_6514/1-1455_(TcII)/1-479        198 CACTCGGCCCCTTTTGATTCCCCCCACCTTGCGGCGGTGTCTTGTGGCTGGAGGACCTCAAATTCTACCAATTTTTAACTAATATAAACATCCCCAATCC 297
Consensus                               CACTCGGCCCCTTTTGATTCCCCCCACCTTGCGGCGGTGTCTTGTGGCAGGAGGACCTCAAATTCTACCAATTTTTAACTAATATAAACATCCCCAATCC

IGR_6481/1-1432_(TcII-like)/1-476   295 GAATAGAGCGCAACATGTCACGAGAGCGGCAATTTGAATAAATCTATCTATCTATATATATACTTGAAGCAAACATGGAGGATATGGCGAGGCCACCGCC 394
IGR_6481/1-1424_(TcII)/1-479        298 GAATAGAGCGCAACATGTCACGAGAGCGGCAATTTGAATAAATCTATCTATCTATATATATACTTGAAGCAAACATGGAGGATATGGCGAGGCCACCGCC 397
IGR_6514/1-1422_(TcII-like)/1-480   299 GAATAGAGCGCAACATGTCACGAGAGCGGCAATTTGAATAAATCTATCTATCTATATATATACTTGAAGCAAACATGGAGGATATGGCGAGGCCACCGCC 398
IGR_6514/1-1455_(TcII)/1-479        298 GAATAGAGCGCAACATGTCACGAGAGCGGCAATTTGAATAAATCTATCTATCTATATATATACTTGAAGCAAACATGGAGGATATGGCGAGGCCACCGCC 397
Consensus                               GAATAGAGCGCAACATGTCACGAGAGCGGCAATTTGAATAAATCTATCTATCTATATATATACTTGAAGCAAACATGGAGGATATGGCGAGGCCACCGCC

IGR_6481/1-1432_(TcII-like)/1-476   395 TCTCCTTTACCTTTTCTTTCTTGTCCTATTTTTATTTCTTTAGAGAGGTTCGGGATCTGGCTAGCAGCAGAGGTGAGTATTT 476
IGR_6481/1-1424_(TcII)/1-479        398 TCTCCTTTACCTTTTCTTTCTTGTCCTATTTTTATTTCTTTAGAGAGGTTCGGGATCTGGCTAGCAGCAGAGGTGAGTATTT 479
IGR_6514/1-1422_(TcII-like)/1-480   399 TCTCCTTTACCTTTTCTTTCTTGTCCTATTTTTATTTCTTTAGAGAGGTTCAGGATCTGGCTAGCAGCCCAGGCGGATGTTT 480
IGR_6514/1-1455_(TcII)/1-479        398 TCTCCTTTACCTTTTCTTTCTTGTCCTATTTTTATTTCTTTAGAGAGGTTCGGGATCTGGCTAGCAGCCCAGGCGGATGTTT 479
Consensus                               TCTCCTTTACCTTTTCTTTCTTGTCCTATTTTTATTTCTTTAGAGAGGTTCGGGATCTGGCTAGCAGC++AGG+G++T+TTT
```

Conserved sequence motif #17

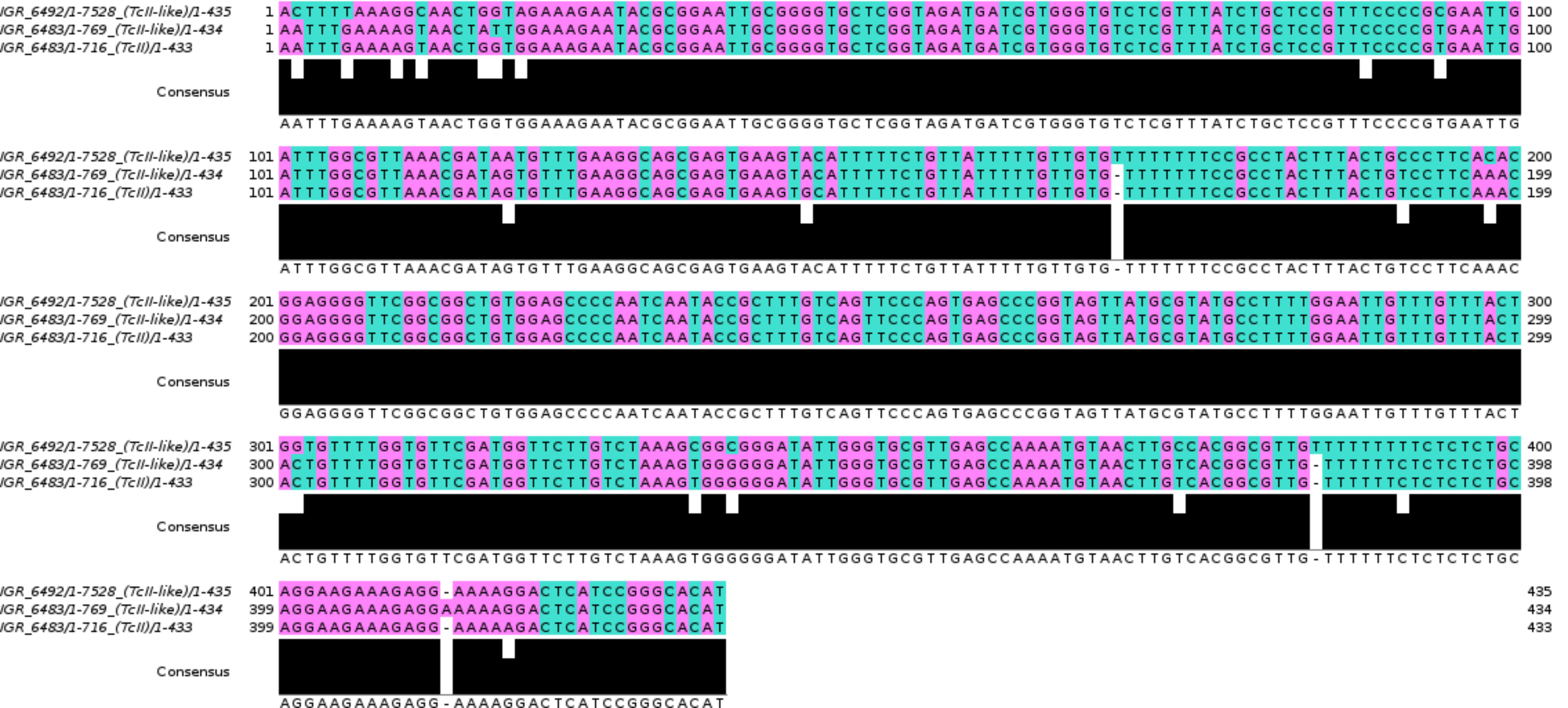

Conserved sequence motif #18

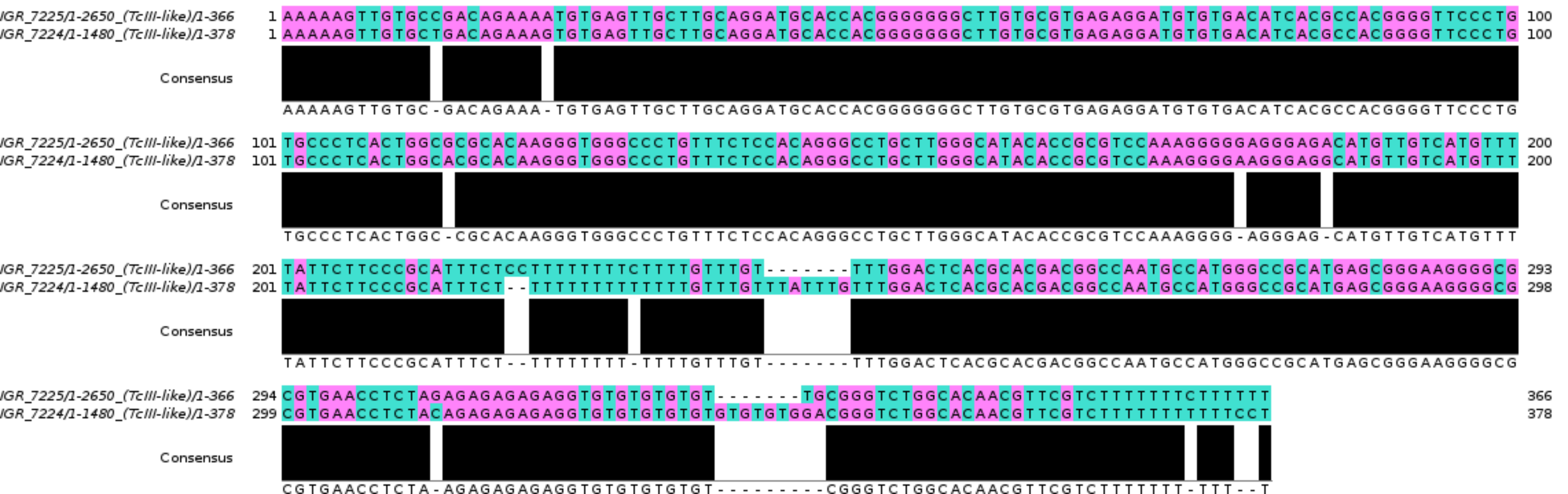

Conserved sequence motif #19

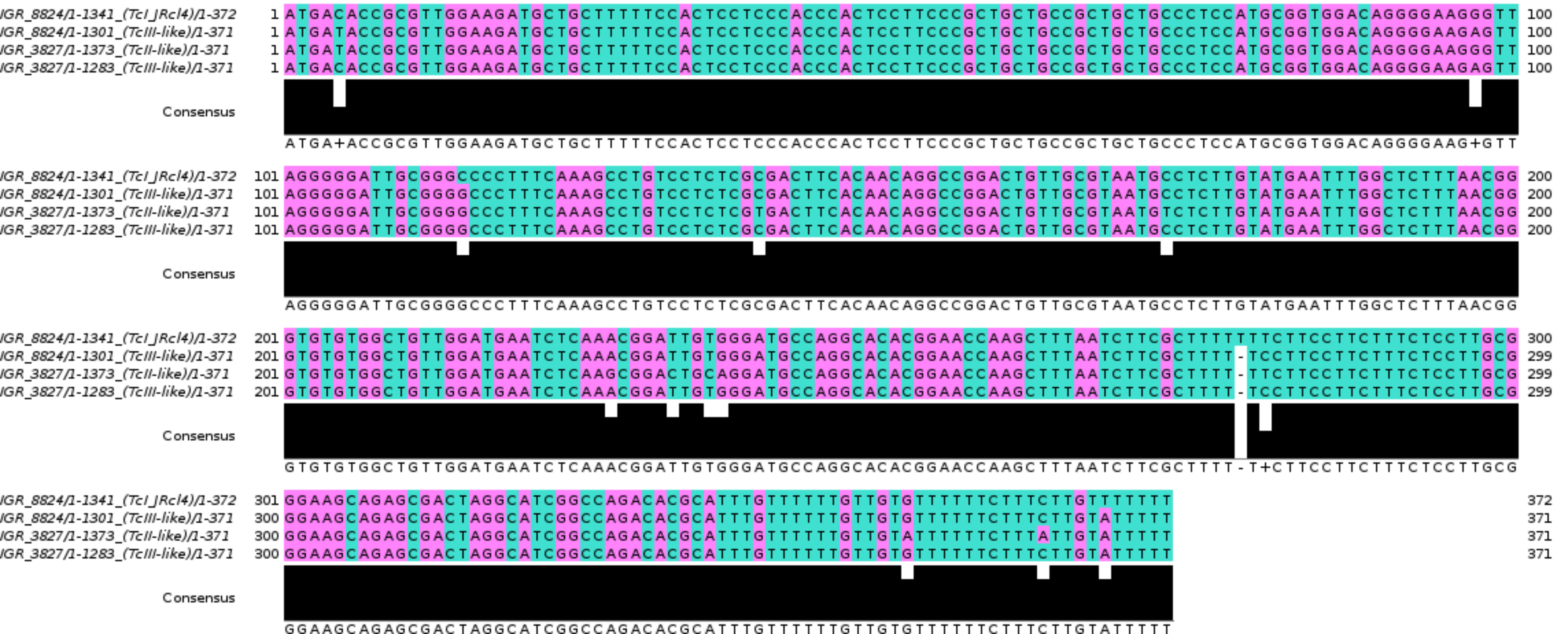

Conserved sequence motif #20

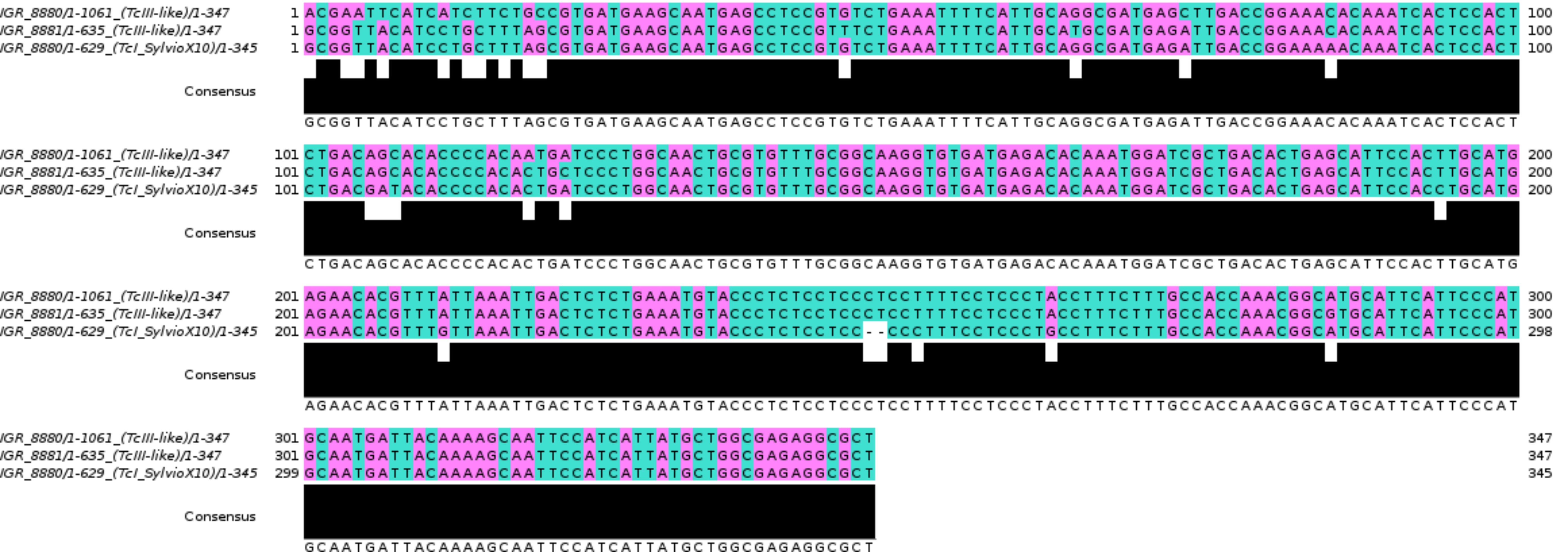

Conserved sequence motif #21

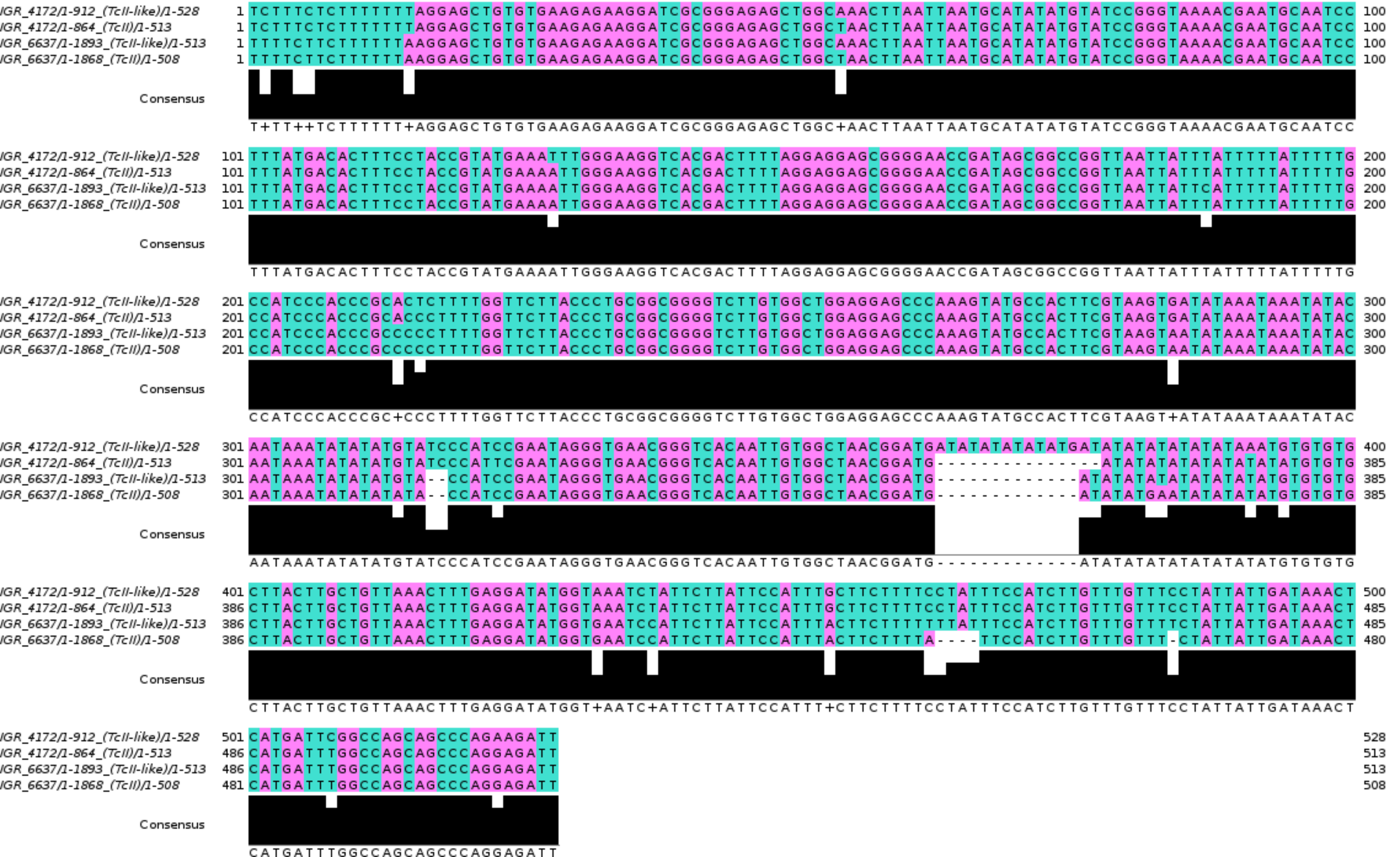

Supplement: Dataset S1 — Intergenic regions from unrelated loci that share blocks of significant sequence similarity. The file contains i) a spreadsheet summarizing listing the non-allelic, non-homologous IGR regions that share significant blocks of sequence similarity; ii) multiple sequence alignments where these unrelated IGRs were aligned to highlight the portion of the IGR that is shared; and iii) a figure in PDF format that shows the alignment in context. The alignments are provided in CLUSTAL format (.clw) and as colored renderings in PNG format, as produced by Jalview (purine/pyrimidine color scheme, only applied to regions of the alignment with 90% identity). The IGR IDs listed in each alignment correspond to those in Table S3. (ZIP) [file pntd.0002839.s010.zip › Dataset S1/Figure-D1b.pdf]
