# Supplementary material for: A Genome-Wide Analysis of Genetic Diversity in Trypanosoma cruzi Intergenic Regions
Source: PLoS Negl Trop Dis. 2014 May 1;8(5):e2839. doi: 10.1371/journal.pntd.0002839 (PMC4006747; doi:10.1371/journal.pntd.0002839)

Motif #1

Motif #	IGR ID	CDS A				CDS B			
		TcII-like		TcIII-like		TcII-like		TcIII-like	
		CDS ID	Product Description	CDS ID	Product Description	CDS ID	Product Description	CDS ID	Product Description
1	237	TcCLB.508277.20	protein kinase, putative	TcCLB.509941.120	protein kinase, putative	TcCLB.508277.30	hypothetical protein, conserved	TcCLB.509941.140	hypothetical protein, conserved
	246	TcCLB.508277.140	phosphomevalonate kinase protein, putative	TcCLB.507913.20	phosphomevalonate kinase protein, putative	TcCLB.508277.150	proliferative cell nuclear antigen (PCNA), putative	TcCLB.507913.30	proliferative cell nuclear antigen (PCNA), putative
	4788	TcCLB.508277.220	hypothetical protein	TcCLB.509945.30	hypothetical protein	TcCLB.508277.230	nucleolar RNA binding protein, putative	No_ortholog	-

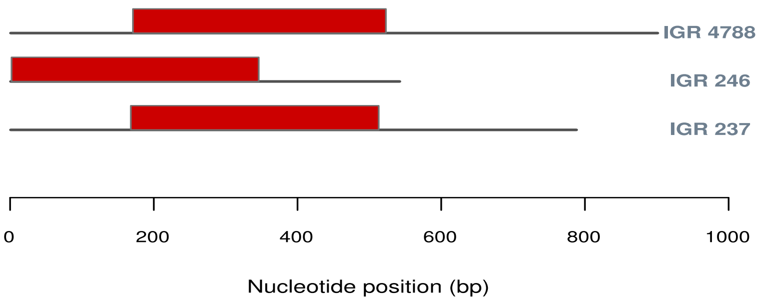

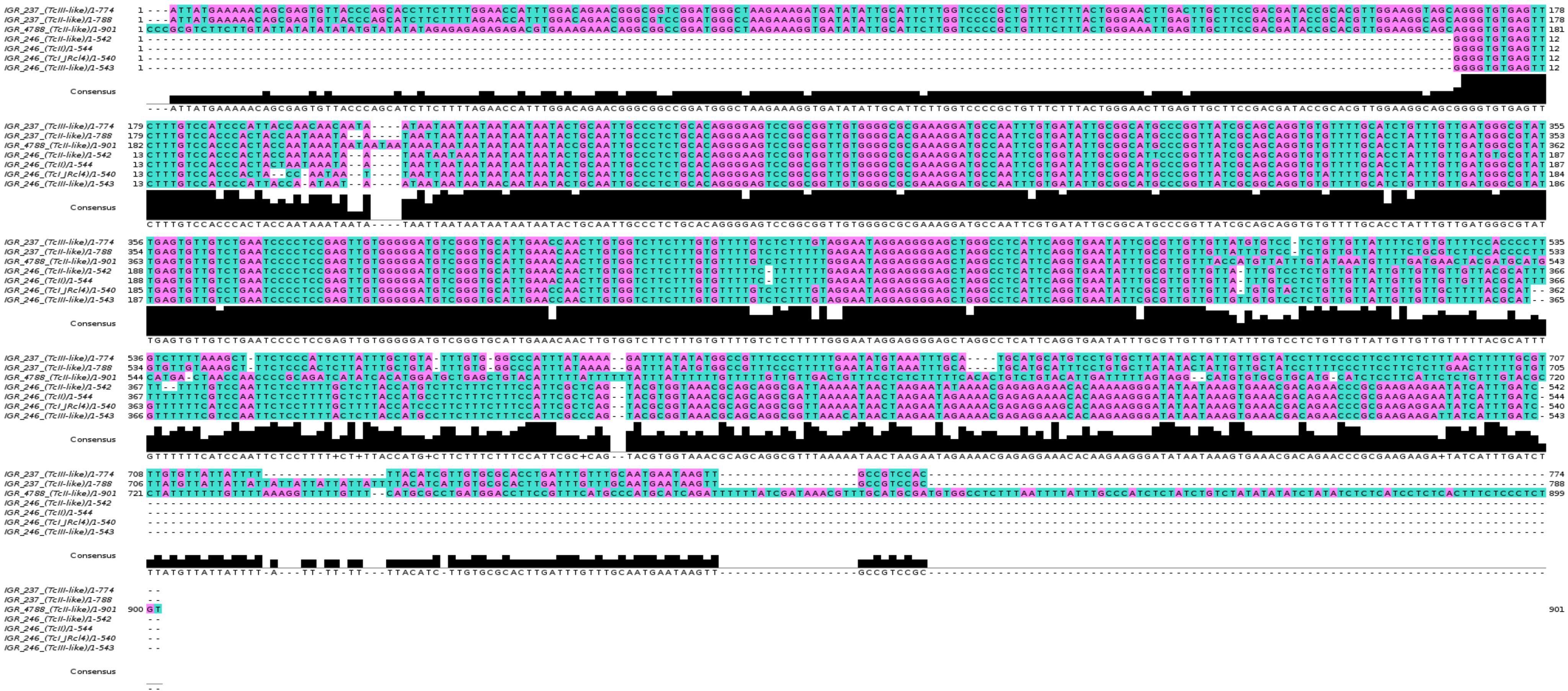

Motif #2

Motif #	IGR ID	CDS A				CDS B			
		TcII-like		TcIII-like		TcII-like		TcIII-like	
		CDS ID	Product Description	CDS ID	Product Description	CDS ID	Product Description	CDS ID	Product Description
2	491	TcCLB.508781.70	palmitoyl acyltransferase 3, putative	TcCLB.506855.10	palmitoyl acyltransferase 3, putative	TcCLB.508781.80	hypothetical protein, conserved	TcCLB.506853.70	hypothetical protein, conserved
	2434	TcCLB.506931.10	haloacid dehalogenase-like hydrolase, putative	TcCLB.510131.40	haloacid dehalogenase-like hydrolase, putative	TcCLB.506931.4	hypothetical protein, conserved	TcCLB.510131.44	hypothetical protein, conserved

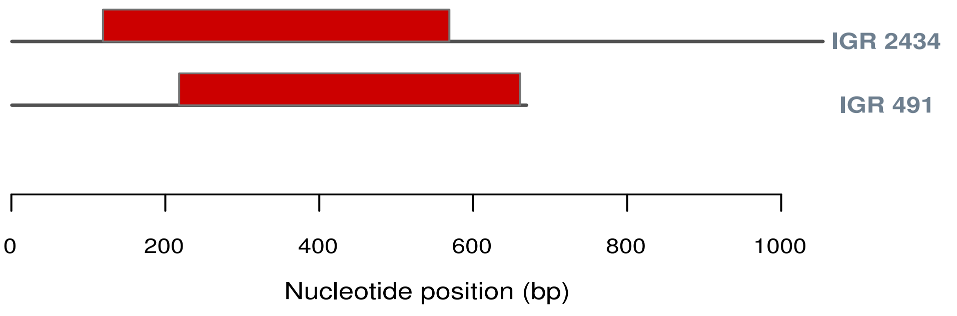

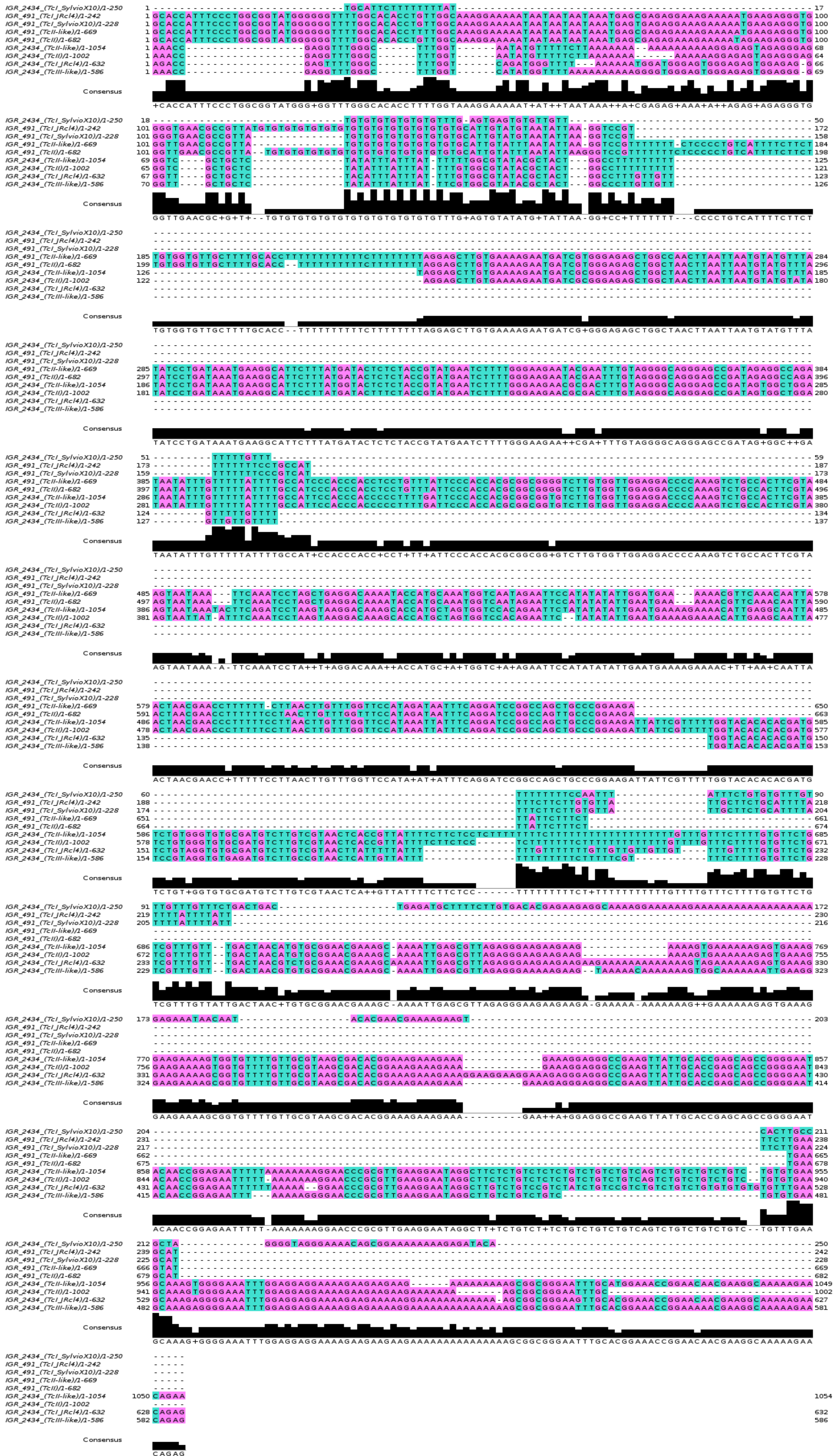

Motif #3

Motif #	IGR ID	CDS A				CDS B			
		TcII-like		TcIII-like		TcII-like		TcIII-like	
		CDS ID	Product Description	CDS ID	Product Description	CDS ID	Product Description	CDS ID	Product Description
3	892	TcCLB.509233.50	hypothetical protein, conserved	TcCLB.509437.70	hypothetical protein, conserved	TcCLB.509233.60	hypothetical protein, conserved	TcCLB.509437.60	hypothetical protein, conserved
	1408	TcCLB.510257.80	peptidase M20/M25/M40, putative	TcCLB.509213.120	peptidase M20/M25/M40, putative	TcCLB.510257.90	autophagocytosis associated protein, putative	TcCLB.509213.130	autophagocytosis associated protein, putative
	6694	TcCLB.510761.44	cyclophilin-like protein, putative	No_ortholog	-	TcCLB.510761.60	casein kinase II, putative	No_ortholog	-

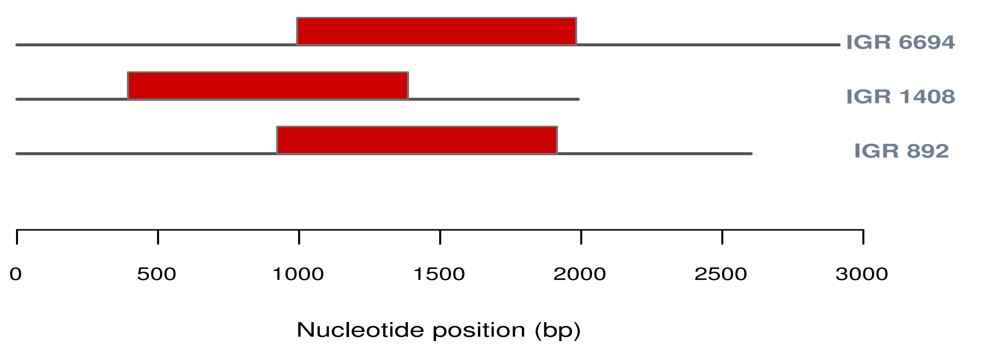

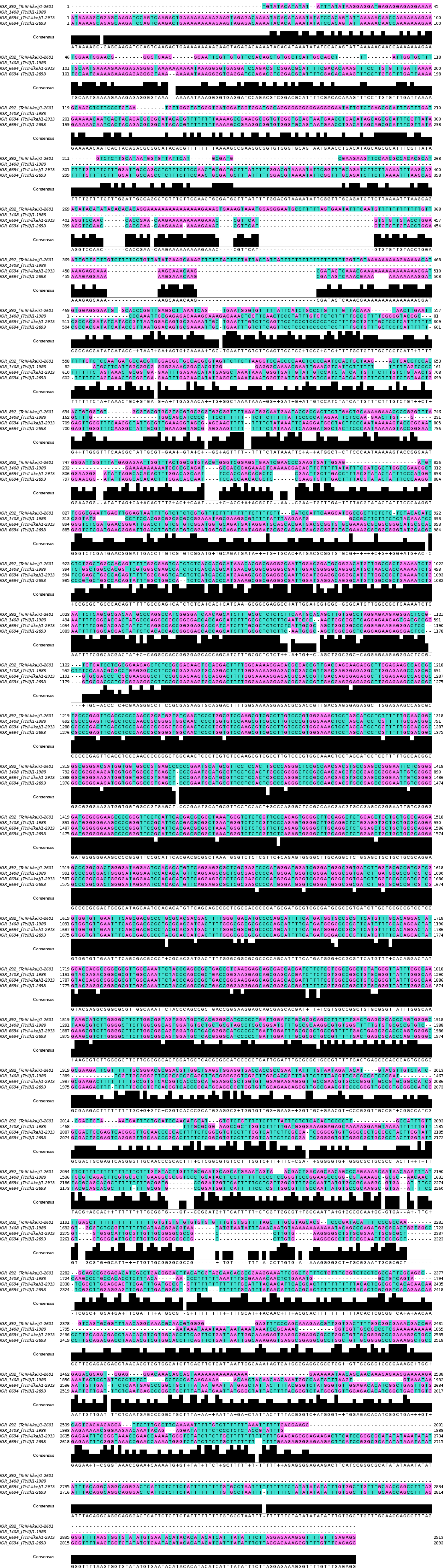

Motif #4

Motif #	IGR ID	CDS A				CDS B			
		TcII-like		TcIII-like		TcII-like		TcIII-like	
		CDS ID	Product Description	CDS ID	Product Description	CDS ID	Product Description	CDS ID	Product Description
4	1861	TcCLB.511071.120	hypothetical protein, conserved	TcCLB.511901.20	hypothetical protein, conserved	TcCLB.511071.100	hypothetical protein, conserved	TcCLB.511901.30	hypothetical protein, conserved
	1869	TcCLB.511067.30	transketolase, putative	TcCLB.511903.120	transketolase, putative	TcCLB.511067.20	60S ribosomal protein L26, putative	TcCLB.511903.130	60S ribosomal protein L26, putative

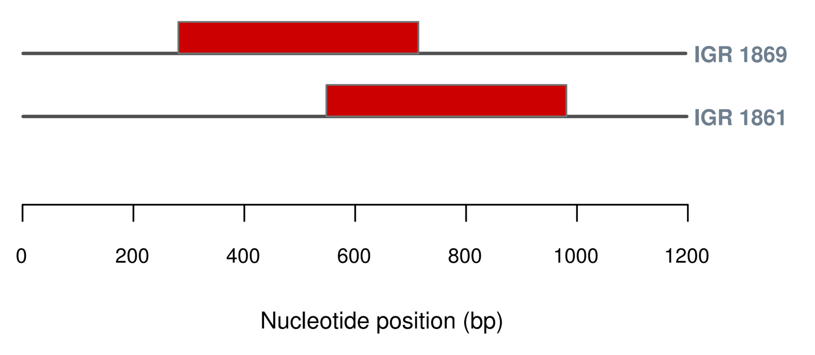

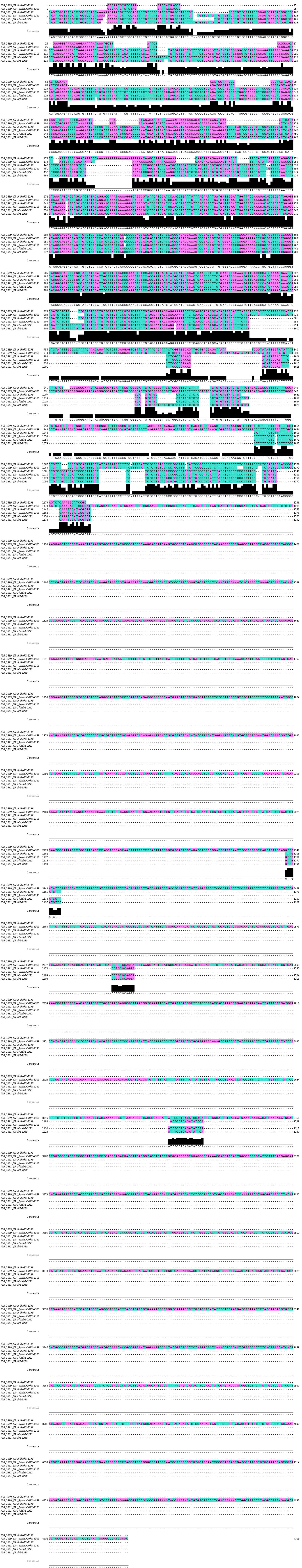

Motif #5

Motif #	IGR ID	CDS A				CDS B			
		TcII-like		TcIII-like		TcII-like		TcIII-like	
		CDS ID	Product Description	CDS ID	Product Description	CDS ID	Product Description	CDS ID	Product Description
5	2848	TcCLB.511585.180	hypothetical protein, conserved	TcCLB.510155.170	hypothetical protein, conserved	TcCLB.511585.170	protein kinase, putative	TcCLB.510155.160	protein kinase, putative
	2854	TcCLB.511585.125	hypothetical protein, conserved	TcCLB.510155.115	hypothetical protein, conserved	TcCLB.511585.120	hypothetical protein, conserved	TcCLB.510155.110	hypothetical protein, conserved
	2865	TcCLB.511585.20	KREL1	TcCLB.510155.20	KREL1	TcCLB.511585.10	hypothetical protein, conserved	TcCLB.510155.10	hypothetical protein, conserved

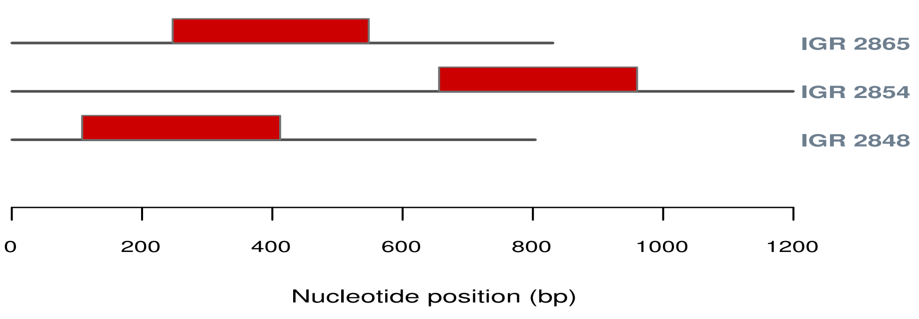

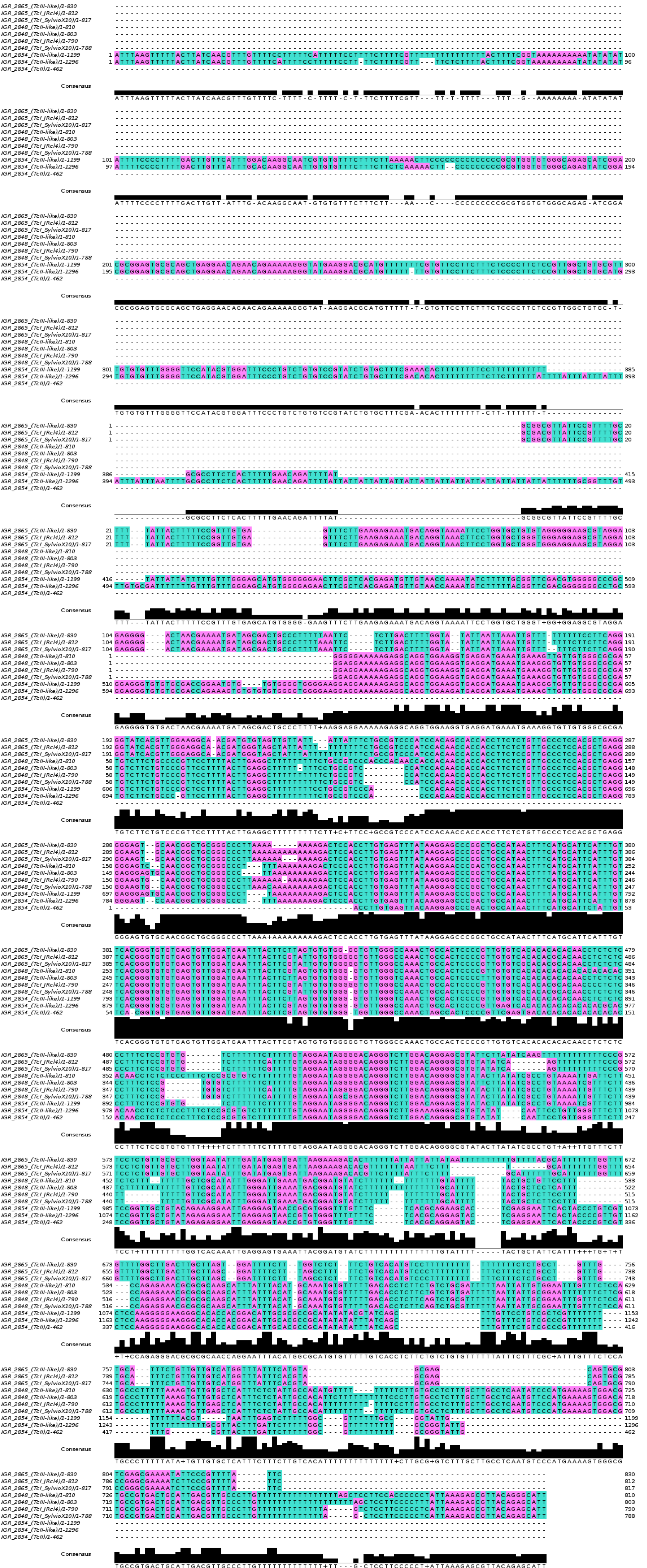

Motif #6

Motif #	IGR ID	CDS A				CDS B			
		TcII-like		TcIII-like		TcII-like		TcIII-like	
		CDS ID	Product Description	CDS ID	Product Description	CDS ID	Product Description	CDS ID	Product Description
6	149	TcCLB.506529.70	hypothetical protein, conserved	TcCLB.508593.130	hypothetical protein, conserved	TcCLB.506529.80	hypothetical protein, conserved	TcCLB.508593.140	hypothetical protein, conserved
	156	TcCLB.506529.140	hypothetical protein, conserved	TcCLB.510885.50	hypothetical protein, conserved	TcCLB.506529.150	hypothetical protein, conserved	TcCLB.510885.60	hypothetical protein, conserved
	175	TcCLB.506529.340	Protein kinase domain, putative	TcCLB.510889.30	kinase, putative	TcCLB.506529.360	cytochrome c oxidase subunit IV, putative	TcCLB.510889.50	cytochrome c oxidase subunit IV, putative
	176	TcCLB.506529.380	serine/threonine protein phosphatase, putative	TcCLB.510889.80	serine/threonine protein phosphatase, putative	TcCLB.506529.400	hypothetical protein, conserved	TcCLB.510889.100	hypothetical protein, conserved
	189	TcCLB.506529.610	hypothetical protein	TcCLB.510889.290	hypothetical protein	TcCLB.506529.620	hypothetical protein	TcCLB.510889.300	hypothetical protein

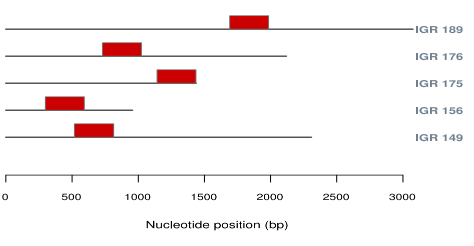

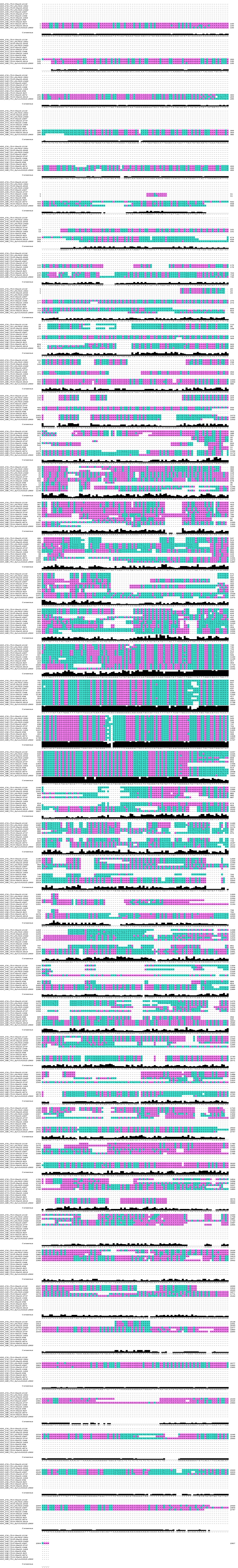

Motif #7

Motif #	IGR ID	CDS A				CDS B			
		TcII-like		TcIII-like		TcII-like		TcIII-like	
		CDS ID	Product Description	CDS ID	Product Description	CDS ID	Product Description	CDS ID	Product Description
7	973	TcCLB.510283.160	hypothetical protein, conserved	TcCLB.503823.120	hypothetical protein, conserved	TcCLB.510285.20	hypothetical protein, conserved	TcCLB.503823.130	hypothetical protein, conserved
	982	TcCLB.510285.100	eukaryotic translation initation factor 4 gamma, putative	TcCLB.504827.130	eukaryotic translation initation factor 4 gamma, putative	TcCLB.510285.110	hypothetical protein, conserved	TcCLB.504827.120	hypothetical protein, conserved

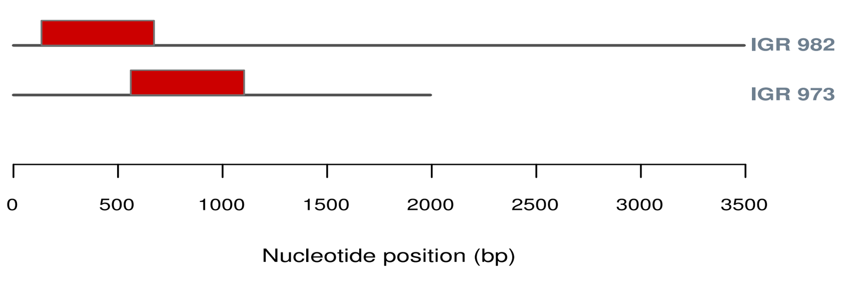

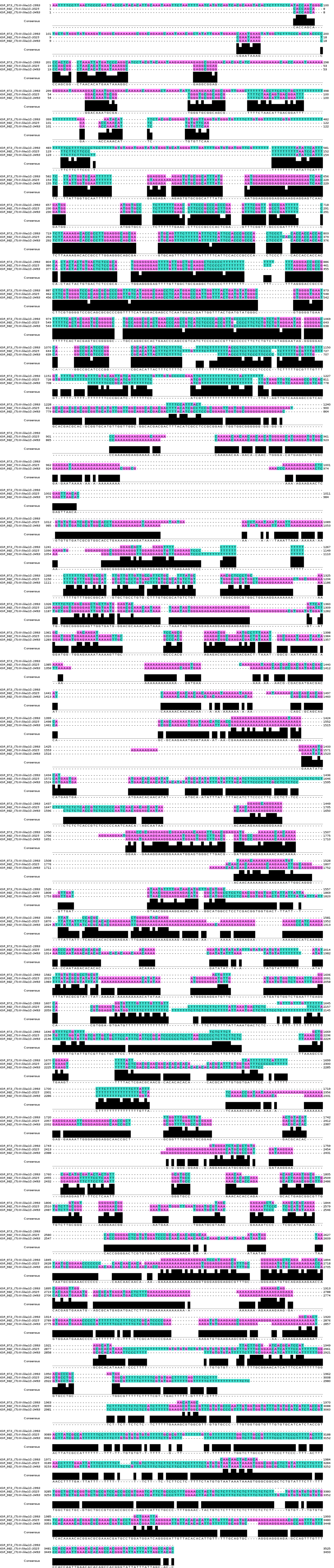

Motif #8

Motif #	IGR ID	CDS A				CDS B			
		TcII-like		TcIII-like		TcII-like		TcIII-like	
		CDS ID	Product Description	CDS ID	Product Description	CDS ID	Product Description	CDS ID	Product Description
8	933	TcCLB.510719.60	biotin/lipoate protein ligase, putative	TcCLB.509749.10	biotin/lipoate protein ligase, putative	TcCLB.510719.70	hypothetical protein, conserved	TcCLB.509747.110	hypothetical protein, conserved
	949	TcCLB.510719.220	hypothetical protein	TcCLB.509745.20	glycosyl transferase-like, putative	TcCLB.510719.230	hypothetical protein, conserved	TcCLB.509745.10	hypothetical protein, conserved

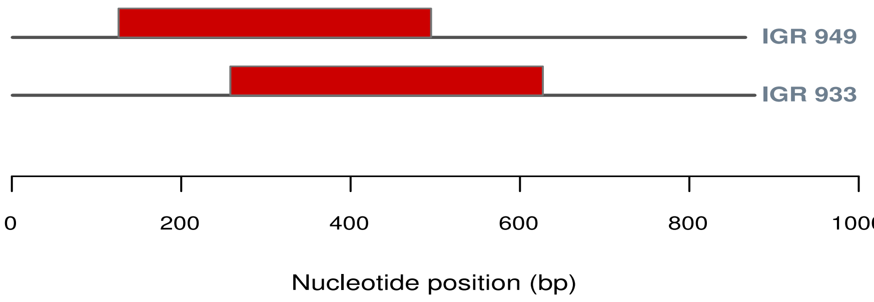

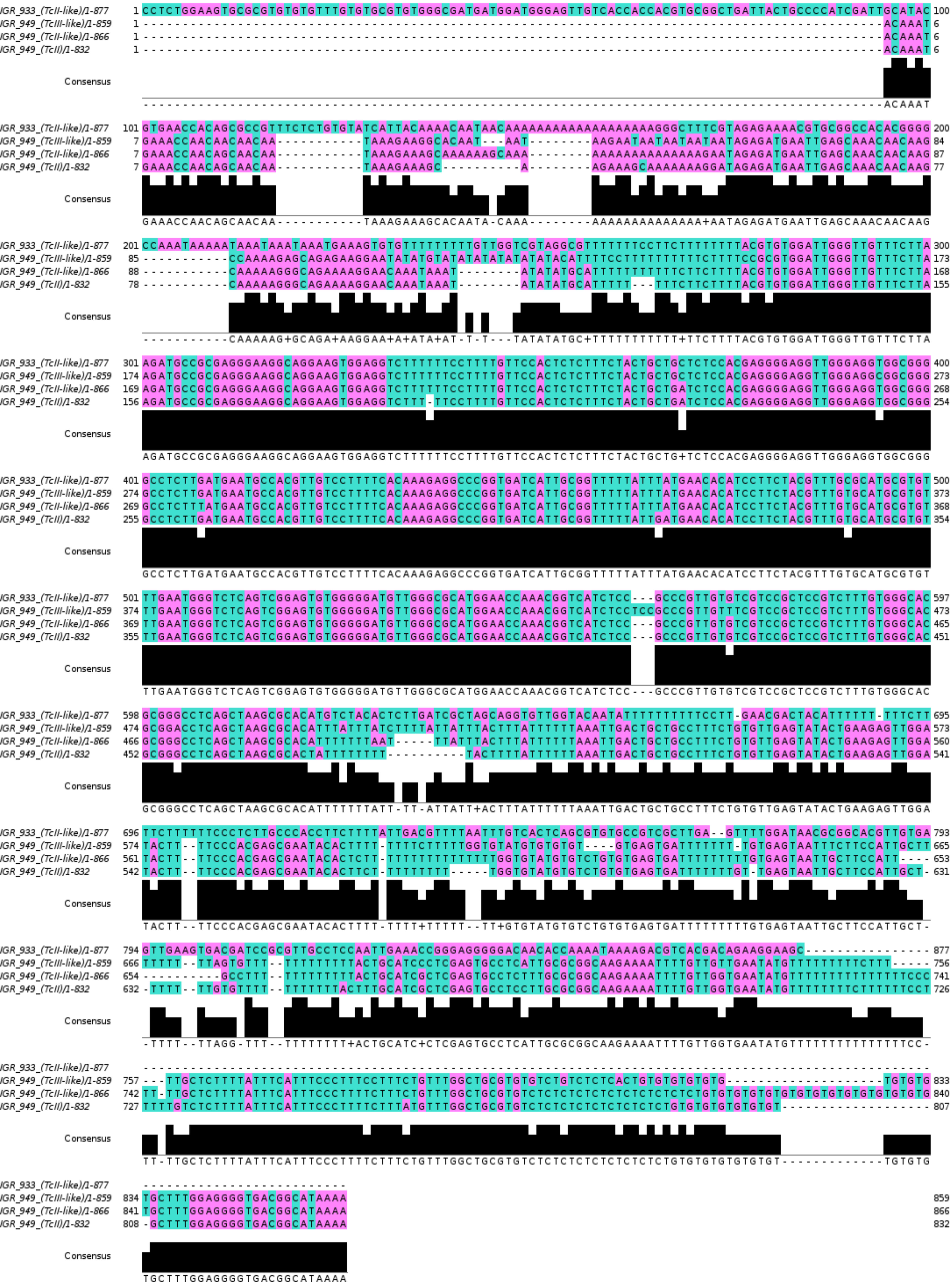

Motif #9

Motif #	IGR ID	CDS A				CDS B			
		TcII-like		TcIII-like		TcII-like		TcIII-like	
		CDS ID	Product Description	CDS ID	Product Description	CDS ID	Product Description	CDS ID	Product Description
9	1208	TcCLB.503715.10	splicing factor TSR1, putative	TcCLB.509607.30	splicing factor TSR1, putative	TcCLB.508569.10	hypothetical protein, conserved	TcCLB.509607.20	hypothetical protein, conserved
	1211	TcCLB.508569.70	hypothetical protein, conserved	TcCLB.503733.80	hypothetical protein, conserved	TcCLB.508569.80	phosphoacetylglucosamine mutase	TcCLB.503733.70	phosphoacetylglucosamine mutase, putative

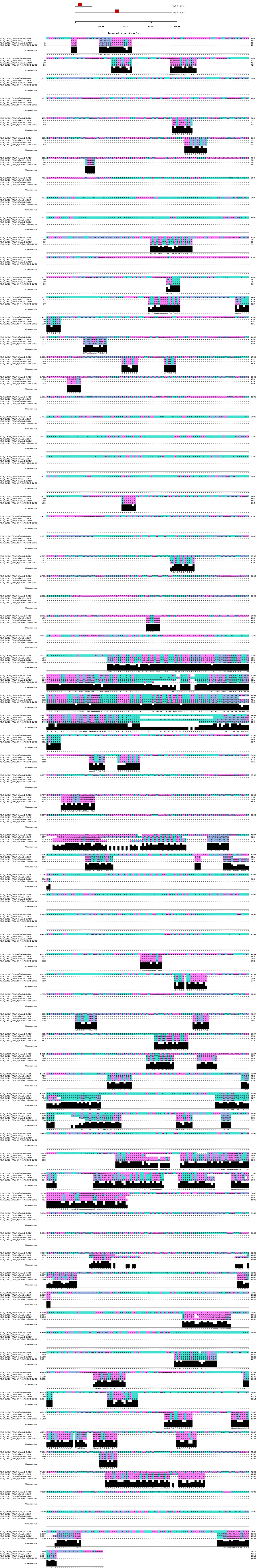

Motif #10

Motif #	IGR ID	CDS A				CDS B			
		TcII-like		TcIII-like		TcII-like		TcIII-like	
		CDS ID	Product Description	CDS ID	Product Description	CDS ID	Product Description	CDS ID	Product Description
10	1292	TcCLB.508169.90	eukaryotic translation initiation factor 3 subunit L, putative	TcCLB.506581.30	eukaryotic translation initiation factor 3 subunit L, putative	TcCLB.508169.100	hypothetical protein, conserved	TcCLB.506581.40	hypothetical protein, conserved
	1305	TcCLB.510289.30	phosphatidylinositol-4-phosphate 5-kinase, putative	TcCLB.503891.30	phosphatidylinositol-4-phosphate 5-kinase, putative	TcCLB.510289.40	GPI inositol deacylase precursor, putative	TcCLB.510967.10	GPI inositol deacylase precursor, putative

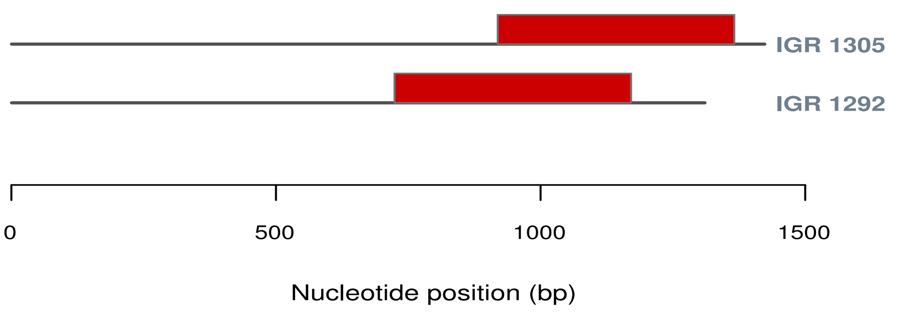

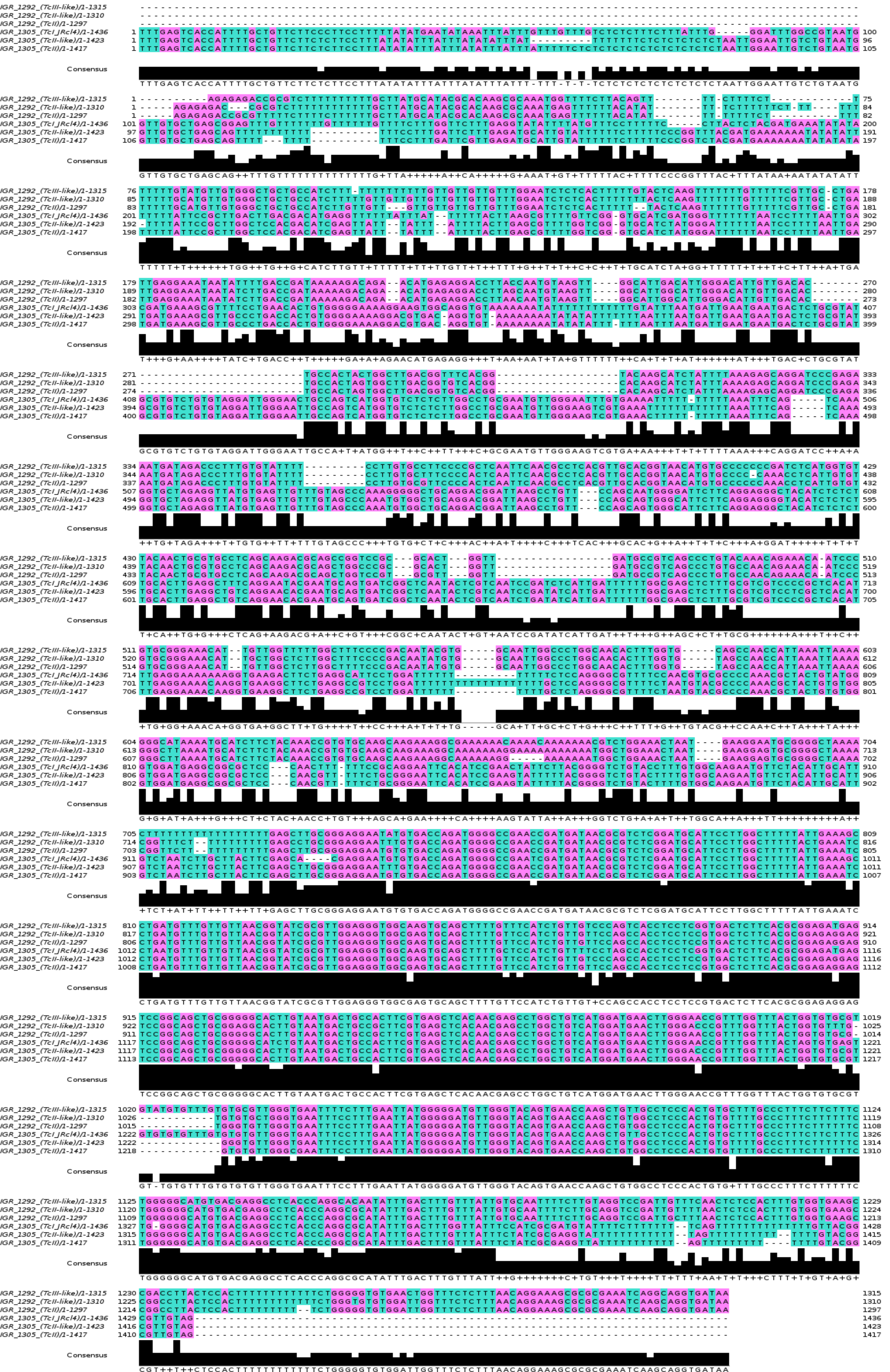

Motif #11

Motif #	IGR ID	CDS A				CDS B			
		TcII-like		TcIII-like		TcII-like		TcIII-like	
		CDS ID	Product Description	CDS ID	Product Description	CDS ID	Product Description	CDS ID	Product Description
11	2449	TcCLB.505965.50	phosphonopyruvate decarboxylase, putative	TcCLB.510903.50	phosphonopyruvate decarboxylase, putative	TcCLB.505965.60	hypothetical protein, conserved	TcCLB.510903.40	hypothetical protein, conserved
	7897	No_ortholog		TcCLB.510901.250	hypothetical protein, conserved	No_ortholog		TcCLB.510901.230	hypothetical protein, conserved

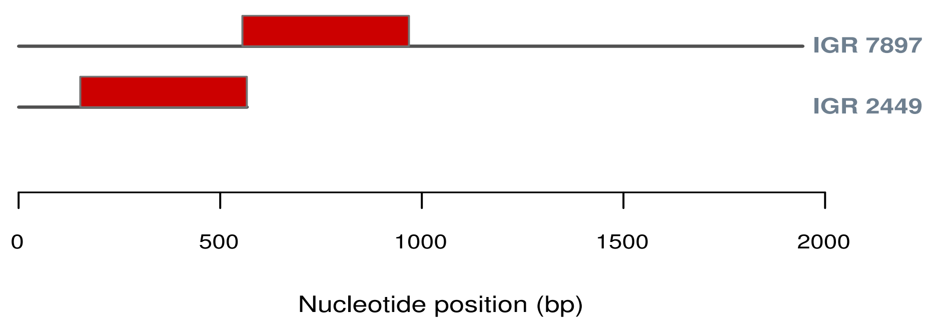

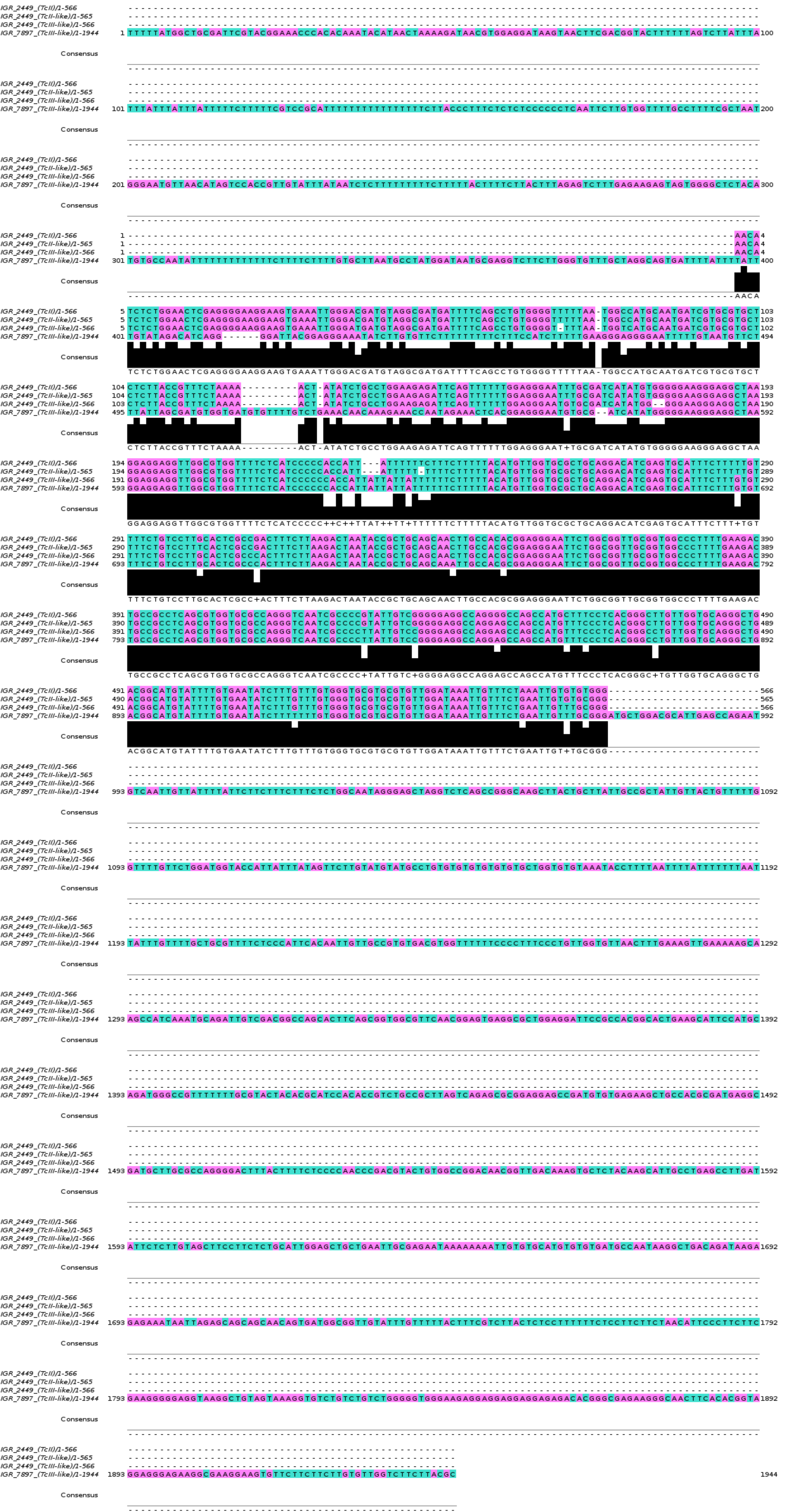

Motif #12

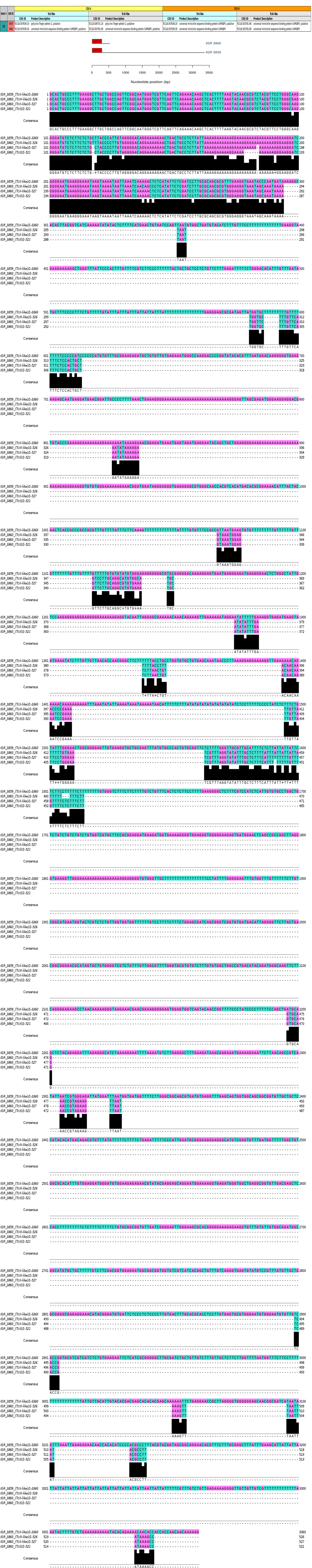

Motif #13

Motif #	IGR ID	CDS A				CDS B			
		TcII-like		TcIII-like		TcII-like		TcIII-like	
		CDS ID	Product Description	CDS ID	Product Description	CDS ID	Product Description	CDS ID	Product Description
13	4004	TcCLB.511445.140	hypothetical protein	TcCLB.503703.60	hypothetical protein	TcCLB.511445.160	copper-transporting ATPase-like protein, putative	TcCLB.503703.50	copper-transporting ATPase-like protein, putative
	8957	TcCLB.511445.160	copper-transporting ATPase-like protein, putative	TcCLB.503703.50	copper-transporting ATPase-like protein, putative	TcCLB.503893.30	hypothetical protein, conserved	TcCLB.503703.40	hypothetical protein, conserved

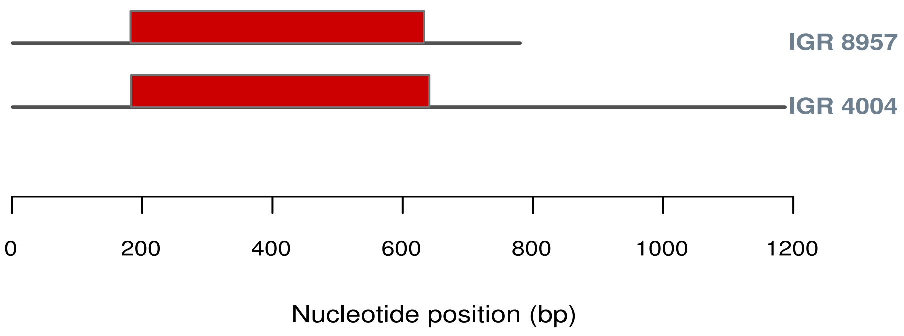

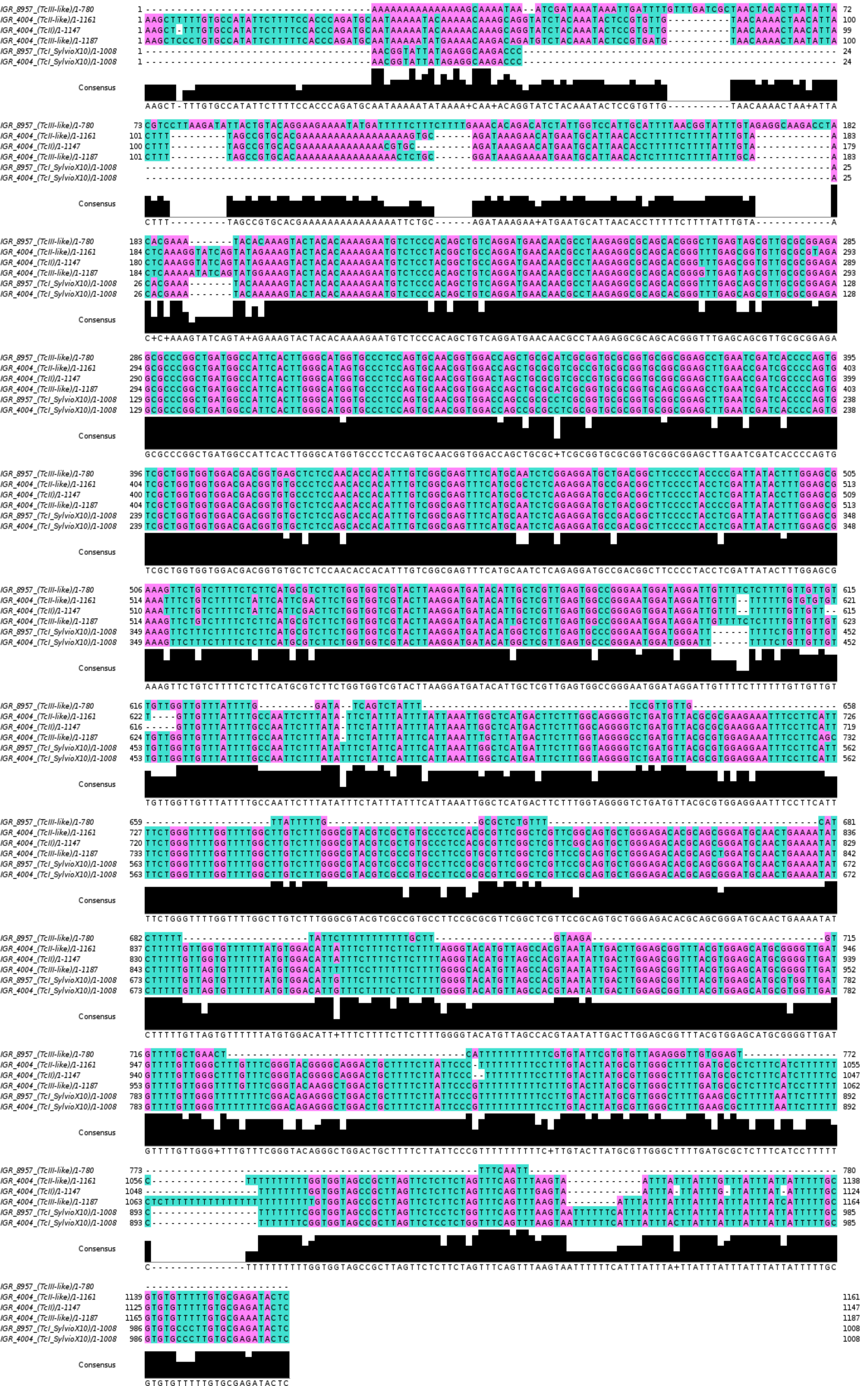

Motif #14

Motif #	IGR ID	CDS A				CDS B			
		TcII-like		TcIII-like		TcII-like		TcIII-like	
		CDS ID	Product Description	CDS ID	Product Description	CDS ID	Product Description	CDS ID	Product Description
14	4012	TcCLB.503893.100	hypothetical protein, conserved	TcCLB.508507.40	hypothetical protein, conserved	TcCLB.503893.120	calcium uniporter protein, mitochondrial, putative	TcCLB.508507.30	calcium uniporter protein, mitochondrial, putative
	4036	TcCLB.509831.20	vesicular-fusion ATPase-like protein, putative	TcCLB.506629.60	vesicular-fusion ATPase-like protein, putative	TcCLB.509831.30	hypothetical protein, conserved	TcCLB.506629.50	hypothetical protein, conserved

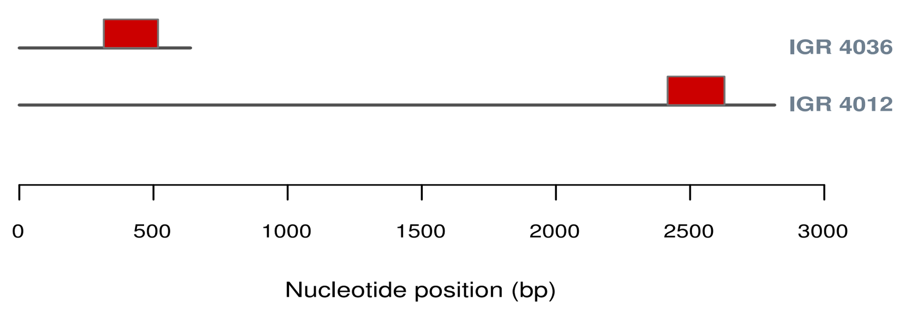

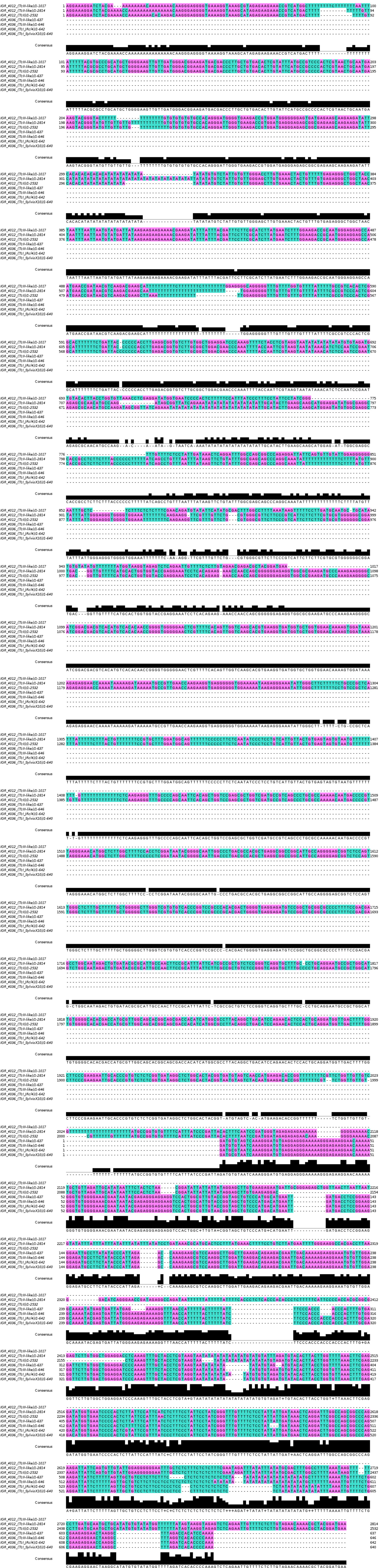

Motif #15

Motif #	IGR ID	CDS A				CDS B			
		TcII-like		TcIII-like		TcII-like		TcIII-like	
		CDS ID	Product Description	CDS ID	Product Description	CDS ID	Product Description	CDS ID	Product Description
15	4425	TcCLB.507683.34	hypothetical protein, conserved	TcCLB.511291.50	hypothetical protein, conserved	TcCLB.507683.40	protein kinase, putative	TcCLB.511291.40	protein kinase, putative
	6735	TcCLB.506211.114	C-terminal motor kinesin, putative	TcCLB.510901.50	C-terminal motor kinesin, putative	TcCLB.506211.120	hypothetical protein, conserved	TcCLB.511289.110	hypothetical protein, conserved

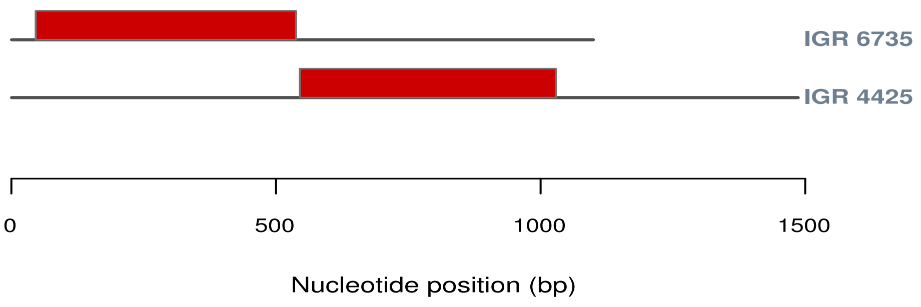

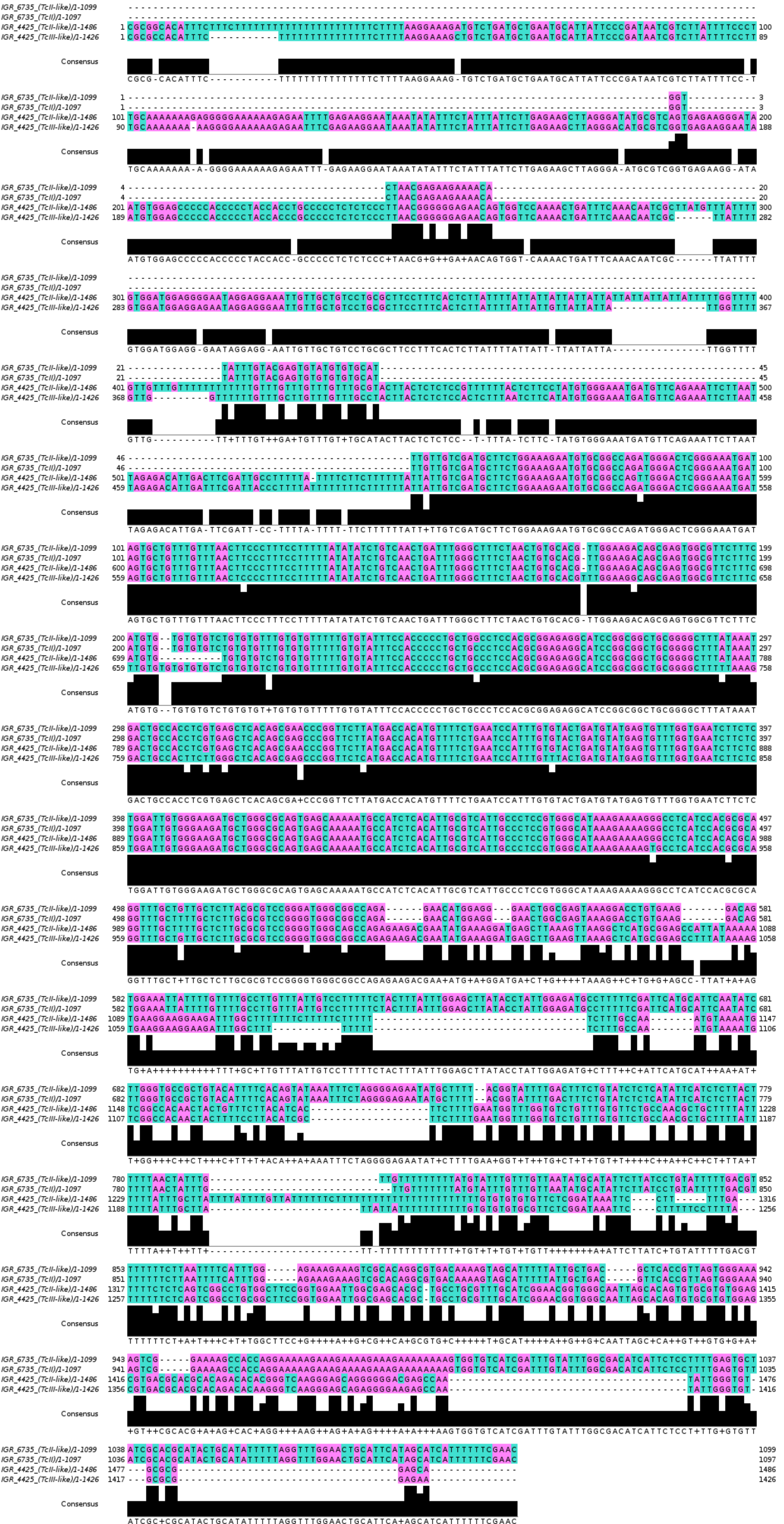

Motif #16

Motif #	IGR ID	CDS A				CDS B			
		TcII-like		TcIII-like		TcII-like		TcIII-like	
		CDS ID	Product Description	CDS ID	Product Description	CDS ID	Product Description	CDS ID	Product Description
16	6481	TcCLB.506925.70	epsin, putative	No_ortholog	-	TcCLB.506925.80	hypothetical protein, conserved	No_ortholog	-
	6514	TcCLB.506925.480	ribokinase, putative	No_ortholog	-	TcCLB.506925.490	hypothetical protein, conserved	No_ortholog	-

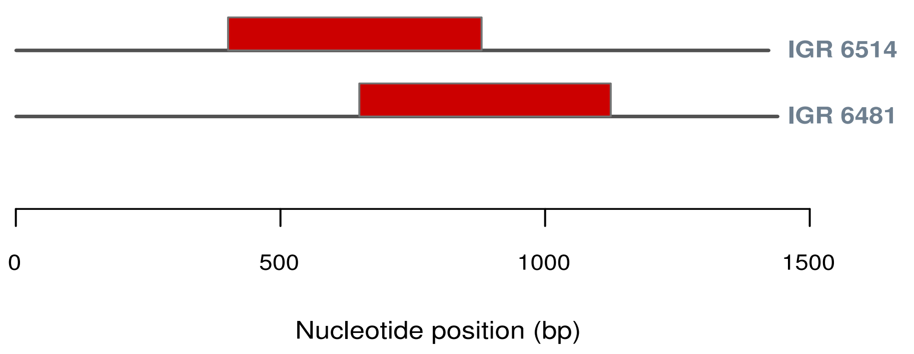

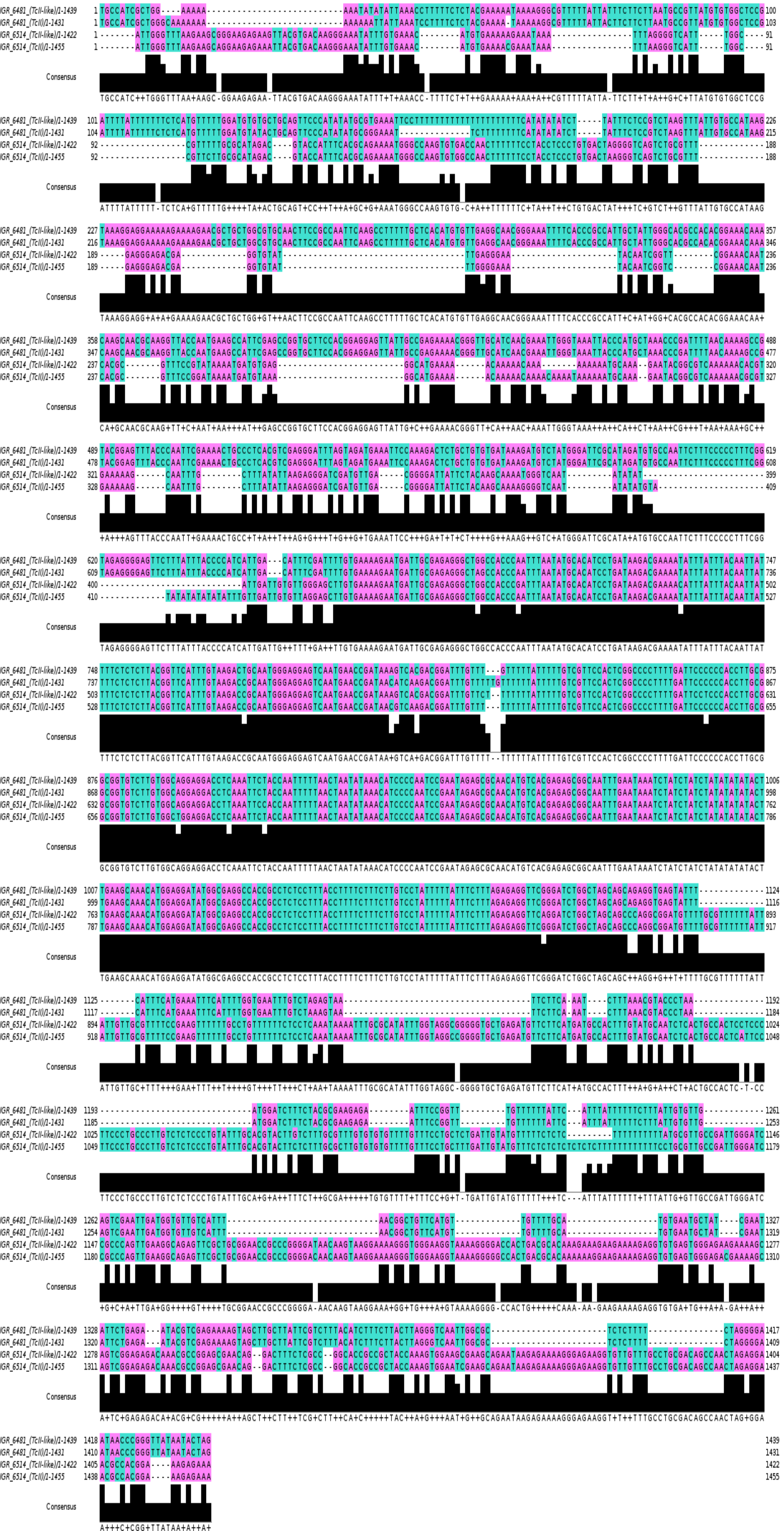

Motif #17

Motif #	IGR ID	CDS A				CDS B			
		TcII-like		TcIII-like		TcII-like		TcIII-like	
		CDS ID	Product Description	CDS ID	Product Description	CDS ID	Product Description	CDS ID	Product Description
17	6483	TcCLB.506925.90	hypothetical protein, conserved	No_ortholog	-	TcCLB.506925.100	hypothetical protein, conserved	No_ortholog	-
	6492	TcCLB.506925.170	hypothetical protein, conserved	No_ortholog	-	TcCLB.506925.180	protein kinase, putative	No_ortholog	-

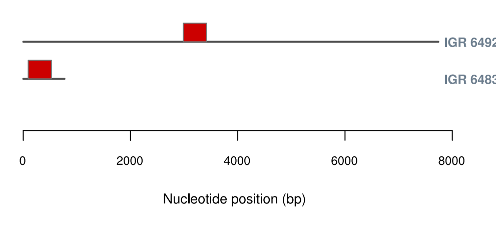

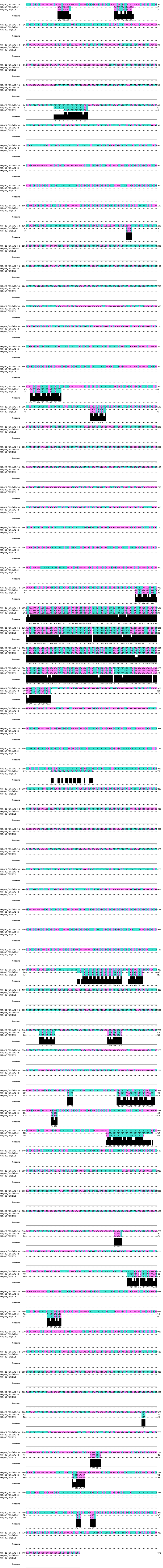

Motif #18

Motif #	IGR ID	CDS A				CDS B			
		TcII-like		TcIII-like		TcII-like		TcIII-like	
		CDS ID	Product Description	CDS ID	Product Description	CDS ID	Product Description	CDS ID	Product Description
18	7224	No_ortholog	-	TcCLB.511807.160	hypothetical protein, conserved	No_ortholog	-	TcCLB.511807.180	hypothetical protein, conserved
	7225	No_ortholog	-	TcCLB.511807.180	hypothetical protein, conserved	No_ortholog	-	TcCLB.511807.190	hypothetical protein, conserved

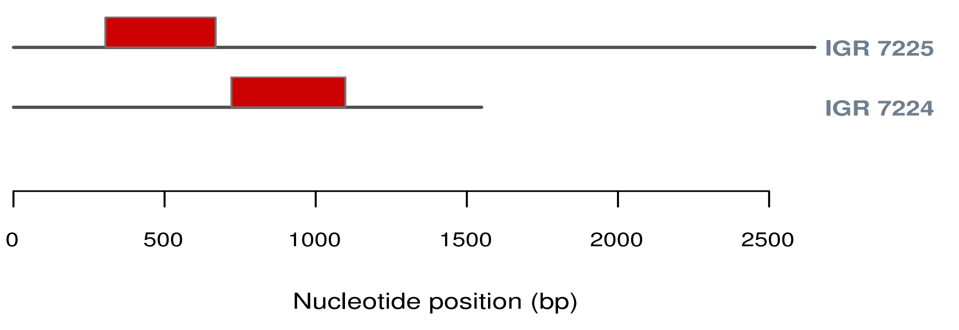

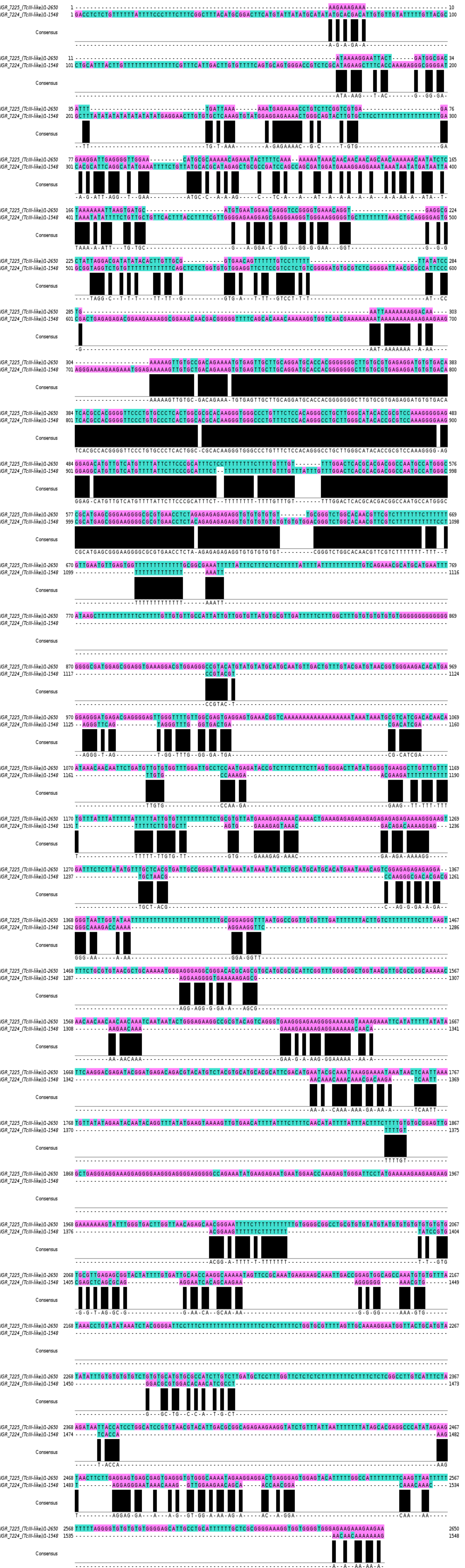

Motif #19

Motif #	IGR ID	CDS A				CDS B			
		TcII-like		TcIII-like		TcII-like		TcIII-like	
		CDS ID	Product Description	CDS ID	Product Description	CDS ID	Product Description	CDS ID	Product Description
19	3827	TcCLB.507677.150	CYC2-like cyclin, putative,G1 cyclin CycE4, putative	TcCLB.504021.10	CYC2-like cyclin, putative,G1 cyclin CycE4, putative	TcCLB.507677.140	hypothetical protein, conserved	TcCLB.504021.20	hypothetical protein, conserved
	8824	No_ortholog		TcCLB.509647.150	calcium-transporting ATPase, putative	No_ortholog		TcCLB.509647.140	hypothetical protein, conserved

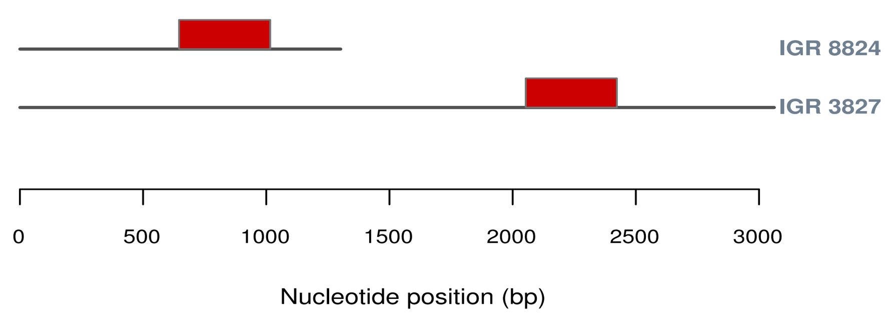

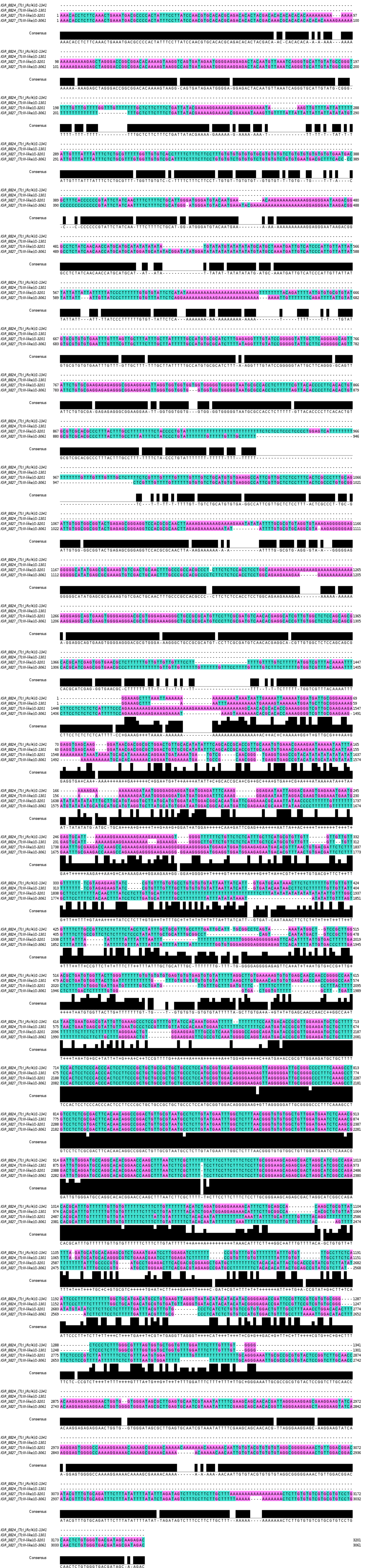

Motif #20

Motif #	IGR ID	CDS A				CDS B			
		TcII-like		TcIII-like		TcII-like		TcIII-like	
		CDS ID	Product Description	CDS ID	Product Description	CDS ID	Product Description	CDS ID	Product Description
20	8880	No_ortholog	-	TcCLB.510187.270	COP-coated vesicle membrane protein erv25 precursor, putative	No_ortholog	-	TcCLB.510187.260	hypothetical protein, conserved
	8881	No_ortholog	-	TcCLB.510187.260	hypothetical protein, conserved	No_ortholog	-	TcCLB.510187.250	hypothetical protein, conserved

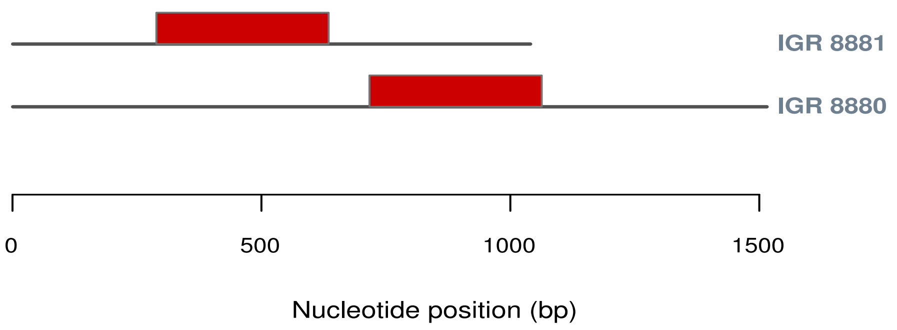

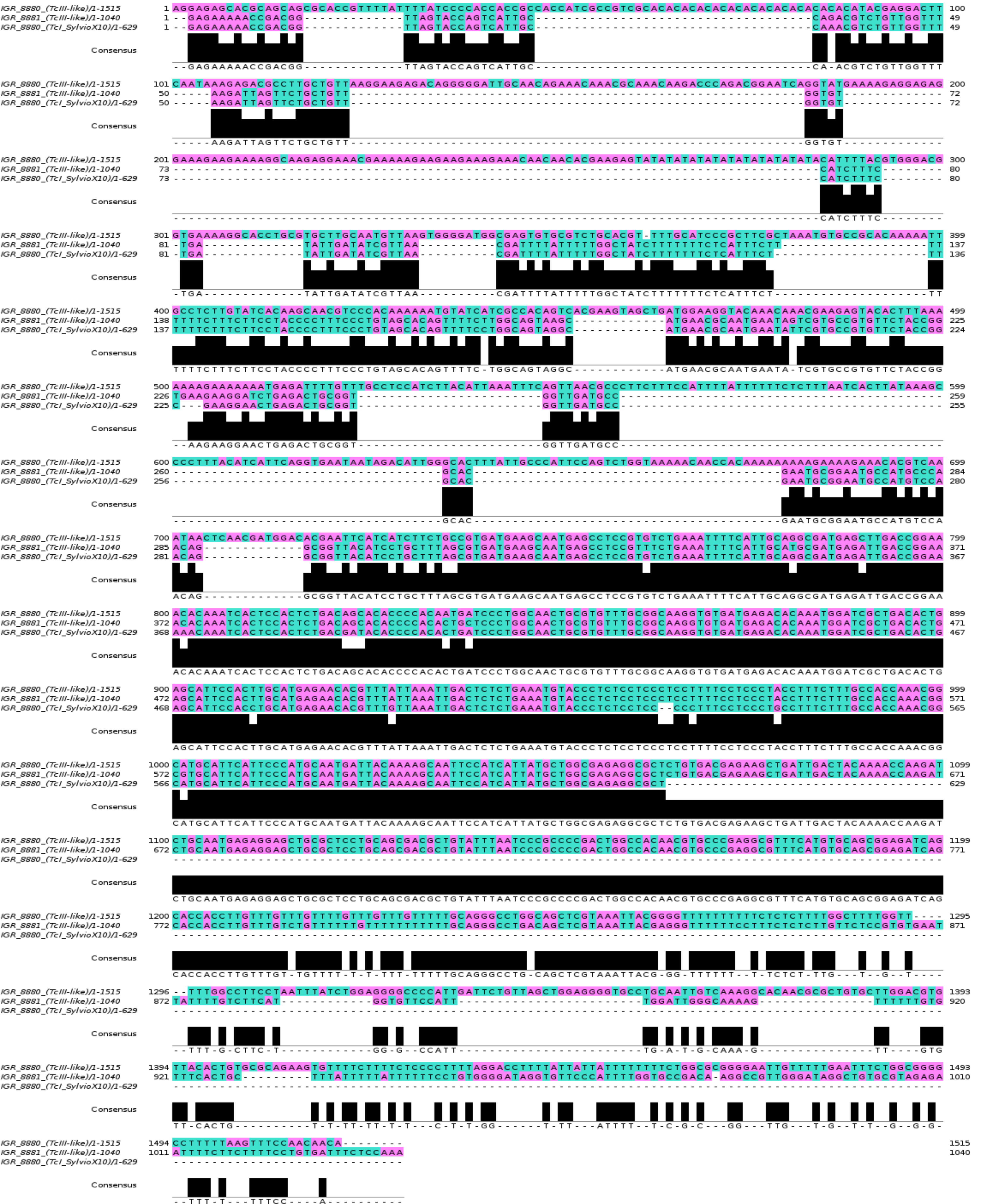

Motif #21

Motif #	IGR ID	CDS A				CDS B			
		TcII-like		TcIII-like		TcII-like		TcIII-like	
		CDS ID	Product Description	CDS ID	Product Description	CDS ID	Product Description	CDS ID	Product Description
21	4172	TcCLB.506147.180	DNA polymerase epsilon catalytic subunit, putative	TcCLB.510733.60	DNA polymerase epsilon catalytic subunit, putative	TcCLB.506147.170	hypothetical protein, conserved	TcCLB.510735.9	hypothetical protein, conserved
	6637	TcCLB.509551.110	hypothetical protein, conserved	TcCLB.507007.34	hypothetical protein, conserved	TcCLB.509551.100	hypothetical protein, conserved	TcCLB.507007.40	hypothetical protein, conserved

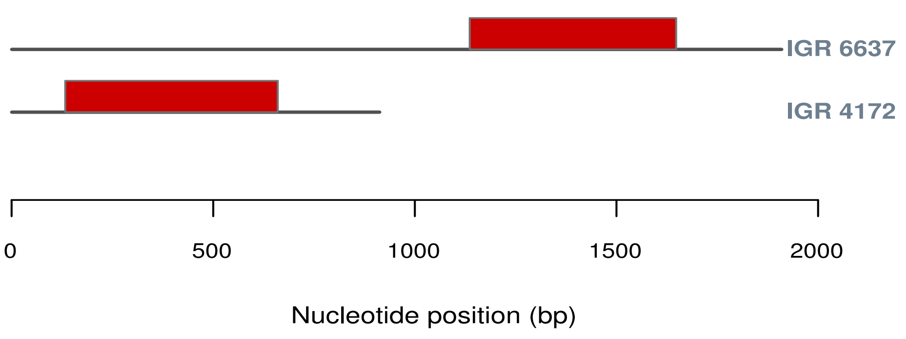

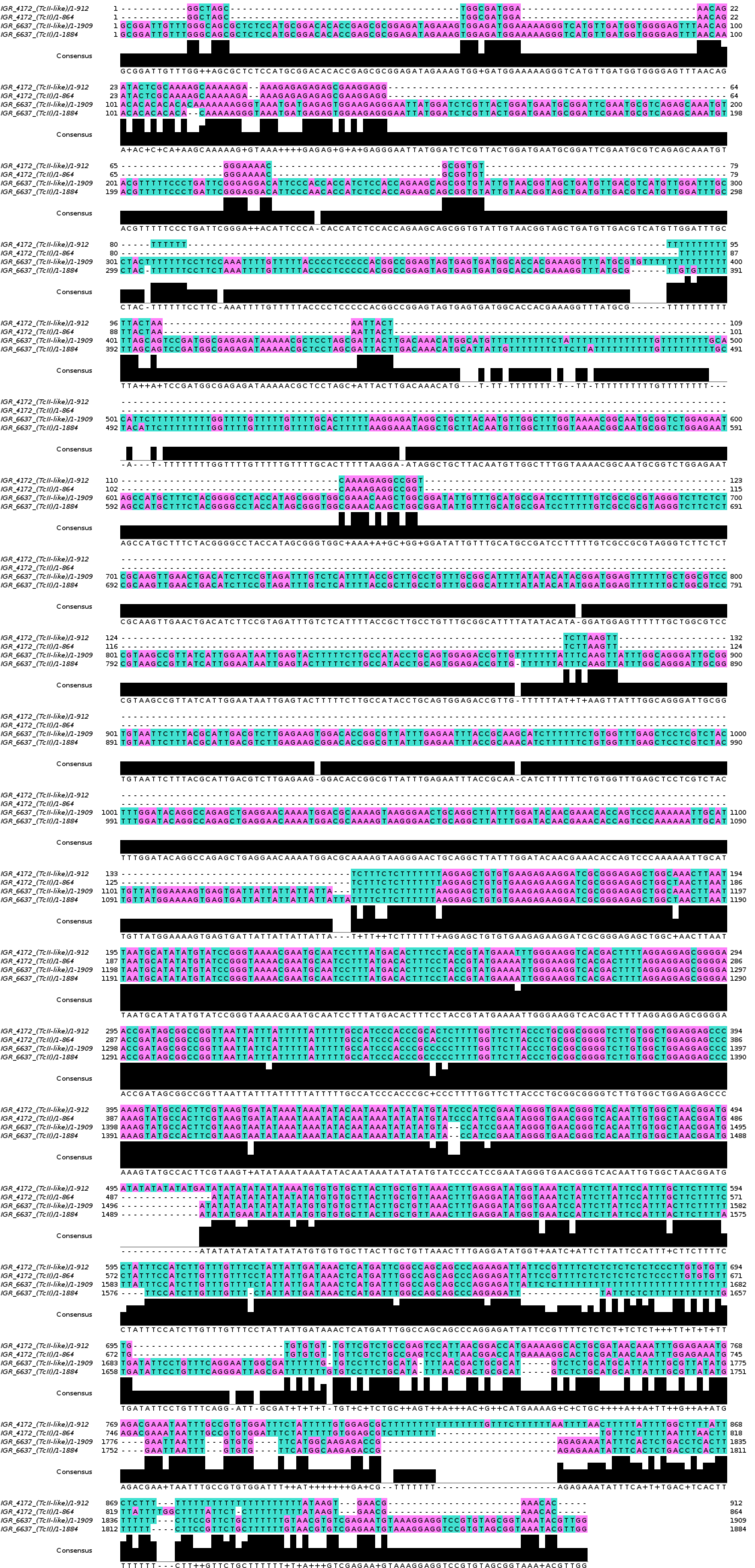

Supplement: Dataset S1 — Intergenic regions from unrelated loci that share blocks of significant sequence similarity. The file contains i) a spreadsheet summarizing listing the non-allelic, non-homologous IGR regions that share significant blocks of sequence similarity; ii) multiple sequence alignments where these unrelated IGRs were aligned to highlight the portion of the IGR that is shared; and iii) a figure in PDF format that shows the alignment in context. The alignments are provided in CLUSTAL format (.clw) and as colored renderings in PNG format, as produced by Jalview (purine/pyrimidine color scheme, only applied to regions of the alignment with 90% identity). The IGR IDs listed in each alignment correspond to those in Table S3. (ZIP) [file pntd.0002839.s010.zip › Dataset S1/Figure-D1a.pdf]
