# Supplementary material for: A Genome-Wide Analysis of Genetic Diversity in Trypanosoma cruzi Intergenic Regions
Source: PLoS Negl Trop Dis. 2014 May 1;8(5):e2839. doi: 10.1371/journal.pntd.0002839 (PMC4006747; doi:10.1371/journal.pntd.0002839)
Supplement: Dataset S1 — Intergenic regions from unrelated loci that share blocks of significant sequence similarity. The file contains i) a spreadsheet summarizing listing the non-allelic, non-homologous IGR regions that share significant blocks of sequence similarity; ii) multiple sequence alignments where these unrelated IGRs were aligned to highlight the portion of the IGR that is shared; and iii) a figure in PDF format that shows the alignment in context. The alignments are provided in CLUSTAL format (.clw) and as colored renderings in PNG format, as produced by Jalview (purine/pyrimidine color scheme, only applied to regions of the alignment with 90% identity). The IGR IDs listed in each alignment correspond to those in Table S3. (ZIP) [file pntd.0002839.s010.zip › Dataset S1/README-S1.pdf]

DATASET S1, Panunzi LG and Agüero F (2014)

This zipfile contains a number of files and folders that provide additional information on a set of 21 conserved sequence elements that are shared by a number of non-allelic, non-homologous intergenic regions in T. cruzi.

List of files:

1. README-S1.pdf (this file)

.

2. Table-D1.xls, an excel spreadsheet that provides information on the groups of IGRs that share each of the 21 sequence elements, their flanking CDS sequences, genomic location, current annotation (including GO annotation), and the number of orthologs and paralogs (which provides information on the phylogenetic spread of each CDS).

3. Figure-D1a.pdf, is a PDF file that contains a summary of the information available for each shared element within the complete IGR: i) a snapshot of the information available in Table-D1, a graphic visualization of the locations of shared elements among different IGRs, and a graphic rendering of a multiple sequence alignment of the corresponding IGRs. Each page within the PDF file has its own custom size. You need to adjust the zoom for each page accordingly.

4. Figure-D1b.pdf, is a PDF file that contains a multiple sequence alignment of every single shared element among different IGRs. Each page within the PDF file has its own custom size. You need to adjust the zoom for each page accordingly.

Each of the the following folders includes two sub-folders, named “Entire IGRs” and “Only motifs”, enclosing files of whole IGRs sequences and files of sole sequence elements, respectively.

5. Alignments (folder). Each sub-folder includes 21 alignments in CLUSTAL format. These files can be opened in Jalview/Seaview (your alignment editor of choice) for further manipulation.

6. Alignment pictures (folder). Each sub-folder comprises 21 pictures of the alignments. These renderings were generated by Jalview. Coloring is by type of base (purines vs pyrimidines).

7. Sequences (folder). Each sub-folder contains the FASTA files with the sequence of the conserved element in each IGR.
